# One-Step Regioselective Synthesis of Benzofurans from Phenols and *α*-Haloketones

**DOI:** 10.3390/molecules24112187

**Published:** 2019-06-11

**Authors:** Bingqiao Wang, Qiu Zhang, Juan Luo, Zongjie Gan, Wengao Jiang, Qiang Tang

**Affiliations:** College of Pharmacy, Center for Lab Teaching and Management, Chongqing Medical University, No.1 Yixueyuan Road, Chongqing 400016, China; wwangkkang111@163.com (B.W.); zq77668811@163.com (Q.Z.); 102693@cqmu.edu.cn (J.L.); gzj@cqmu.edu.cn (Z.G.); bluesky_gx@sina.com (W.J.)

**Keywords:** naphthofuran, benzofuran, titanium tetrachloride, *α*-haloketones, cyclodehydration

## Abstract

Reported here is the direct synthesis of naphthofurans and benzofurans from readily available phenols and *α*-haloketones promoted by titanium tetrachloride which combines Friedel–Crafts-like alkylation and intramolecular cyclodehydration into one step. This simple protocol allows for the formation of a variety of high value naphthofurans and benzofurans within which a series of cyclic and acyclic groups are readily incorporated. This process demonstrates the advantages of high levels of regioselectivity, broad substrate scope, and moderate to excellent yields.

## 1. Introduction

**Figure 1:**
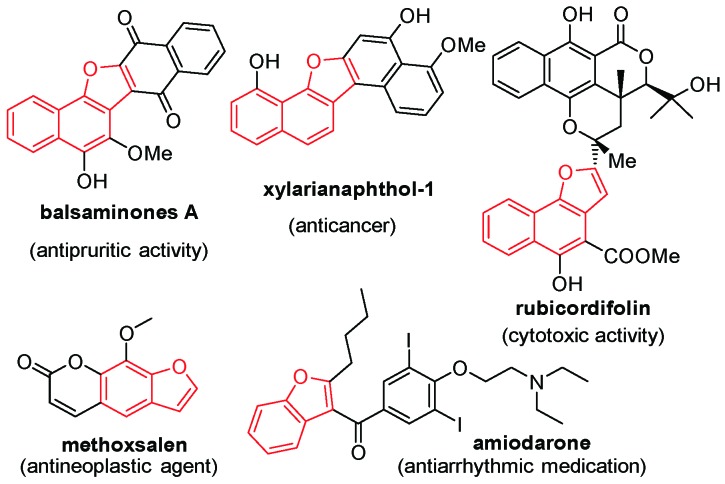
Representative drugs containing benzofuran core.

Benzofuran derivatives, especially naphthofurans, constitute a valuable class of heterocyclic compounds due to their natural occurrence and remarkable biological activities [1,2]. Currently, more than 30 drugs bearing a benzofuran moiety have been approved by the United States Food and Drug Administration (USFDA) [3,4]. Furthermore, naphthofurans have attracted significant attention in recent years owing to their powerful paradigm in the development and design of potential anticancer drugs [5], dual inhibitors of Alzheimer’s disease [6,7], inhibitors of human protein kinase [8], and regulators of the nuclear receptor [9], as well as other bioactivities [10,11]. Several representative bioactive compounds possessing a naphthofuran or benzofuran skeleton are listed in Figure 1 [12,13]. Therefore, the development of novel synthetic methods for their direct preparation from readily accessible materials is very important.

**Scheme 1:**
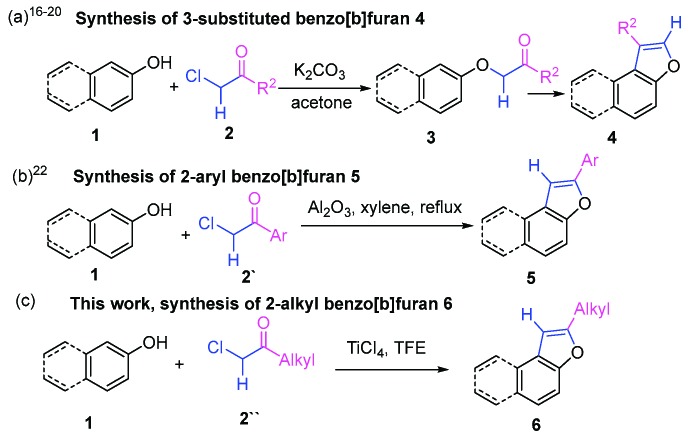
Synthesis of benzofurans from *α*-haloketones and phenols.

Because of the aforementioned importance, numerous approaches have been reported for the preparation of these scaffolds through transition-metal catalysis, Lewis or Brønsted acid catalyzed, or base-promoted cyclizations [14,15]. Many of these methods rely on harsh conditions, expensive transition metals, or substrates that are difficult to obtain. The strategy using phenols and α-haloketones as starting materials to obtain benzofurans is one of the most convenient routes. 3-Substituted benzo[b]furan **4** can be easily synthesized by a stepwise [16,17,18] or a one-step method [19,20] which involves *o*-alkylation of simple phenols with α-haloketone followed by intramolecular cyclization (Scheme 1a). However, there are seldom reports concerned with the synthesis of 2-substituted benzo[b]furans using *α*-haloketone and phenols as starting materials [21]. Recently, Arias et al. have reported that 2-aryl benzo[b]furan **5** can be obtained with excellent regioselectivity under refluxing temperature using neutral alumina as a promoter and xylene as a solvent [22]. Nevertheless, the scope of *α*-haloketone is limited to only aryl ketone, without any alkyl ketone being employed (Scheme 1b). To continue our research [18], we report here that 2-alkyl benzo[b]furan **6** can be regioselectively formed directly from *α*-haloketones and phenols in the presence of titanium tetrachloride (Scheme 1c).

## 2. Results and Discussion

In order to develop a concise approach to naphthofuran, 2-naphthol (**1a**) and 2-chloroacetone (**2a**) were selected as model substrates (Table 1). To our delight, the reaction took place smoothly and proceeded to completion in ten hours when titanium tetrachloride was used in the presence of trifluoroethanol (TFE). The desired product **6a** (Table 1) was formed regioselectively, without any other isomer being detected (entry 8).

As a matter of fact, no conversion to the desired product was observed when commonly used Brønsted acids or other Lewis acids were tested (Table 1, entries 1–4). When TMSOTf or BF_3_.Et_2_O was used, the reaction produced numerous by-products and finally provided only a few furan products (Table 1, entries 5 and 6). Using reaction conditions reported in the literature [22], the reaction did take place but produced an inseparable mixture of **4a** and **6a** (Table 1, entry 7). Moreover, it was discovered that increasing or decreasing the amount of titanium tetrachloride led to lower reaction efficiency (Table 1, entries 9–11). Note that slightly higher reaction temperature is beneficial to both reaction rate and efficiency. Actually, only a trace amount of **6a** was detected by TLC when the reaction mixture was stirred at room temperature overnight, and the reaction turned out to be complex if prolonging the reaction time (Table 1, entry 13). The reaction could be carried out in several conventional solvents (CH_2_Cl_2_ and toluene) in addition to TFE, although resulting in significantly diminished conversions and a longer reaction time (Table 1, entries 14 and 15). Other solvents (CH_3_CN, Et_2_O and THF) were also screened at their refluxing temperatures, but no new product could be detected after stirring overnight (Table 1, entry 16). The effect of catalyst amount and reaction temperature in this reaction was then investigated.

With an optimal set of catalysis conditions selected, we were then poised to test the one-pot process and evaluate the substrate scope of this reaction. When the reaction was conducted in refluxing TFE in the presence of titanium tetrachloride, we were delighted to find that both 1- and 2-naphthols functioned efficiently in the reaction with 2-chloroacetone, with nearly single isomer being isolated (Table 2, **6a**–**6d**, 99:1 rr). The yields and reaction rates for 1-naphthol, in general, were a little better than those of 2-naphthol. Bromo-substituted naphthols were also highly effective regardless of the position of the bromo group on the phenyl ring (Table 2, **6c** and **6d**, 89% and 66% yields, respectively). Additionally, simple 3-chloro-2-butanone was also highly effective in the current protocol (Table 2, **6e** and **6f**, 74% and 76% yields, respectively).

Regioisomers were obtained in the reactions of other acyclic α-haloketones (Table 2, **6g**–**6l**). The phenomenon of isomerization was particularly obvious for the reaction of 2-chloro-3-pentanone stirred at room temperature (Table 2, **6g**, 2:1 rr). However, the problem caused by isomerization was readily overcome by slightly raising the reaction temperature and dropping *α*-haloketones into the reaction mixture. By employing the above-mentioned procedures, the desired products were afforded with high regioselectivity and good yields (Table 2, **6g**, 9:1 rr; **6h**, 10:1 rr).

We next examined the scope of cyclic α-chloroketone which finally produced furans with four cycles (Table 3). It was gratifying to find that these reactions were completed in 3–10 h to afford the corresponding tetracyclic products with moderate to excellent yields. Importantly, the transformation is not limited to six-membered cyclic *α*-chloroketone, as five-, seven-, and eight-membered cyclic *α*-chloroketones are competent substrates. Interestingly, both reaction rate and yield for six-membered cyclic α-chloroketone (Table 3, **7b**, **7e**, **7i**, and 7**m**) were better than those of other cyclic α-chloroketones. Intriguingly, all the reactions of 1-naphthol with cyclic α-chloroketones proceeded to completion in 3 h, offering products with excellent yields (Table 3, **7e**–**7g**). However, the reactions of bromo-substituted naphthols, such as 6-bromo or 7-bromo-2-naphthol, required longer reaction times (10–24 h) and offered only moderate yields of naphthofurans **7h**, **7j**–**7l**, and **7n**–**7o** (Table 3).

To further extend the reaction scope, we carried out the reaction with phenols. First, we used 2-chlorocyclohexanone to examine the reactivity of various substituted phenols. Gratifyingly, all alkyl- or alkoxy-substituted phenols reacted successfully with 2-chlorocyclohexanone to produce the desired benzofurans **9a**–**9h** (Table 4) with excellent yields. Additionally, it was found that the substituent patterns (ortho-, meta- and para-) on the benzene ring showed no observed effects on the reaction outcomes (Table 4, **9b**–**9d**). Note that the reaction exhibits sensitivity to steric constraints on the phenol substrate, that is, the Friedel–Crafts-like alkylation occurs preferentially at the less hindered position, which can be demonstrated by the formation of a single isomer (Table 4, **9c**). Unfortunately, phenols incorporating an electron-withdrawing group did not react under these conditions. For example, no new spot was detected by TLC when 4-nitrophenol was employed to react with 2-chlorocyclohexane for 24 h. Moreover, phenols bearing a strong electron-donating substituent, such as methoxyl, failed to give better yields (Table 4, **9e** and **9f**, 72% and 73% yields, respectively), although the reaction rates were faster than that of non-substituted phenol. On the other hand, the reactions of phenols with acyclic α-haloketones were also examined, which proceeded smoothly (Table 4, **9i**–**9k**, 77–81% yields).

**Scheme 2:**
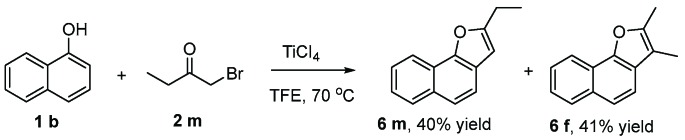
Mechanism research.

To gain insight into the reaction mechanism, we carried out the reaction with unsymmetrically substituted haloketone **2m** (Scheme 2), under the optimized conditions. Pleasingly, both the desired furan **6m** and isomer **6f** (Scheme 2) were isolated in nearly equal amounts. However, reactions for *α*-halo aromatic ketones, such as 2-bromoacetophenone and 2-bromo-1-phenylpentanone, failed to occur. These facts suggested that oxy-allyl cation may be one of the key intermediates for the reaction between *α*-halo alkyl ketones and phenols.

**Scheme 3:**
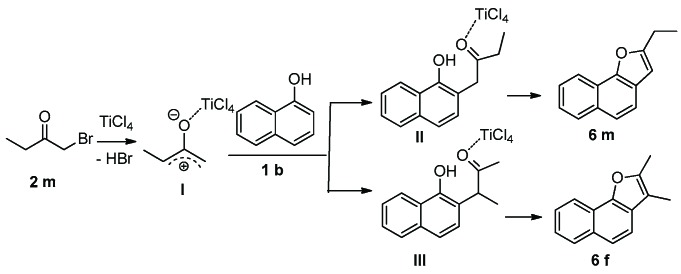
Proposed mechanism.

It is reported that azepinium ions can be generated by the ether cleavage reaction of 2-methoxy-2*H*-azepine derivatives with titanium tetrachloride as a Lewis acid [23,24]. Furthermore, titanium tetrachloride is also a powerful dehydrating agent and demonstrates a prominent effect in the condensation reaction of triketones to yield furans [25]. Apart from the reaction paths reported in the literature (Scheme 1a,b) [22], another reaction route for the one-pot synthesis of benzofuran was proposed in Scheme 3 [26,27]. First, oxy-allyl cation **I**, evolved from **2m** with the aid of titanium tetrachloride, reacts with **1b** to produce Friedel–Crafts type intermediate **II** or **III** (Scheme 3). Then, due to the powerful dehydration ability of titanium tetrachloride, intramolecular cyclodehydration of the intermediate **II** or **III** easily takes place to obtain benzofuran **6m** or **6f** (Scheme 3).

## 3. Experimental Section

### 3.1. General Information

Nuclear magnetic resonance spectra (^1^H and ^13^C) were recorded on 400 and 600 MHz spectrometers (Bruker, Karlsruhe, Germany) with tetramethylsilane (TMS) as an internal standard.The splitting patterns are designated as singlet (s), doublet (d), triplet (t), quartet (q), dd (doublet of doublets), m (multiplets), etc. All first-order splitting patterns were assigned on the basis of the appearance of the multiplet. Splitting patterns that could not be easily interpreted were designated as multiplet (m) or broad (br). High resolution mass spectral analysis (HRMS) was performed on an ESI-QTOP mass spectrometer (Bruker Solari XFT-ICR-MS system). Purification was done by column chromatography and preparative TLC using silica gel. TLC analyses were performed on commercial glass plates (Qingdao Haiyang Chemical Co., Ltd, Qingdao, China) bearing a 0.25-mmlayer of silica gel GF_254_.Visualization was performed using a UV lampor chemical stains like KMnO_4_ and I_2_. Commercially available materials were used as received.

All reactions were carried out under nitrogen atmosphere. Dehydrated solvents were purchased from commercial suppliers (Alfa Aesar, Ward Hill, MA, USA; Adamas, Shanghai, China) and stored under nitrogen atmosphere. Unless otherwise noted, materials were obtained from commercial suppliers and used without further purification. Some of the *α*-chloroketones and bromoketones were prepared using literature methods [28,29].

### 3.2. General Procedure for the Reaction between Phenol and α-Haloketone

To a 25 mL two-necked flask equipped with a reflux condenser, fresh distilled 2,2,2-trifluoroethanol (1.0 mL), phenol (1.0 mmol), and titanium tetrachloride (1.0 mmol) were added under nitrogen atmosphere. Then, a mixture of α-haloketone (1.2 mmol) in 2,2,2-trifluoroethanol (1.0 mL) was dropped into the reaction mixture under refluxing temperature. After completion of the reaction (monitored by TLC), the mixture was quenched with a saturated aqueous solution of NH_4_Cl (20 mL). After filtration of the mixture, the water layer was extracted by dichloromethane (3 × 10 mL) and dried with anhydrous sodium sulphate. The organic mixture was concentrated under reduced pressure, and separated by silica-gel column chromatography using ethyl acetate−hexane as eluent in increasing polarity to yield the desired furan compound.

#### 3.2.1. Characterizations of Naphthofuran 6 (Table 2)

*2-Methylnaphtho[2,1-b]furan* (**6a**) [30]. The title compound was obtained as white solid (76%), mp: 39–40 °C, and the analytical data are consistent with those in the literature. ^1^H NMR (600 MHz, CDCl_3_) δ 8.07 (d, *J* = 8.2 Hz, 1H), 7.93 (d, *J* = 8.1 Hz, 1H), 7.65 (d, *J* = 8.8 Hz, 1H), 7.60 (d, *J* = 8.9 Hz, 1H), 7.55 (t, *J* = 7.5 Hz, 1H), 7.46 (t, *J* = 7.5 Hz, 1H), 6.87 (s, 1H), 2.56 (s, 3H); ^13^C NMR (150 MHz, CDCl_3_) δ 154.7, 151.9, 130.3, 128.7, 127.4, 125.9, 124.2, 124.2, 123.8, 123.4, 112.1, 101.7, 14.3; GC-MS (*m/z*): 182.1 [M]^+^.

*2-Methylnaphtho[1–b]furan* (**6b**) [31]. The title compound was obtained as colorless oil (80%), and the analytical data are consistent with those in the literature. ^1^H NMR (600 MHz, CDCl_3_) δ 8.27 (d, *J* = 8.2 Hz, 1H), 7.91 (d, *J* = 8.2 Hz, 1H), 7.74–7.51 (m, 3H), 7.45 (d, *J* = 7.6 Hz, 1H), 6.51 (s, 1H), 2.58 (s, 3H); ^13^C NMR (150 MHz, CDCl_3_) δ 154.6, 149.9, 130.8, 128.3, 126.1, 124.6, 124.4, 123.0, 121.2, 119.7, 119.3, 103.7, 14.2.

*7-Bromo-2-methylnaphtho[2,1-b]furan* (**6c**) [32]. The title compound was obtained as white solid (89%), mp: 91–92 °C, and the analytical data are consistent with those in the literature. ^1^H NMR (600 MHz, CDCl_3_) δ 8.06 (d, *J* = 1.8 Hz, 1H), 7.90 (d, *J* = 8.7 Hz, 1H), 7.60 (dd, *J* = 8.8, 1.8 Hz, 2H), 7.53 (d, *J* = 8.9 Hz, 1H), 6.81 (s, 1H), 2.55 (s, 3H); ^13^C NMR (150 MHz, CDCl_3_) δ 155.3, 151.9, 131.4, 130.6, 129.0, 125.8, 125.2, 124.3, 122.7, 117.8, 113.1, 101.6, 14.2.

*8-Bromo-2-methylnaphtho[2,1-b]furan* (**6d**). The title compound was obtained as white solid (66%), mp: 93–94 °C. ^1^H NMR (600 MHz, CDCl_3_) δ 8.20 (d, *J* = 1.9 Hz, 1H), 7.77 (d, *J* = 8.7 Hz, 1H), 7.59 (s, 2H), 7.52 (dd, *J* = 8.7, 2.0 Hz, 1H), 6.80 (s, 1H), 2.55 (d, *J* = 0.8 Hz, 3H); ^13^C NMR (150 MHz, CDCl_3_) δ 155.2, 152.3, 130.3, 128.6, 128.5, 127.5, 125.9, 123.6, 123.5, 120.0, 112.5, 101.6, 14.2; HRMS (ESI) calcd for C_13_H_10_BrO (M + H)^+^: 260.9910. Found: 260.9909.

*1,2-Dimethylnaphtho[2,1-b]furan* (**6e**) [33]. The title compound was obtained as yellowish oil (74%), and the analytical data are consistent with those in the literature. ^1^H NMR (600 MHz, CDCl_3_) δ 8.40 (d, *J* = 8.3 Hz, 1H), 7.96 (d, *J* = 8.1 Hz, 1H), 7.65 (d, *J* = 8.8 Hz, 1H), 7.63–7.53 (m, 2H), 7.53–7.38 (m, 1H), 2.57 (s, 3H), 2.49 (s, 3H); ^13^C NMR (150 MHz, CDCl_3_) δ 151.3, 149.9, 130.7, 128.9, 128.7, 125.8, 124.0, 123.8, 123.2, 123.0, 112.1, 111.7, 11.8, 11.4.

*2,3-Dimethylnaphtho[1,2-b]furan* (**6f**) [30]. The title compound was obtained as yellowish solid (71%), mp: 201–202 °C, and the analytical data are consistent with those in the literature. ^1^H NMR (400 MHz, CDCl_3_) δ 8.26 (d, *J* = 8.3 Hz, 1H), 7.92 (d, *J* = 8.2 Hz, 1H), 7.64 (d, *J* = 8.5 Hz, 1H), 7.61–7.51 (m, 2H), 7.51–7.36 (m, 1H), 2.50 (s, 3H), 2.24 (s, 3H); ^13^C NMR (100 MHz, CDCl_3_) δ 149.8, 148.9, 130.9, 128.3, 126.0, 125.7, 124.3, 122.5, 121.1, 119.7, 117.9, 110.9, 12.00, 8.1.

*2-Ethyl-3-methylnaphtho[1,2-b]furan* (**6g**). The title compound was obtained as yellowish oil (88%). ^1^H NMR (600 MHz, CDCl_3_) δ 8.27 (d, *J* = 8.2 Hz, 1H), 7.91 (d, *J* = 8.2 Hz, 1H), 7.63 (d, *J* = 8.4 Hz, 1H), 7.54 (t, *J* = 7.5 Hz, 2H), 7.43 (t, *J* = 7.5 Hz, 1H), 2.86 (q, *J* = 7.6 Hz, 2H), 2.25 (s, 3H), 1.36 (t, *J* = 7.6 Hz, 3H); ^13^C NMR (150 MHz, CDCl_3_) δ 154.9, 148.9, 130.9, 128.3, 126.0, 125.7, 124.3, 122.5, 121.2, 119.8, 118.0, 109.9, 19.9, 13.1, 8.0; HRMS (ESI) calcd for C_15_H_15_O (M + H)^+^: 211.1117. Found: 211.1118.

*2-Ethyl-1-methylnaphtho[2,1-b]furan* (**6h**) [26]. The title compound was obtained as yellowish oil (80%), and the analytical data are consistent with those in the literature. ^1^H NMR (400 MHz, CDCl_3_) δ 8.41 (d, *J* = 8.3 Hz, 1H), 7.96 (d, *J* = 8.1 Hz, 1H), 7.74–7.53 (m, 3H), 7.53–7.41 (m, 1H), 2.87 (q, *J* = 7.6 Hz, 2H), 2.59 (s, 3H), 1.36 (t, *J* = 7.6 Hz, 3H); ^13^C NMR (100 MHz, CDCl_3_) δ 155.0, 151.3, 130.7, 129.0, 128.9, 125.8, 124.0, 123.7, 123.3, 123.1, 112.2, 110.8, 19.6, 13.2, 11.3.

*2-Benzyl-1-phenylnaphtho[2,1-b]furan* (**6i**) [34]. The title compound was obtained as yellow oil (70%), and the analytical data are consistent with those in the literature. ^1^H NMR (400 MHz, CDCl_3_) δ 7.90 (d, *J* = 8.2 Hz, 1H), 7.71 (dd, *J* = 12.8, 8.7 Hz, 2H), 7.63 (d, *J* = 8.9 Hz, 1H), 7.58–7.44 (m, 5H), 7.38 (t, *J* = 7.5 Hz, 1H), 7.35–7.20 (m, 6H), 4.08 (s, 2H); ^13^C NMR (100 MHz, CDCl_3_) δ 152.7, 151.7, 138.2, 133.9, 130.7, 130.6, 128.8, 128.7, 128.6, 128.6, 127.8, 126.5, 125.8, 125.0, 124.1, 123.2, 122.2, 120.0, 112.3, 32.6.

*2-Benzyl-3-phenylnaphtho[1,2-b]furan* (**6j**) [35]. The title compound was obtained as yellow oil (74%), and the analytical data are consistent with those in the literature. ^1^H NMR (400 MHz, CDCl_3_) δ 8.29 (d, *J* = 8.2 Hz, 1H), 7.92 (d, *J* = 8.1 Hz, 1H), 7.67 (s, 2H), 7.56 (ddd, *J* = 8.1, 5.1, 2.1 Hz, 3H), 7.53–7.45 (m, 3H), 7.45–7.19 (m, 6H), 4.32 (s, 2H); LCMS (ESI) calcd for C_25_H_19_O (M + H)^+^: 335.1. Found: 335.0.

*2-Butyl-1-propylnaphtho[2,1-b]furan* (*6k*). The title compound was obtained as yellowish oil (83%). ^1^H NMR (400 MHz, CDCl_3_) δ 8.24 (d, *J* = 8.1 Hz, 1H), 7.92 (d, *J* = 8.2 Hz, 1H), 7.63 (d, *J* = 8.9 Hz, 1H), 7.62–7.50 (m, 2H), 7.44 (dd, *J* = 11.1, 4.0 Hz, 1H), 3.03–2.87 (m, 2H), 2.88–2.69 (m, 2H), 1.87–1.67 (m, 4H), 1.42 (dd, *J* = 15.0, 7.5 Hz, 2H), 1.06 (t, *J* = 7.4 Hz, 3H), 0.96 (t, *J* = 7.4 Hz, 3H); ^13^C NMR (100 MHz, CDCl_3_) δ 154.4, 151.6, 130.7, 129.1, 128.4, 125.9, 124.1, 123.7, 123.2, 122.5, 116.2, 112.3, 30.9, 27.3, 26.0, 23.4, 22.5, 14.1, 14.0; HRMS (ESI) calcd for C_19_H_23_O (M + H)^+^: 267.1743. Found: 267.1741.

*2-Butyl-3-propylnaphtho[1,2-b]furan* (**6l**). The title compound was obtained as yellowish oil (84%). ^1^H NMR (400 MHz, CDCl_3_) δ 8.26 (d, *J* = 8.3 Hz, 1H), 7.90 (d, *J* = 8.2 Hz, 1H), 7.57 (ddd, *J* = 17.6, 16.0, 8.2 Hz, 3H), 7.50–7.36 (m, 1H), 2.82 (dt, *J* = 9.9, 7.5 Hz, 2H), 2.68 (dd, *J* = 15.8, 7.9 Hz, 2H), 1.91–1.72 (m, 2H), 1.67 (dd, *J* = 15.7, 7.6 Hz, 2H), 1.51–1.33 (m, 2H), 0.98 (ddt, *J* = 16.3, 8.9, 7.4 Hz, 6H); ^13^C NMR (100 MHz, CDCl_3_) δ 154.1, 149.1, 130.8, 128.3, 125.9, 125.0, 124.3, 122.3, 121.2, 119.8, 118.3, 115.8, 23.5, 23.4, 22.6, 22.5, 22.1, 14.1, 13.9; HRMS (ESI) calcd for C_19_H_23_O (M + H)^+^: 267.1743. Found: 267.1744.

*2-Ethylnaphtho[1,2-b]furan* (**6m**) [36].The title compound was obtained as yellowish oil (40%). ^1^H NMR (600 MHz, CDCl_3_) δ 8.27 (d, *J* = 8.3 Hz, 1H), 7.91 (d, *J* = 8.2 Hz, 1H), 7.65–7.51 (m, 3H), 7.50–7.38 (m, 1H), 6.51 (d, *J* = 0.9 Hz, 1H), 2.92 (qd, *J* = 7.5, 0.9 Hz, 2H), 1.40 (t, *J* = 7.6 Hz, 3H). ^13^C NMR (150 MHz, CDCl_3_) δ 160.3, 149.8, 130.9, 128.3, 126.1, 124.4, 124.4, 122.9, 121.2, 119.7, 119.4, 102.1, 21.9, 12.2.

#### 3.2.2. Characterizations of Naphthofuran7 (Table 3)

*9,10-Dihydro-8H-cyclopenta[b]naphtho[1,2-d]furan* (**7a**). The title compound was obtained as white solid (72%), mp: 125–126 °C. ^1^H NMR (400 MHz, CDCl_3_) δ 8.05 (d, *J* = 8.1 Hz, 1H), 7.90 (d, *J* = 8.1 Hz, 1H), 7.59 (s, 2H), 7.51 (t, *J* = 7.4 Hz, 1H), 7.43 (t, *J* = 7.5 Hz, 1H), 3.16–2.99 (m, 2H), 2.91 (t, *J* = 7.2 Hz, 2H), 2.73–2.52 (m, 2H); ^13^C NMR (100 MHz,, CDCl_3_) δ 162.0, 157.3, 130.4, 128.5, 127.5, 125.8, 124.2, 124.1, 123.1, 122.2, 121.7, 113.0, 27.9, 25.0, 23.9; HRMS (ESI) calcd for C_15_H_13_O (M + H)^+^: 209.0961. Found: 209.0960.

*8,9,10,11-Tetrahydronaphtho[2,1-b]benzofuran* (**7b**) [37]. The title compound was obtained as yellowish oil (86%), and the analytical data are consistent with those in the literature. ^1^H NMR (400 MHz, CDCl_3_) δ 8.25 (d, *J* = 8.2 Hz, 1H), 7.94 (d, *J* = 8.1 Hz, 1H), 7.75–7.60 (m, 2H), 7.60–7.50 (m, 1H), 7.50–7.37 (m, 1H), 3.21–3.03 (m, 2H), 2.85 (t, *J* = 4.8 Hz, 2H), 2.14–1.86 (m, 4H); ^13^C NMR (150 MHz, CDCl_3_) δ 153.3, 151.5, 130.6, 129.5, 128.7, 125.7, 123.8, 123.7, 123.5, 122.5, 114.3, 112.3, 23.8, 23.2, 23.1, 22.6.

*9,10,11,12-Tetrahydro-8H-cyclohepta[b]naphtho[1,2-d]furan* (**7c**). The title compound was obtained as yellowish oil (78%). ^1^H NMR (400 MHz, CDCl_3_) δ 8.46 (d, *J* = 8.4 Hz, 1H), 7.94 (d, *J* = 8.1 Hz, 1H), 7.63 (d, *J* = 8.8 Hz, 1H), 7.61–7.52 (m, 2H), 7.45 (t, *J* = 7.5 Hz, 1H), 3.37–3.17 (m, 2H), 3.03 (d, *J* = 5.9 Hz, 2H), 2.13–1.77 (m, 6H); ^13^C NMR (100 MHz, CDCl_3_) δ 155.8, 150.7, 130.8, 129.1, 128.8, 125.6, 124.1, 123.7, 123.1, 123.0, 118.3, 112.2, 29.6, 28.5, 28.0, 26.1, 25.8;HRMS (ESI) calcd for C_17_H_17_O (M + H)^+^: 237.1274. Found: 237.1276.

*8,9,10,11,12,13-Hexahydrocycloocta[b]naphtho[1,2-d]furan* (**7d**). The title compound was obtained as white solid (71%).^1^H NMR (400 MHz, CDCl_3_) δ 8.37 (d, *J* = 8.3 Hz, 1H), 7.95 (d, *J* = 8.1 Hz, 1H), 7.62 (q, *J* = 8.8 Hz, 2H), 7.58–7.52 (m, 1H), 7.50–7.42 (m, 1H), 3.30–3.12 (m, 2H), 3.12–2.88 (m, 2H), 1.96 (dt, *J* = 12.6, 6.3 Hz, 2H), 1.89–1.73 (m, 2H), 1.68–1.53 (m, 2H), 1.46 (dd, *J* = 11.2, 5.5 Hz, 2H);^13^C NMR (100 MHz, CDCl_3_) δ 155.0, 151.3, 130.6, 129.0, 128.6, 125.8, 123.8, 123.8, 123.0, 122.6, 115.6, 112.4, 29.6, 28.1, 26.7, 26.0, 25.8, 23.3;HRMS (ESI) calcd for C_18_H_19_O (M + H)^+^: 251.1430. Found: 251.1435.

*7,8,9,10-Tetrahydronaphtho[1,2-b]benzofuran* (**7e**) [36].The title compound was obtained as yellowish oil (92%), and the analytical data are consistent with those in the literature. ^1^H NMR (400 MHz, CDCl_3_) δ 8.29 (d, *J* = 8.3 Hz, 1H), 7.93 (d, *J* = 8.2 Hz, 1H), 7.65 (d, *J* = 8.4 Hz, 1H), 7.61–7.50 (m, 2H), 7.51–7.37 (m, 1H), 2.99–2.81 (m, 2H), 2.81–2.61 (m, 2H), 2.14–1.96 (m, 2H), 1.96–1.80 (m, 2H); LCMS (ESI) calcd for C_16_H_15_O (M + H)^+^: 223.1. Found: 223.3.

*8,9,10,11-Tetrahydro-7H-cyclohepta[b]naphtho[2,1-d]furan* (**7f**) [38]. The title compound was obtained as white solid (84%), mp: 38–39 °C, and the analytical data are consistent with those in the literature. ^1^H NMR (400 MHz, CDCl_3_)δ 8.25 (d, *J* = 8.3 Hz, 1H), 7.91 (d, *J* = 8.2 Hz, 1H), 7.63 (d, *J* = 8.5 Hz, 1H), 7.54 (ddd, *J* = 8.5, 5.5, 2.0 Hz, 2H), 7.43 (ddd, *J* = 8.1, 6.9, 1.2 Hz, 1H), 3.14–2.97 (m, 2H), 2.89–2.71 (m, 2H), 2.00–1.75 (m, 6H); ^13^C NMR (100 MHz, CDCl3) δ 155.8, 148.2, 130.8, 128.3, 126.0, 125.7, 124.2, 122.5, 121.22, 119.8, 117.7, 117.2, 30.8, 29.4, 28.4, 26.5, 23.5.

*7,8,9,10,11,12-Hexahydrocycloocta[b]naphtho[2,1-d]furan* (**7g**) [38]. The title compound was obtained as colorless oil (87%), and the analytical data are consistent with those in the literature. ^1^H NMR (400 MHz, CDCl_3_) δ 8.29 (d, *J* = 8.3 Hz, 1H), 7.93 (d, *J* = 8.2 Hz, 1H), 7.65 (d, *J* = 8.5 Hz, 1H), 7.57 (t, *J* = 8.1 Hz, 2H), 7.45 (t, *J* = 7.4 Hz, 1H), 3.18–2.99 (m, 2H), 2.99–2.82 (m, 2H), 2.03–1.72 (m, 4H), 1.68–1.44 (m, 4H); ^13^C NMR (100 MHz, CDCl_3_) δ 154.3, 148.8, 130.8, 128.4, 126.0, 125.2, 124.3, 122.5, 121.3, 119.8, 117.7, 115.0, 28.3, 27.6, 26.5, 26.1, 25.6, 21.6.

*3-Bromo-9,10-dihydro-8H-cyclopenta[b]naphtho[1,2-d]furan* (**7h**). The title compound was obtained as white solid (61%), mp: 36–37 °C. ^1^H NMR (600 MHz, CDCl_3_) δ 8.06 (s, 1H), 7.93 (d, *J* = 8.7 Hz, 1H), 7.60 (dd, *J* = 18.5, 8.8 Hz, 2H), 7.51 (d, *J* = 8.9 Hz, 1H), 3.06 (t, *J* = 6.8 Hz, 2H), 3.00–2.89 (m, 2H), 2.77–2.56 (m, 2H); ^13^C NMR (150 MHz, CDCl_3_) δ 162.7, 157.3, 131.5, 130.4, 128.9, 125.9, 125.8, 122.1, 122.0, 121.8, 117.9, 114.0, 27.9, 25.0, 23.8; HRMS (ESI) calcd for C_15_H_12_BrO (M + H)^+^: 287.0066. Found: 287.0068.

*3-Bromo-8,9,10,11-tetrahydronaphtho[2,1-b]benzofuran* (**7i**). The title compound was obtained as white solid (77%). ^1^H NMR (400 MHz, CDCl_3_) δ 8.04 (dd, *J* = 9.2, 5.4 Hz, 2H), 7.68–7.53 (m, 2H), 7.49 (d, *J* = 8.9 Hz, 1H), 3.00 (t, *J* = 4.8 Hz, 2H), 2.83 (t, *J* = 5.0 Hz, 2H), 2.04–1.84 (m, 4H); ^13^C NMR (100 MHz, CDCl_3_) δ 153.9, 151.5, 131.8, 130.7, 128.8, 126.8, 125.2, 122.7, 122.6, 117.5, 114.1, 113.3, 23.7, 23.0, 23.0, 22.5; HRMS (ESI) calcd for C_16_H_14_BrO (M + H)^+^: 301.0223. Found: 301.0220.

*3-Bromo-9,10,11,12-tetrahydro-8H-cyclohepta[b]naphtho[1,2-d]furan* (**7j**). The title compound was obtained as yellowish oil (65%). ^1^H NMR (400 MHz, CDCl_3_) δ 8.29 (d, *J* = 9.0 Hz, 1H), 8.06 (d, *J* = 2.0 Hz, 1H), 7.56 (ddd, *J* = 25.1, 13.7, 5.5 Hz, 3H), 3.34–3.09 (m, 2H), 3.01 (d, *J* = 6.1 Hz, 2H), 2.06–1.76 (m, 6H); ^13^C NMR (100 MHz, CDCl_3_) δ 156.4, 150.7, 132.1, 131.0, 128.7, 127.2, 124.8, 123.2, 123.0, 118.1, 117.2, 113.2, 29.5, 28.5, 27.9, 26.0, 25.7; HRMS (ESI) calcd for C_17_H_16_BrO (M + H)^+^: 315.0379. Found: 315.0381.

*3-Bromo-8,9,10,11,12,13-hexahydrocycloocta[b]naphtho[1,2-d]furan* (**7k**). The title compound was obtained as white solid (68%), mp: 36–37 °C. ^1^H NMR (600 MHz, CDCl_3_) δ 8.20 (s, 1H), 8.07 (s, 1H), 7.60 (s, 2H), 7.52 (s, 1H), 3.13 (s, 2H), 2.98 (s, 2H), 1.93 (s, 2H), 1.78 (s, 2H), 1.58 (s, 2H), 1.45 (s, 2H); ^13^C NMR (150 MHz, CDCl_3_) δ 155.5, 151.4, 132.0, 130.9, 128.9, 127.0, 124.7, 122.8, 122.7, 117.4, 115.5, 113.4, 29.5, 28.0, 26.6, 26.0, 25.7, 23.3; HRMS (ESI) calcd for C_18_H_18_BrO (M + H)^+^: 329.0536. Found: 329.0534.

*2-Bromo-9,10-dihydro-8H-cyclopenta[b]naphtho[1,2-d]furan* (**7l**). The title compound was obtained as white solid (62%), mp: 35–36 °C ^1^H NMR (600 MHz, CDCl_3_) δ 8.18 (d, *J* = 1.9 Hz, 1H), 7.78 (d, *J* = 8.7 Hz, 1H), 7.59 (dd, *J* = 22.5, 8.9 Hz, 2H), 7.52 (dd, *J* = 8.7, 2.0 Hz, 1H), 3.15–3.02 (m, 2H), 3.02–2.84 (m, 2H), 2.68 (dd, *J* = 14.2, 7.4 Hz, 2H); HRMS (ESI) calcd for C_15_H_12_BrO (M + H)^+^: 287.0066. Found: 287.0069.

*2-Bromo-8,9,10,11-tetrahydronaphtho[2,1-b]benzofuran* (**7m**). The title compound was obtained as white solid (81%). ^1^H NMR (400 MHz, CDCl_3_) δ 8.31 (d, *J* = 1.8 Hz, 1H), 7.76 (d, *J* = 8.7 Hz, 1H), 7.64–7.53 (m, 2H), 7.50 (dd, *J* = 8.7, 1.9 Hz, 1H), 3.04 (t, *J* = 4.7 Hz, 2H), 2.83 (t, *J* = 4.9 Hz, 2H), 1.96 (dd, *J* = 6.7, 3.9 Hz, 4H); ^13^C NMR (100 MHz, CDCl_3_) δ 153.8, 151.9, 130.3, 129.5, 128.9, 127.1, 125.8, 123.4, 121.8, 119.8, 114.1, 112.7, 23.7, 22.97, 22.92, 22.5; HRMS (ESI) calcd for C_16_H_14_BrO (M + H)^+^: 301.0223. Found: 301.0220.

*2-Bromo-9,10,11,12-tetrahydro-8H-cyclohepta[b]naphtho[1,2-d]furan* (**7n**). The title compound was obtained as yellowish oil (68%). ^1^H NMR (400 MHz, CDCl_3_) δ 8.54 (d, *J* = 1.7 Hz, 1H), 7.78 (d, *J* = 8.7 Hz, 1H), 7.66–7.45 (m, 3H), 3.31–3.10 (m, 2H), 3.01 (d, *J* = 6.2 Hz, 2H), 2.10–1.73 (m, 6H); ^13^C NMR (100 MHz, CDCl_3_) δ 156.3, 151.1, 130.6, 129.8, 129.2, 126.9, 125.5, 123.8, 122.4, 119.9, 118.1, 112.6, 29.5, 28.5, 27.9, 25.97, 25.58; HRMS (ESI) calcd for C_17_H_16_BrO (M + H)^+^: 315.0379. Found: 315.0377.

*2-Bromo-8,9,10,11,12,13-hexahydrocycloocta[b]naphtho[1,2-d]furan* (**7o**). The title compound was obtained as colorless oil (70%). ^1^H NMR (600 MHz, CDCl_3_) δ 8.45 (d, *J* = 1.8 Hz, 1H), 7.78 (d, *J* = 8.7 Hz, 1H), 7.58 (d, *J* = 1.9 Hz, 2H), 7.51 (dd, *J* = 8.7, 1.9 Hz, 1H), 3.22–3.09 (m, 2H), 3.09–2.86 (m, 2H), 2.07–1.90 (m, 2H), 1.88–1.73 (m, 2H), 1.58 (d, *J* = 6.3 Hz, 2H), 1.52–1.40 (m, 2H);^13^C NMR (150 MHz, CDCl_3_) δ 155.3, 151.7, 130.5, 129.7, 129.1, 127.1, 125.4, 123.6, 121.9, 120.0, 115.5, 112.8, 29.4, 27.9, 26.7, 26.0, 25.6, 23.2; HRMS (ESI) calcd for C_18_H_18_BrO (M + H)^+^: 329.0536. Found: 329.0538.

#### 3.2.3. Characterizations of Benzofuran 9 (Table 4)

*1,2,3,4-Tetrahydrodibenzo[b,d]furan* (**9a**) [39]. The title compound was obtained as colorless oil (68%), and the analytical data are consistent with those in the literature. ^1^H NMR (600 MHz, CDCl_3_) δ 7.42 (ddd, *J* = 5.7, 3.3, 1.1 Hz, 2H), 7.21 (pd, *J* = 7.2, 3.8 Hz, 2H), 2.76 (tt, *J* = 6.3, 1.8 Hz, 2H), 2.64 (tt, *J* = 6.0, 1.9 Hz, 2H), 2.04–1.92 (m, 2H), 1.92–1.79 (m, 2H); ^13^C NMR (150 MHz, CDCl_3_) δ 154.3, 154.0, 128.9, 123.0, 122.1, 118.4, 112.9, 110.8, 23.46, 23.0, 22.7, 20.5.

*6-Methyl-1,2,3,4-tetrahydrodibenzo[b,d]furan* (**9b**). The title compound was obtained as colorless oil (76%). ^1^H NMR (600 MHz, CDCl_3_) δ 7.25 (d, *J* = 7.6 Hz, 1H), 7.10 (t, *J* = 7.5 Hz, 1H), 7.01 (d, *J* = 7.3 Hz, 1H), 2.76 (ddd, *J* = 8.1, 4.1, 1.9 Hz, 2H), 2.62 (tt, *J* = 5.9, 1.8 Hz, 2H), 2.51 (s, 3H), 2.02–1.91 (m, 2H), 1.91–1.78 (m, 2H); ^13^C NMR (150 MHz, CDCl_3_) δ 152.6, 152.2, 127.3, 123.0, 121.1, 119.9, 114.8, 112.0, 22.5, 22.0, 21.7, 19.5, 14.0; HRMS (ESI) calcd for C_13_H_15_O (M + H)^+^: 187.1117. Found: 187.1121.

*7-Methyl-1,2,3,4-tetrahydrodibenzo[b,d]furan* (**9c**) [40]. The title compound was obtained as yellowish oil (82%), and the analytical data are consistent with those in the literature. ^1^H NMR (600 MHz, CDCl_3_) δ 7.28 (d, *J* = 7.8 Hz, 1H), 7.21 (d, *J* = 0.5 Hz, 1H), 7.01 (d, *J* = 7.8 Hz, 1H), 2.81–2.68 (m, 2H), 2.61 (tt, *J* = 5.9, 1.8 Hz, 2H), 2.46 (s, 3H), 2.01–1.90 (m, 2H), 1.90–1.77 (m, 2H); ^13^C NMR (150 MHz, CDCl_3_) δ 153.7, 152.3, 131.9, 125.3, 122.3, 116.8, 111.6, 110.1, 22.4, 21.9, 21.7, 20.6, 19.5.

*8-Methyl-1,2,3,4-tetrahydrodibenzo[b,d]furan* (**9d**) [40]. The title compound was obtained as colorless oil (72%), and the analytical data are consistent with those in the literature. ^1^H NMR (600 MHz, CDCl_3_) δ 7.30 (d, *J* = 8.3 Hz, 1H), 7.22 (s, 1H), 7.03 (dd, *J* = 8.2, 1.2 Hz, 1H), 2.75 (ddd, *J* = 8.0, 4.1, 1.8 Hz, 2H), 2.62 (tt, *J* = 5.9, 1.8 Hz, 2H), 2.46 (s, 3H), 2.03–1.92 (m, 2H), 1.91–1.79 (m, 2H);^13^C NMR (150 MHz, CDCl_3_) δ 154.1, 152.7, 131.5, 129.0, 124.0, 118.4, 112.6, 110.2, 23.5, 23.0, 22.8, 21.4, 20.5.

*6-Methoxy-1,2,3,4-tetrahydrodibenzo[b,d]furan* (**9e**). The title compound was obtained as yellow oil (70%). ^1^H NMR (600 MHz, CDCl_3_) δ 7.11 (t, *J* = 7.8 Hz, 1H), 7.02 (dd, *J* = 7.7, 0.9 Hz, 1H), 6.74 (dd, *J* = 7.9, 0.5 Hz, 1H), 4.00 (s, 3H), 2.84–2.69 (m, 2H), 2.69–2.52 (m, 2H), 1.99–1.89 (m, 2H), 1.89–1.78 (m, 3H); ^13^C NMR (150 MHz, CDCl_3_) δ 154.2, 145.0, 143.2, 130.5, 122.8, 113.2, 111.0, 105.5, 56.0, 23.4, 22.9, 22.7, 20.6; HRMS (ESI) calcd for C_13_H_15_O_2_ (M + H)^+^: 203.1067. Found: 203.1066.

*8-Methoxy-1,2,3,4-tetrahydrodibenzo[b,d]furan* (**9f**) [41]. The title compound was obtained as colorless oil (71%), and the analytical data are consistent with those in the literature. ^1^H NMR (600 MHz, CDCl_3_) δ 7.27 (d, *J* = 8.8 Hz, 1H), 6.87 (d, *J* = 2.6 Hz, 1H), 6.79 (dd, *J* = 8.8, 2.6 Hz, 1H), 3.84 (s, 3H), 2.71 (ddd, *J* = 6.2, 4.8, 1.7 Hz, 2H), 2.59 (tt, *J* = 5.8, 1.7 Hz, 2H), 2.00–1.89 (m, 2H), 1.89–1.77 (m, 2H); ^13^C NMR (150 MHz, CDCl_3_) δ 154.6, 154.0, 148.2, 128.4, 112.0, 110.0, 109.9, 100.5, 54.9, 22.5, 21.9, 21.6, 19.5.

*8-(tert-Butyl)-1,2,3,4-tetrahydrodibenzo[b,d]furan* (**9g**). The title compound was obtained as colorless oil (80%), and the analytical data are consistent with those in the literature. ^1^H NMR (600 MHz, CDCl_3_) δ 7.39 (d, *J* = 1.9 Hz, 1H), 7.31 (d, *J* = 8.6 Hz, 1H), 7.27–7.24 (m, 1H), 2.75–2.69 (m, 2H), 2.63 (ddd, *J* = 7.7, 4.0, 1.8 Hz, 2H), 1.98–1.90 (m, 2H), 1.89–1.78 (m, 2H), 1.37 (s, 9H); ^13^C NMR (150 MHz, CDCl_3_) δ 153.1, 151.4, 144.2, 127.4, 119.7, 113.5, 111.8, 109.0, 33.7, 30.9, 22.5, 22.0, 21.7, 19.5.

*6-Benzyl-1,2,3,4-tetrahydrodibenzo[b,d]furan* (**9h**). The title compound was obtained as yellowish oil (85%). ^1^H NMR (600 MHz, CDCl_3_) δ 7.35–7.27 (m, 5H), 7.24–7.19 (m, 1H), 7.13 (t, *J* = 7.5 Hz, 1H), 6.98 (d, *J* = 7.3 Hz, 1H), 4.26 (s, 2H), 2.88–2.72 (m, 2H), 2.64 (tt, *J* = 5.8, 1.7 Hz, 2H), 2.06–1.92 (m, 2H), 1.87 (dtd, *J* = 9.0, 6.0, 2.8 Hz, 2H); ^13^C NMR (150 MHz, CDCl_3_) δ 153.9, 152.8, 140.5, 129.0, 128.7, 128.39, 126.0, 124.2, 123.6, 122.4, 116.4, 113.1, 77.3, 77.1, 76.9, 35.5, 23.6, 23.0, 22.7, 20.6; HRMS (ESI) calcd for C_19_H_19_O (M + H)^+^: 263.1430. Found: 263.1433.

*2,3-Dimethylbenzofuran* (**9i**) [30]. The title compound was obtained as yellow oil (73%), and the analytical data are consistent with those in the literature. ^1^H NMR (600 MHz, CDCl_3_) δ 7.48–7.39 (m, 1H), 7.39–7.32 (m, 1H), 7.23–7.15 (m, 2H), 2.38 (d, *J* = 0.6 Hz, 3H), 2.16 (d, *J* = 0.7 Hz, 3H); ^13^C NMR (150 MHz, CDCl_3_) δ 152.8, 149.4, 129.4, 122.0, 120.9, 117.5, 109.4, 108.7, 10.8, 6.9.

*2,3,5-Trimethylbenzofuran* (**9j**) [30]. The title compound was obtained as colorless oil (81%), and the analytical data are consistent with those in the literature. ^1^H NMR (600 MHz, CDCl_3_) δ 7.23 (d, *J* = 8.3 Hz, 1H), 7.18 (s, 1H), 7.00 (dd, *J* = 8.2, 1.3 Hz, 1H), 2.43 (s, 3H), 2.36 (s, 3H), 2.12 (s, 3H); ^13^C NMR (150 MHz, CDCl_3_) δ 152.2, 150.6, 131.3, 130.5, 124.1, 118.5, 109.9, 109.4, 21.7, 11.8, 7.9.

*5-Methoxy-2,3-dimethylbenzofuran* (**9k**). The title compound was obtained as yellow oil (77%). ^1^H NMR (600 MHz, CDCl_3_) δ 7.25 (d, *J* = 8.8 Hz, 1H), 6.86 (d, *J* = 2.6 Hz, 1H), 6.79 (dd, *J* = 8.8, 2.6 Hz, 1H), 3.85 (s, 3H), 2.36 (s, 3H), 2.12 (s, 3H); ^13^C NMR (150 MHz, CDCl_3_) δ 154.5, 150.4, 147.7, 130.0, 110.0, 109.7, 108.8, 100.6, 54.9, 10.9, 7.0; HRMS (ESI) calcd for C_11_H_13_O_2_ (M + H)^+^: 177.0910. Found: 177.0909.

## 4. Conclusions

In conclusion, we have found that titanium tetrachloride can act as an efficient Lewis acid catalyst and a strong dehydrating agent to promote the regioselective Friedel–Crafts-like alkylation and subsequent intramolecular cyclodehydration. This process provides a general method for the preparation of a wide range of naphthofurans and benzofurans from readily available phenols and α-haloketones.

## Figures and Tables

**Table 1:**
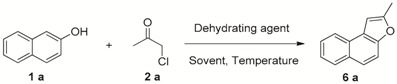
Model reaction optimization ^a^.

Entry	Acid	Solvent	T (°C)	Yield of 6a ^b^
1	HCl	TFE	70	NA
2	TsOH	TFE	70	NA
3	ZnCl_2_	TFE	70	NA
4	AlCl_3_	TFE	70	NA
5	TMSOTf	TFE	70	14
6	BF_3_.Et_2_O	TFE	70	26
7	Al_2_O_3_	xylene	150	-- ^d^
8	TiCl_4_ (1.0 eq)	TFE	70	76%
9	TiCl_4_ (0.2 eq)	TFE	70	16%
10	TiCl_4_ (0.5 eq)	TFE	70	38%
11	TiCl_4_ (1.5 eq)	TFE	70	71%
12	TiCl_4_	TFE	50	45%
13	TiCl_4_	TFE	25	<10%
14	TiCl_4_	CH_2_Cl_2_	40	34%
15	TiCl_4_	Toluene	110	38%
16	TiCl_4_	Solvent ^c^	reflux	NA

^a^ The mole ratio between **1a** and **2a** is 1:1.2. ^b^ Isolated yields. ^c^ Solvents: ACN, Et_2_O, THF. ^d^ An inseparable mixture of **4a** and **6a** was obtained.

**Table 2:** Reaction of naphthols with different acyclic α-haloketones ^a, b, c^.

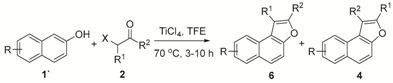
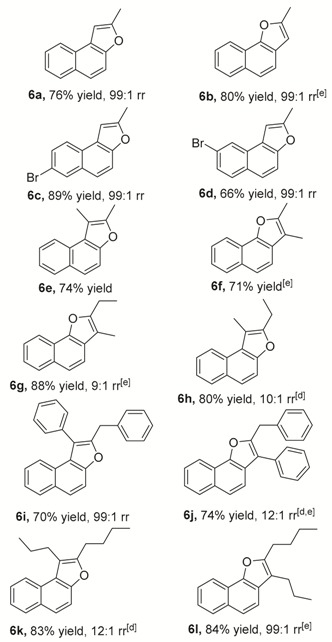

^a^ The molar ratio between naphthol **1**, haloketone **2** and TiCl_4_ is 1:1.2:1. ^b^ Isolated yield. ^c^ The ratio of regioisomers was determined by crude ^1^H NMR (see Supplementary Materials). ^d^ The products were inseparable when purified by chromatography on silica gel. ^e^ Using 1-napthol as starting material.

**Table 3:** Reaction of naphthols with different cyclic α-haloketones ^a, b^.

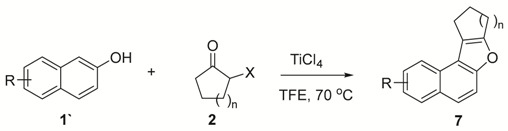
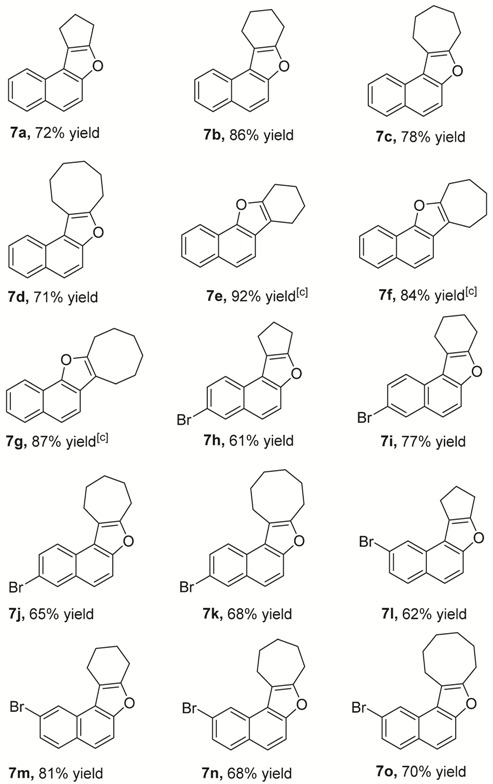

^a^ The molar ratio between naphthol **1** and haloketone **2** is 1:1.2. ^b^ Isolated yield. ^c^ Using 1-napthol as starting material.

**Table 4:** Reaction of phenols with different α-haloketones ^a, b^.

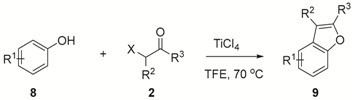
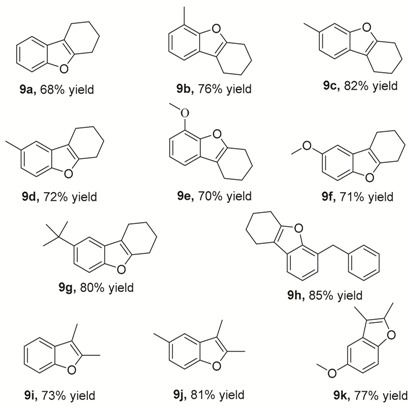

^a^ The molar ratio between phenol **8** and haloketone **2** is 1:1.2. ^b^ Isolated yield.

## Supplementary Materials

The following are available online. The NMR spectra for all the synthesized compounds.
