# Supplementary material for: One-Step Regioselective Synthesis of Benzofurans from Phenols and α-Haloketones
Source: Molecules. 2019 Jun 11;24(11):2187. doi: 10.3390/molecules24112187 (PMC6600630; doi:10.3390/molecules24112187)
Supplement: Supplementary file 1 [file molecules-24-02187-s001.pdf]

Supporting Information

One-step Regioselective Synthesis of Benzofurans from Phenols and α-Haloketones

Bingqiao Wang,[1,&] Qiu Zhang,[1,&] Juan Luo,[1] Zongjie Gan,[1] Wengao Jiang,[1] and Qiang Tang[1,*]

[1] College of Pharmacy, Center for Lab Teaching and Management, Chongqing Medical University, No.1 Yixueyuan Road, Chongqing 400016, P. R. China.
[&] These authors contributed equally to this work and should be considered co-first authors.

* Correspondence: E-mail: tangqiang @cqmu.edu.cn.

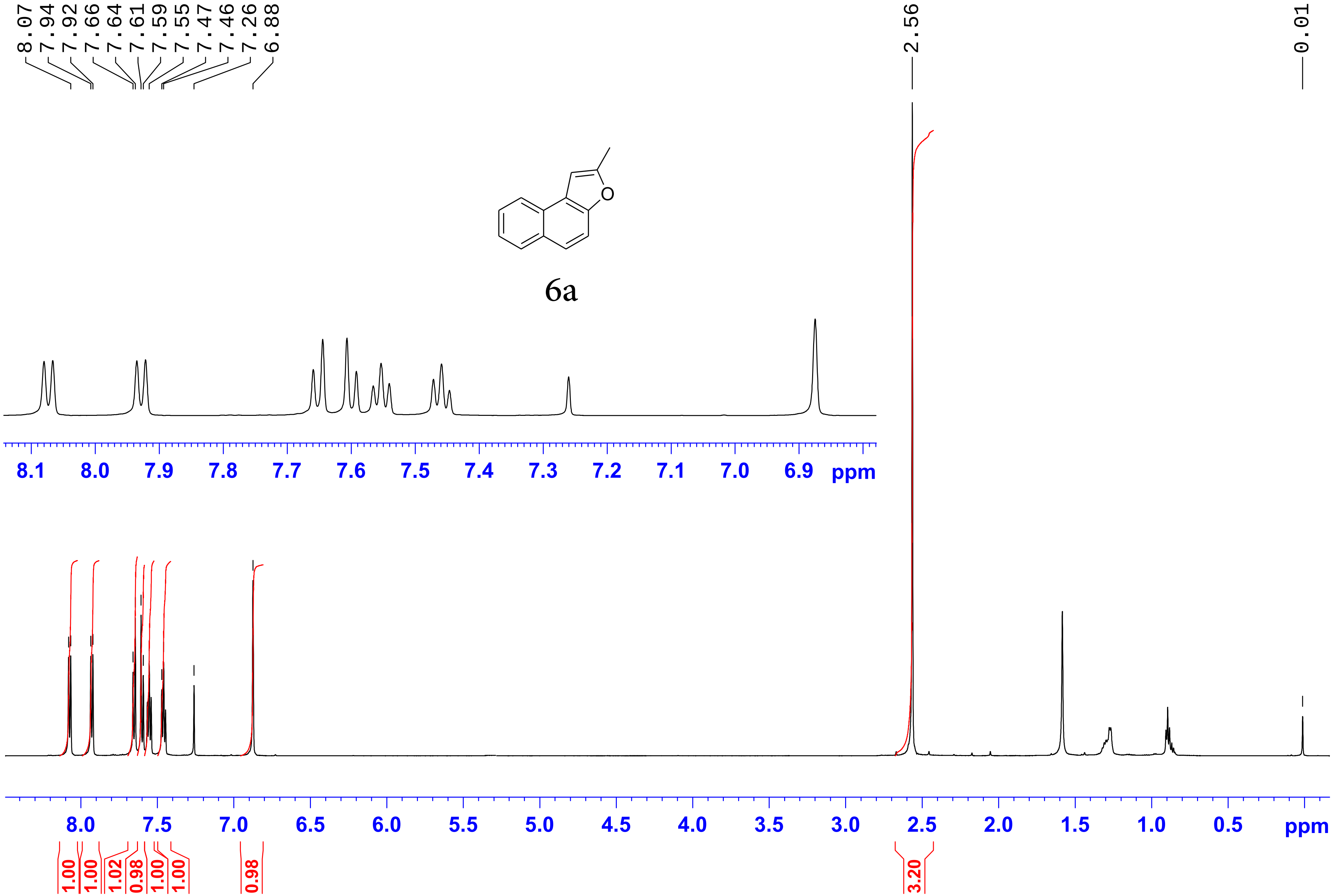

8.07
7.94
7.92
7.66
7.64
7.61
7.59
7.55
7.47
7.46
7.26
6.88
2.56
0.01
6a
8.1 8.0 7.9 7.8 7.7 7.6 7.5 7.4 7.3 7.2 7.1 7.0 6.9 ppm
8.0 7.5 7.0 6.5 6.0 5.5 5.0 4.5 4.0 3.5 3.0 2.5 2.0 1.5 1.0 0.5 ppm
1.00
1.00
1.02
0.98
1.00
1.00
0.98
3.20

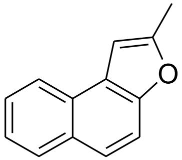

6a

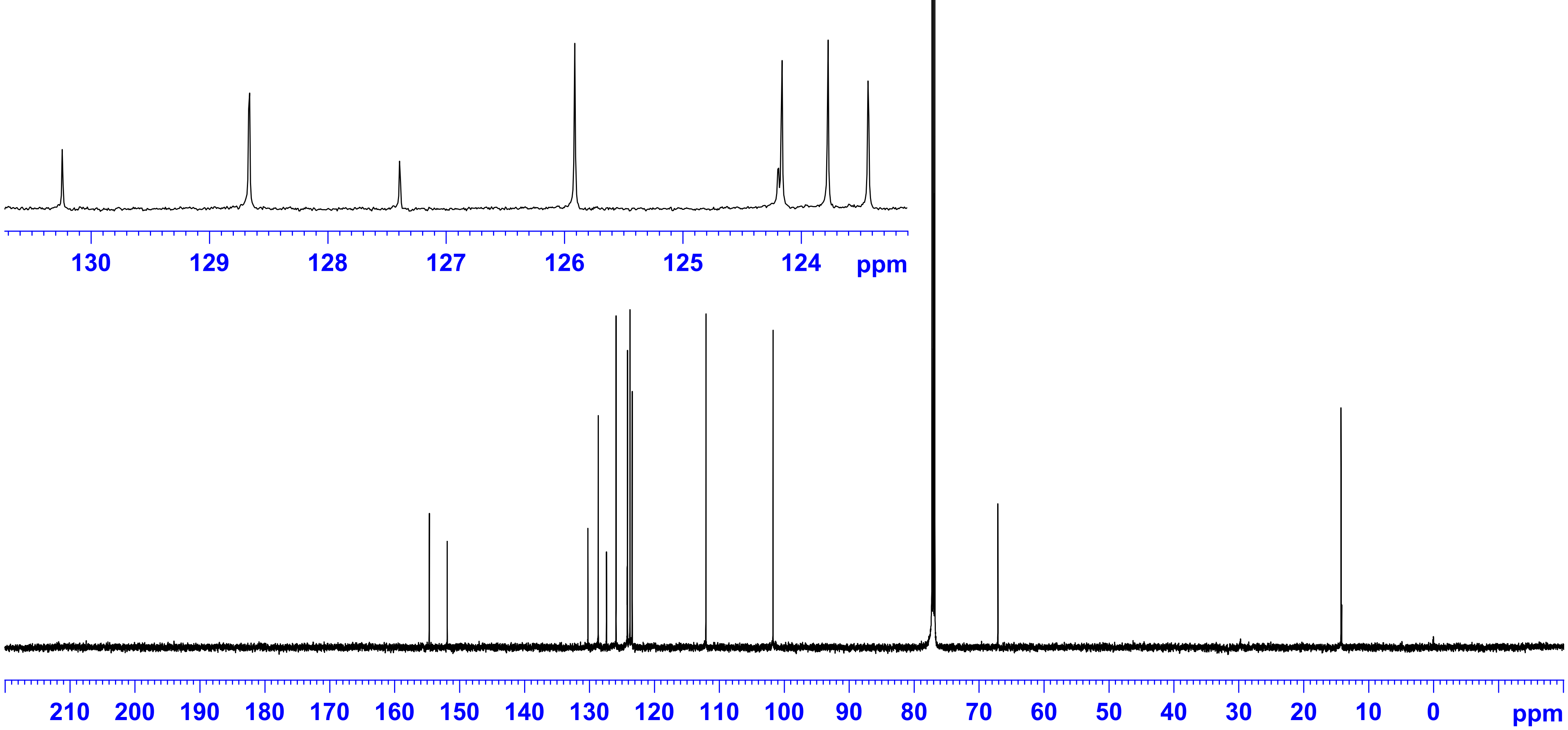
154.69
151.93
130.24
128.66
127.39
125.91
124.20
124.16
123.78
123.44
112.07
101.74
77.25
77.04
76.83
14.25
130
129
128
127
126
125
124
ppm
210
200
190
180
170
160
150
140
130
120
110
100
90
80
70
60
50
40
30
20
10
0
ppm

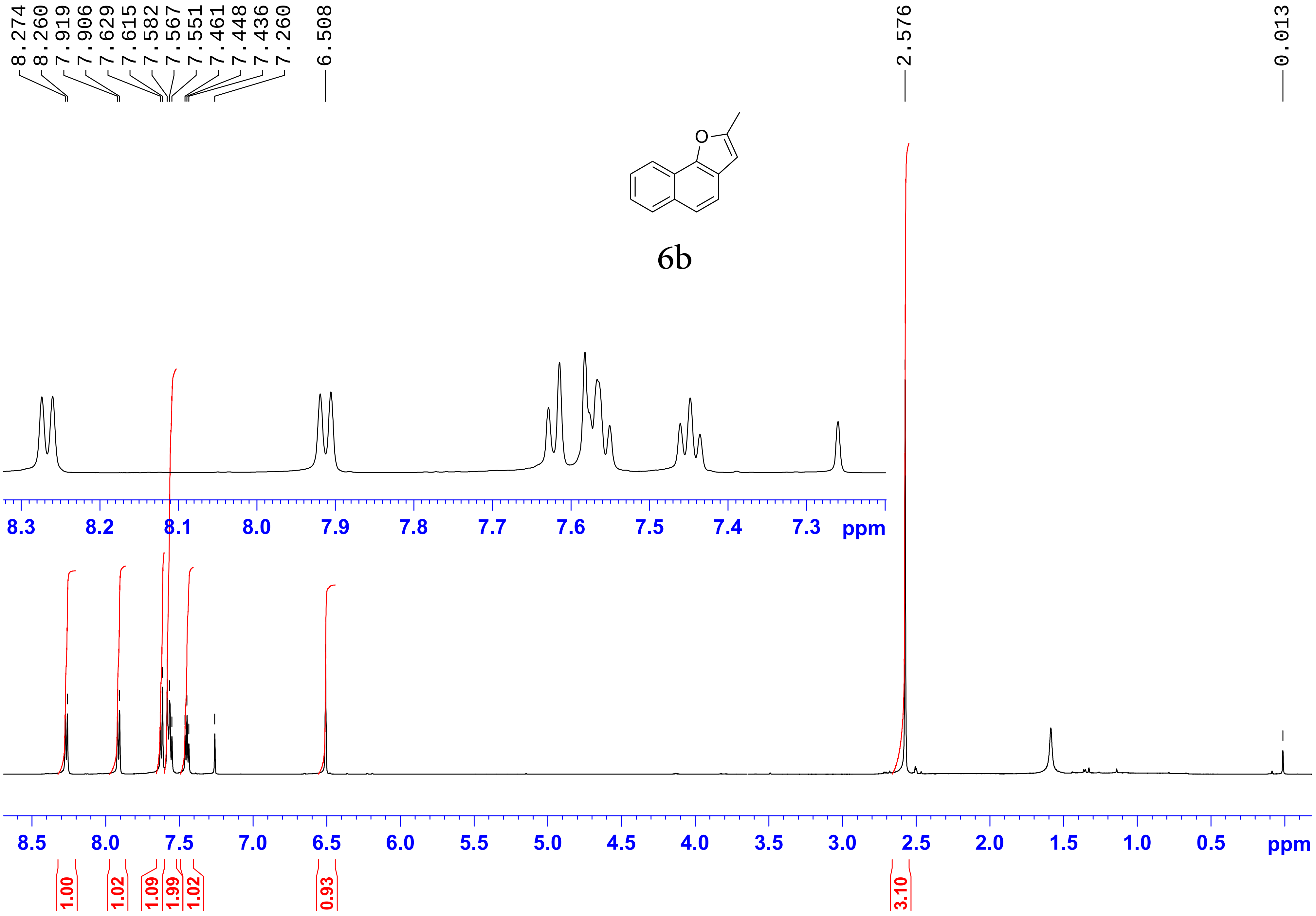
8.274
8.260
7.919
7.906
7.629
7.615
7.582
7.567
7.551
7.461
7.448
7.436
7.260
6.508
2.576
0.013
O
6b
8.3
8.2
8.1
8.0
7.9
7.8
7.7
7.6
7.5
7.4
7.3
ppm
8.5
8.0
7.5
7.0
6.5
6.0
5.5
5.0
4.5
4.0
3.5
3.0
2.5
2.0
1.5
1.0
0.5
ppm
1.00
1.02
1.09
1.99
1.02
0.93
3.10

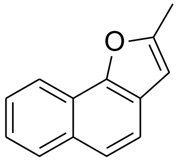
O

6b

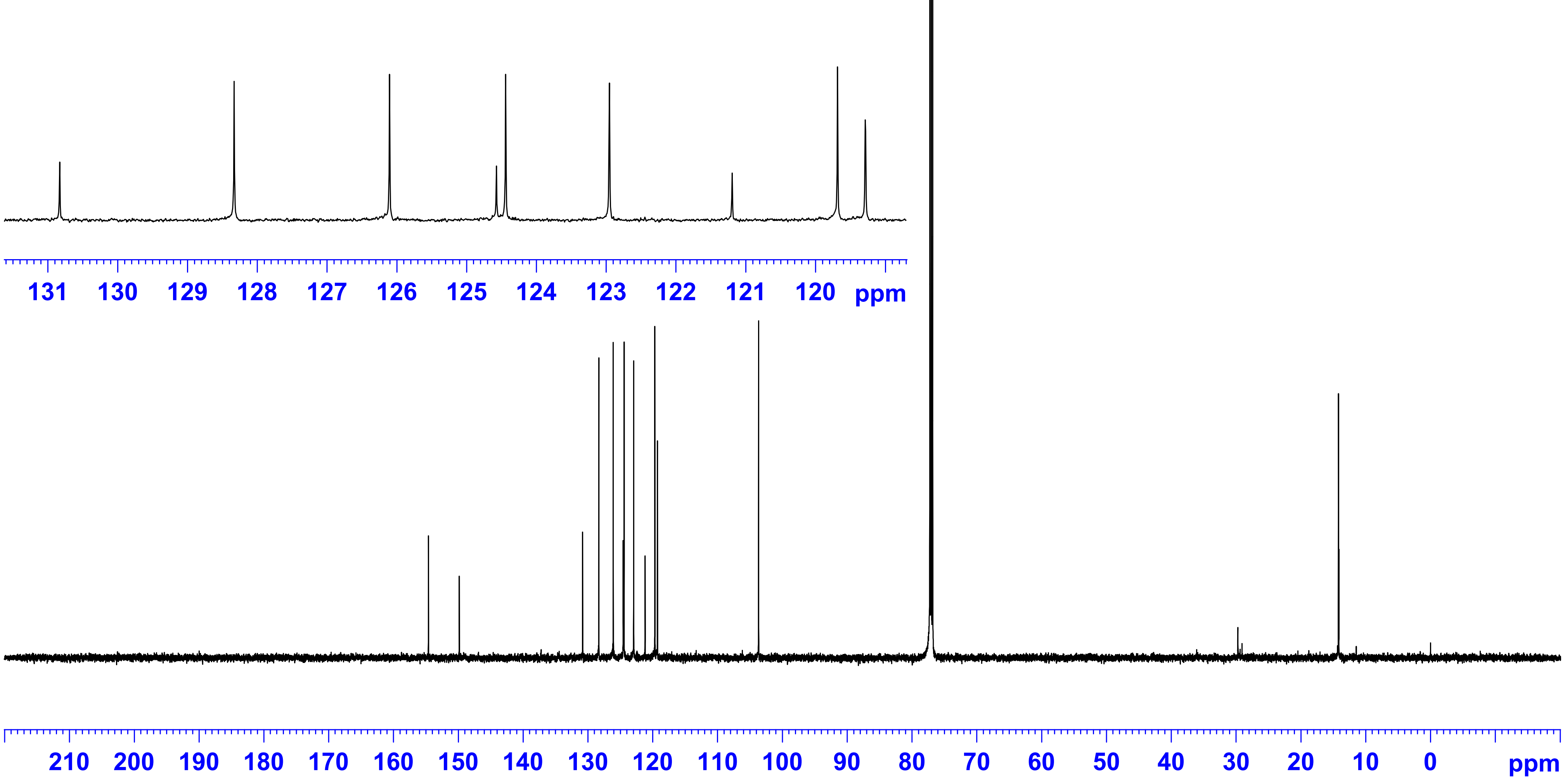
154.62
149.86
130.83
128.33
126.10
124.57
124.44
122.95
121.20
119.69
119.29
103.68
77.25
77.04
76.83
14.21
131 130 129 128 127 126 125 124 123 122 121 120 ppm
210 200 190 180 170 160 150 140 130 120 110 100 90 80 70 60 50 40 30 20 10 0 ppm

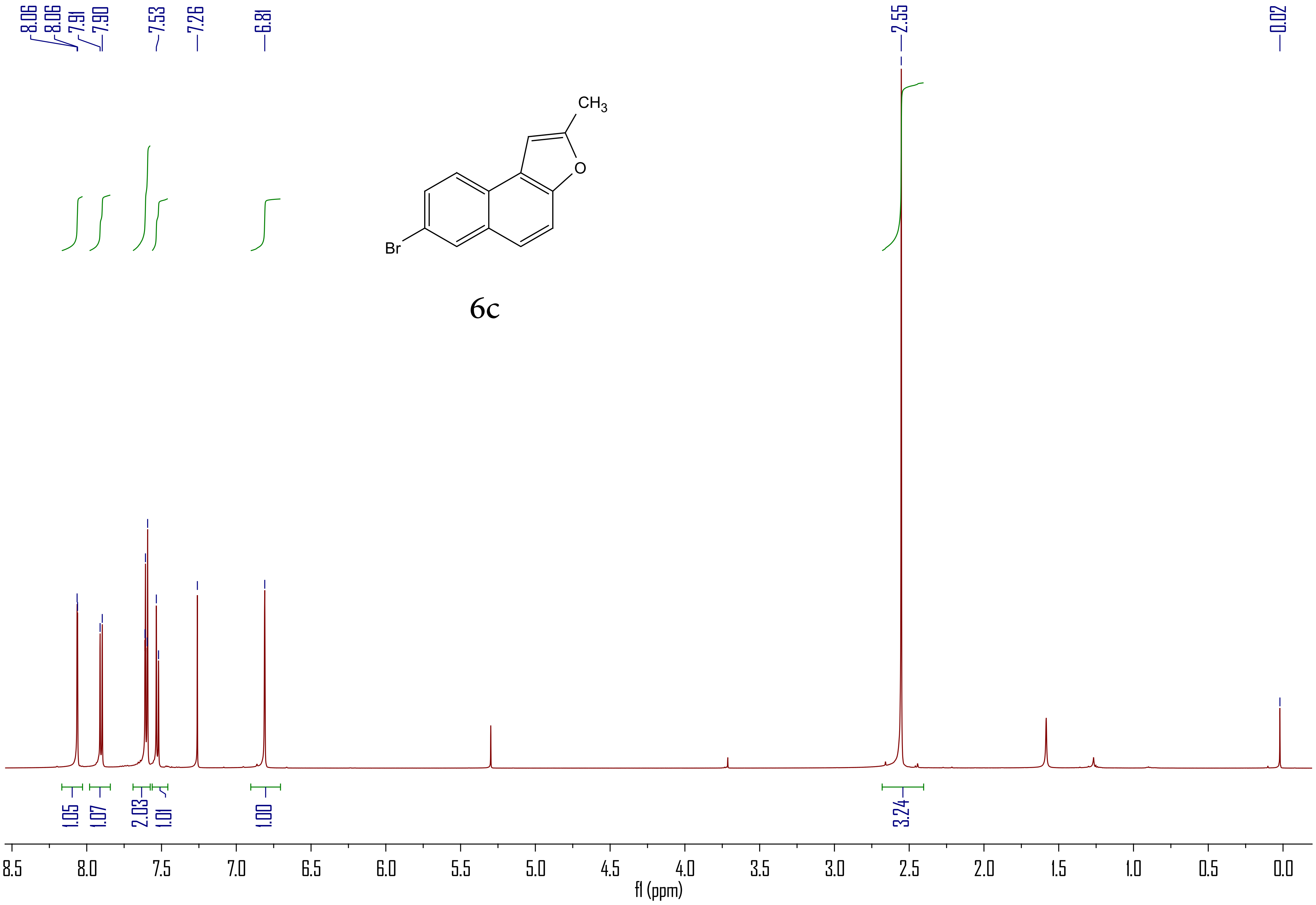

8.06
8.06
7.91
7.90
7.53
7.26
6.81
2.55
0.02
CH3
O
Br
6c
1.05
1.07
2.03
1.01
1.00
3.24
8.5 8.0 7.5 7.0 6.5 6.0 5.5 5.0 4.5 4.0 3.5 3.0 2.5 2.0 1.5 1.0 0.5 0.0
f1 (ppm)

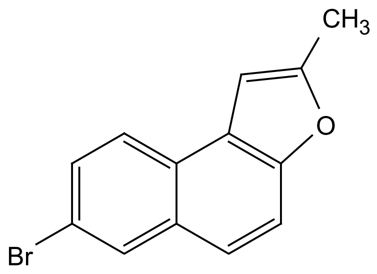
CH3
O
Br

6c

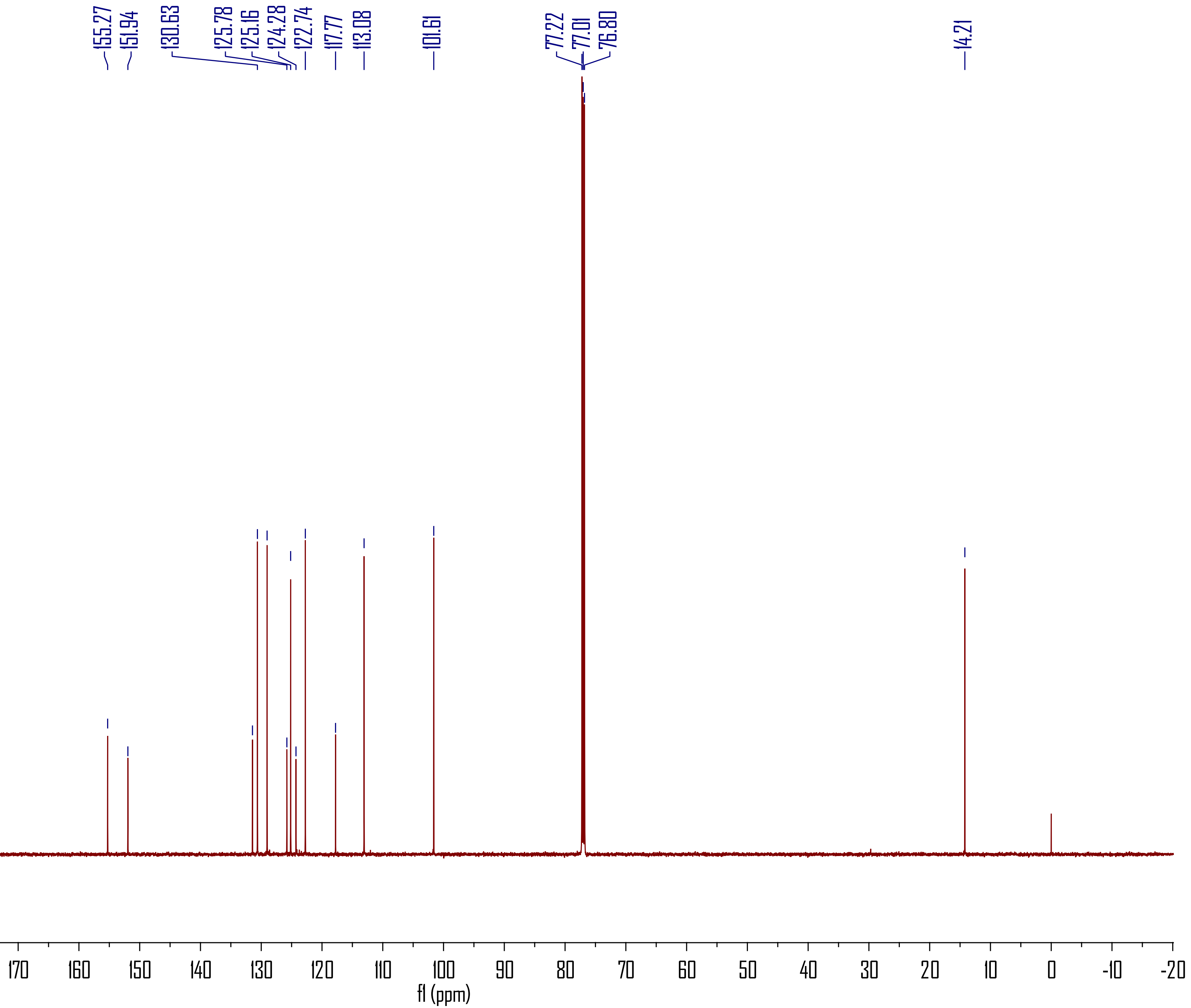
155.27
151.94
130.63
125.78
125.16
124.28
122.74
117.77
113.08
101.61
77.22
77.01
76.80
14.21
220 210 200 190 180 170 160 150 140 130 120 110 100 90 80 70 60 50 40 30 20 10 0 -10 -20
f1 (ppm)

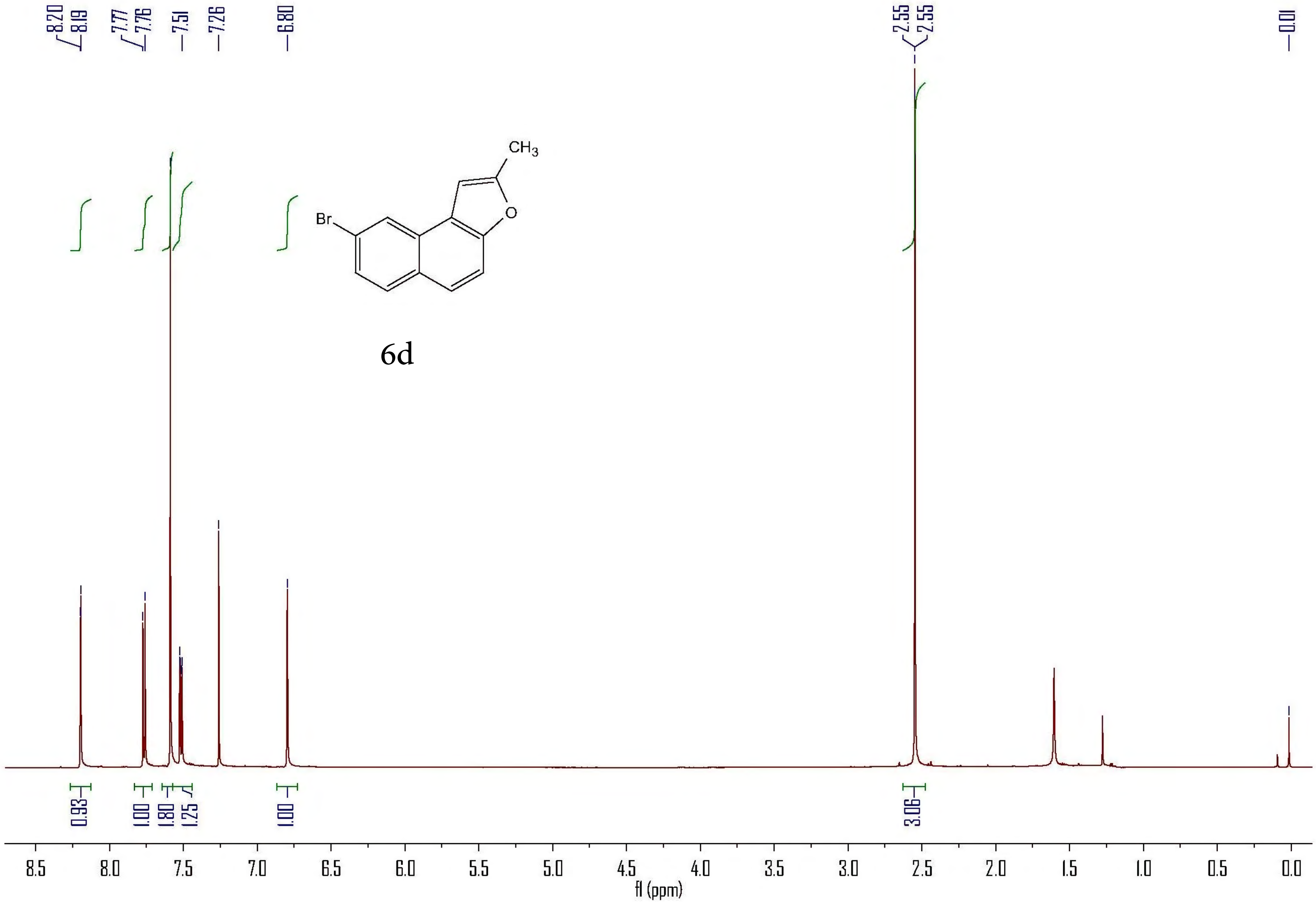

8.20
8.19
7.77
7.76
7.51
7.26
6.80
2.55
2.55
0.01
CH3
Br
O
6d
0.93
1.00
1.80
1.25
1.00
3.06
8.5 8.0 7.5 7.0 6.5 6.0 5.5 5.0 4.5 4.0 3.5 3.0 2.5 2.0 1.5 1.0 0.5 0.0
f1 (ppm)

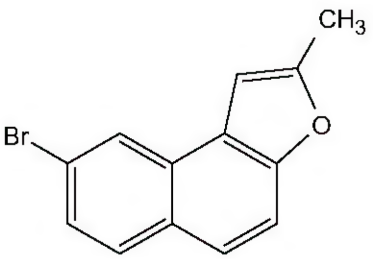
CH3
Br
O

6d

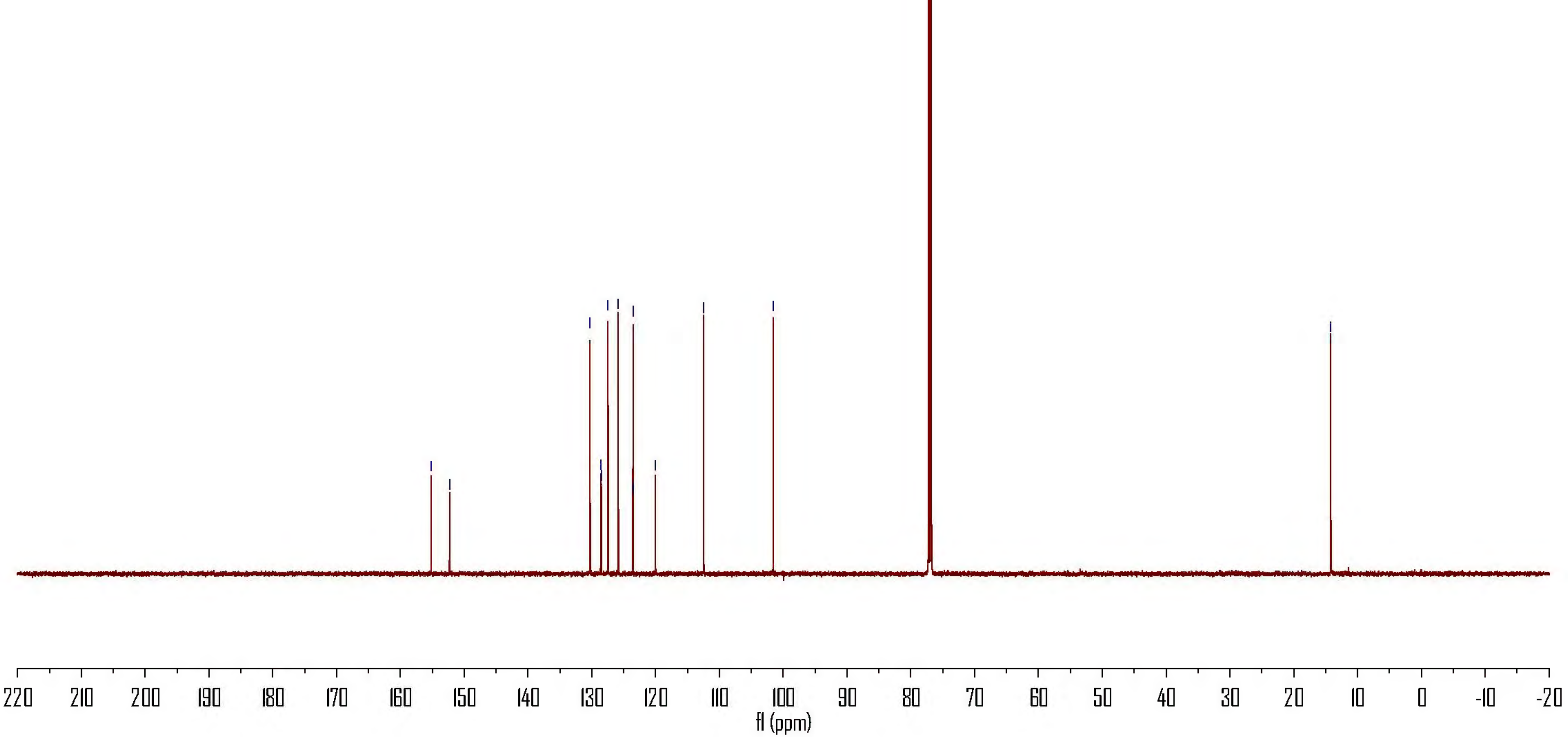
155.20
152.31
127.46
125.85
123.55
123.51
120.04
112.47
101.57
77.23
77.02
76.80
14.23
220 210 200 190 180 170 160 150 140 130 120 110 100 90 80 70 60 50 40 30 20 10 0 -10 -20
f1 (ppm)

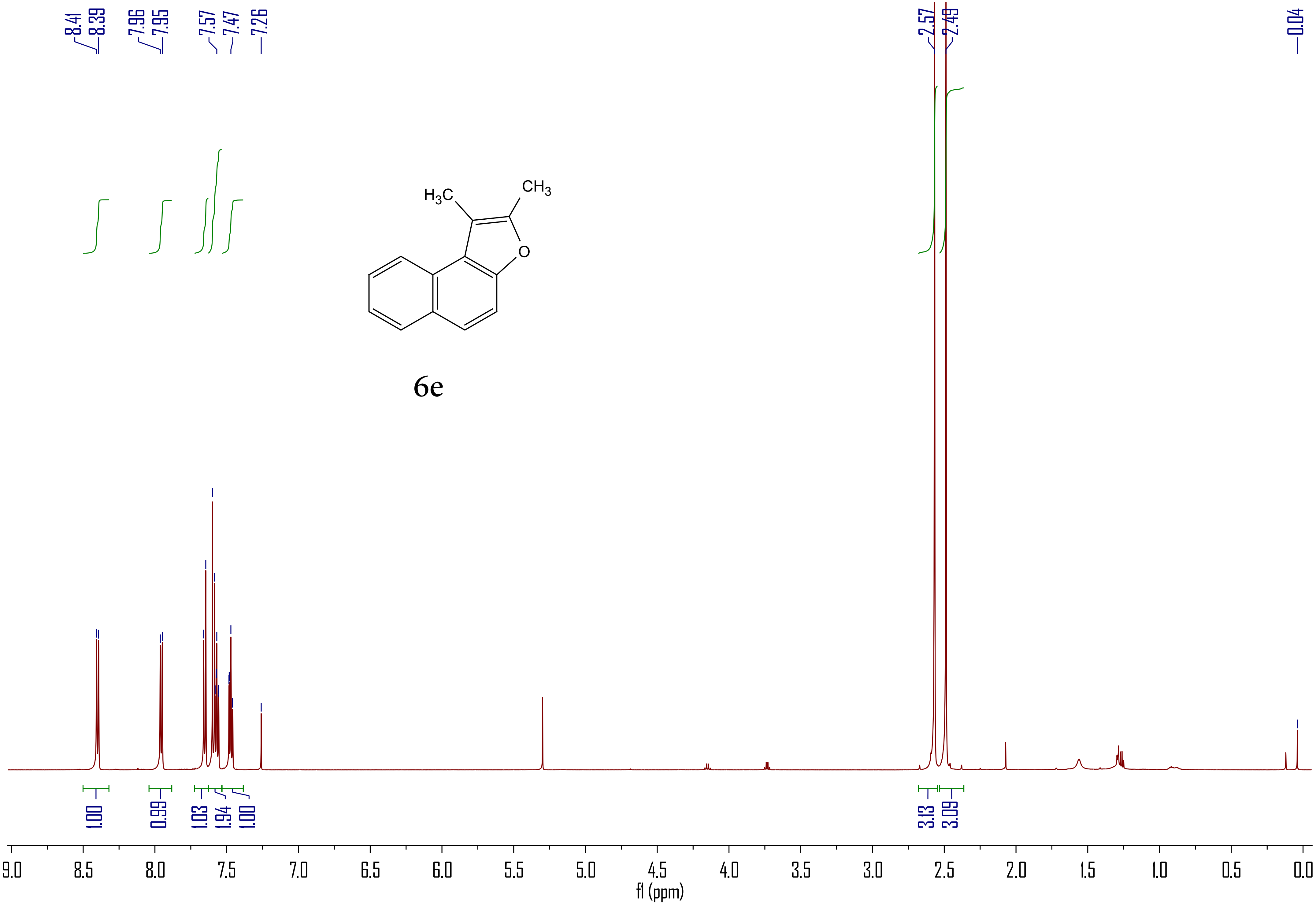
8.41
8.39
7.96
7.95
7.57
7.47
7.26
2.57
2.49
0.04
H3C
CH3
O
1.00
0.99
1.03
1.94
1.00
3.13
3.09
9.0
8.5
8.0
7.5
7.0
6.5
6.0
5.5
5.0
4.5
4.0
3.5
3.0
2.5
2.0
1.5
1.0
0.5
0.0
f1 (ppm)

6e

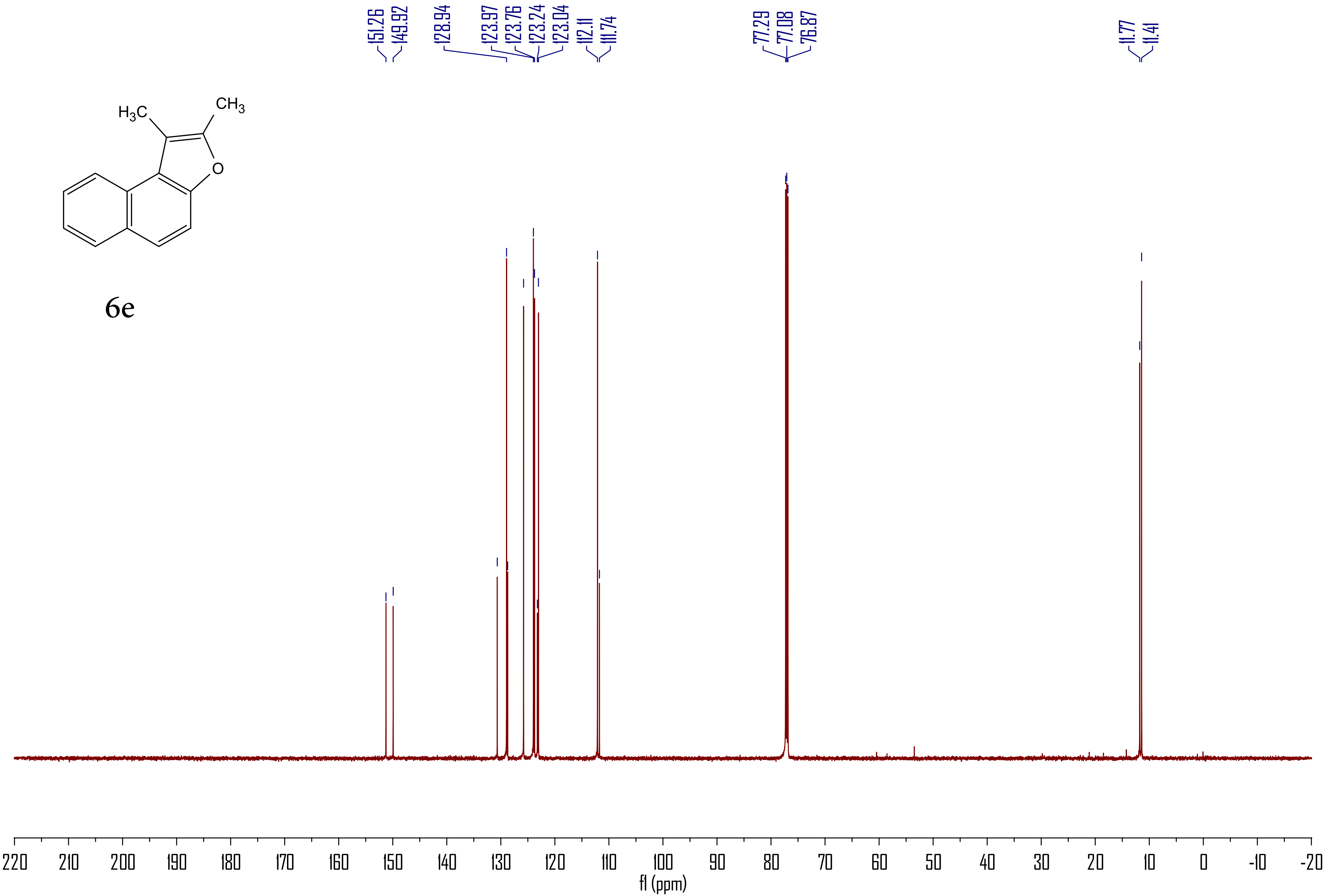
151.26
149.92
128.94
123.97
123.76
123.24
123.04
112.11
111.74
77.29
77.08
76.87
11.77
11.41
H3C
CH3
O
220 210 200 190 180 170 160 150 140 130 120 110 100 90 80 70 60 50 40 30 20 10 0 -10 -20
f1 (ppm)

6e

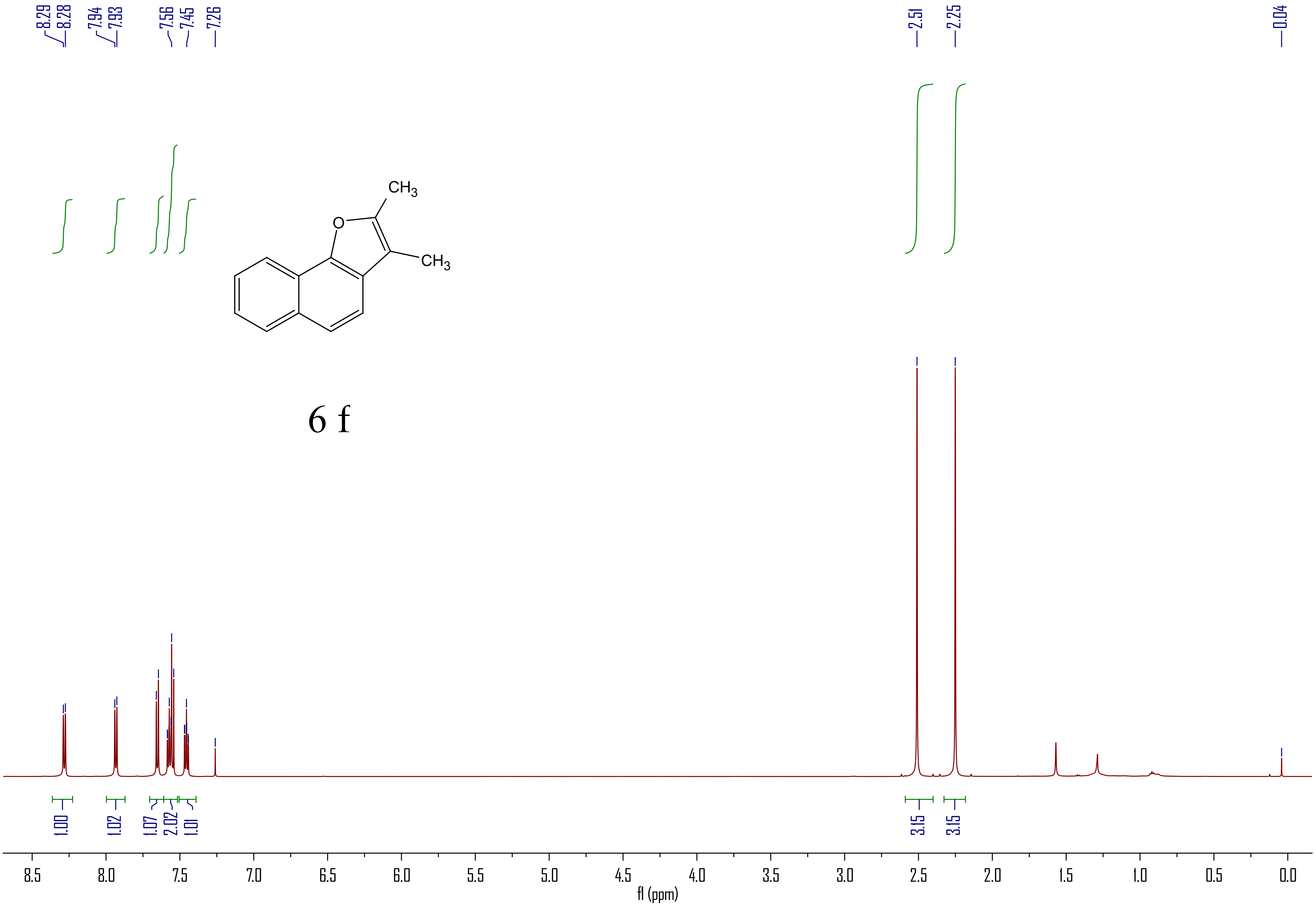
8.29
8.28
7.94
7.93
7.56
7.45
7.26
2.51
2.25
0.04
CH3
O
CH3
6 f
1.00
1.02
1.07
2.02
1.01
3.15
3.15
8.5
8.0
7.5
7.0
6.5
6.0
5.5
5.0
4.5
4.0
3.5
3.0
2.5
2.0
1.5
1.0
0.5
0.0
f1 (ppm)

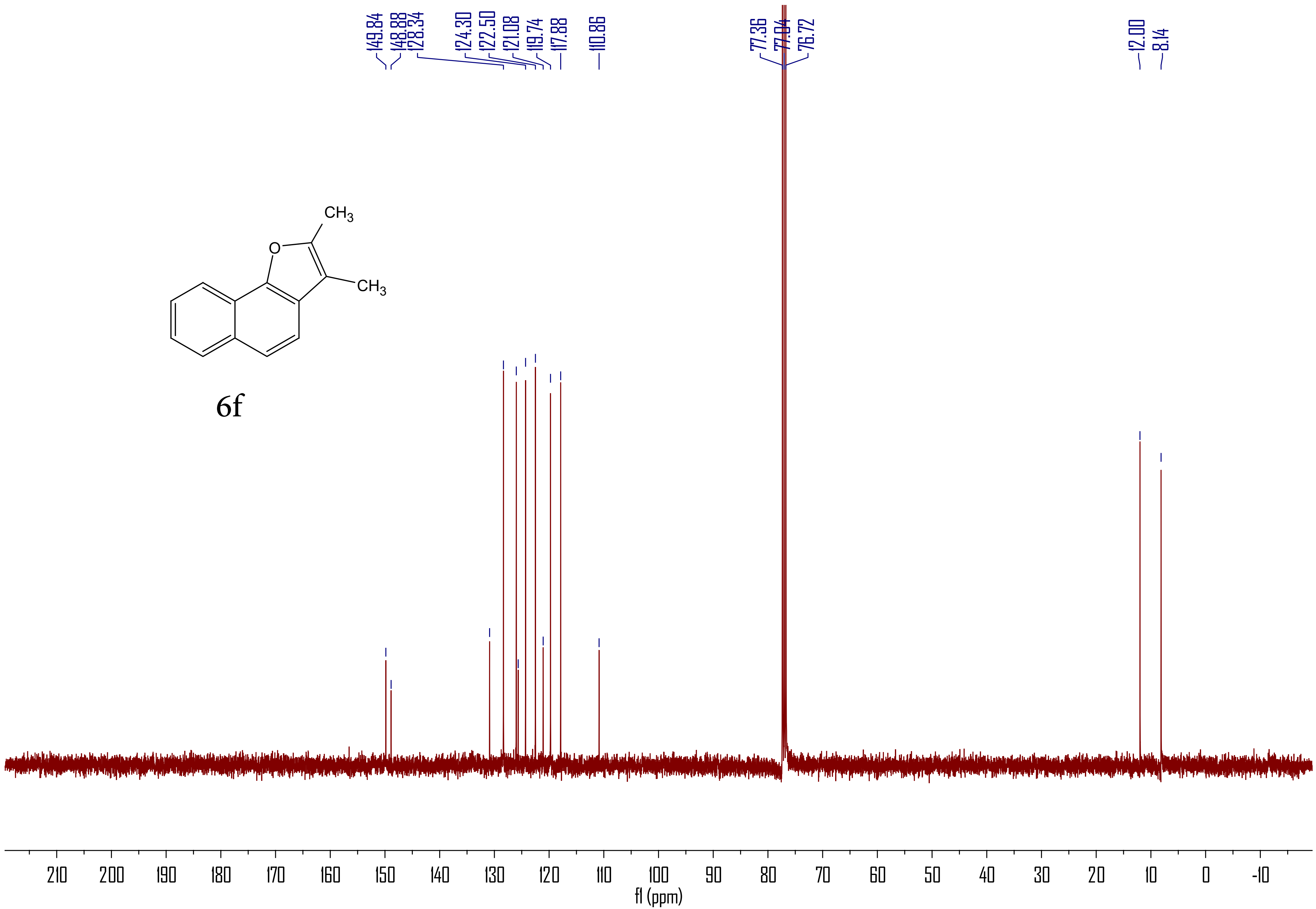
149.84
148.88
128.34
124.30
122.50
121.08
119.74
117.88
110.86
77.36
77.04
76.72
12.00
8.14
CH_3
O
CH_3
6f
210 200 190 180 170 160 150 140 130 120 110 100 90 80 70 60 50 40 30 20 10 0 -10
f1 (ppm)

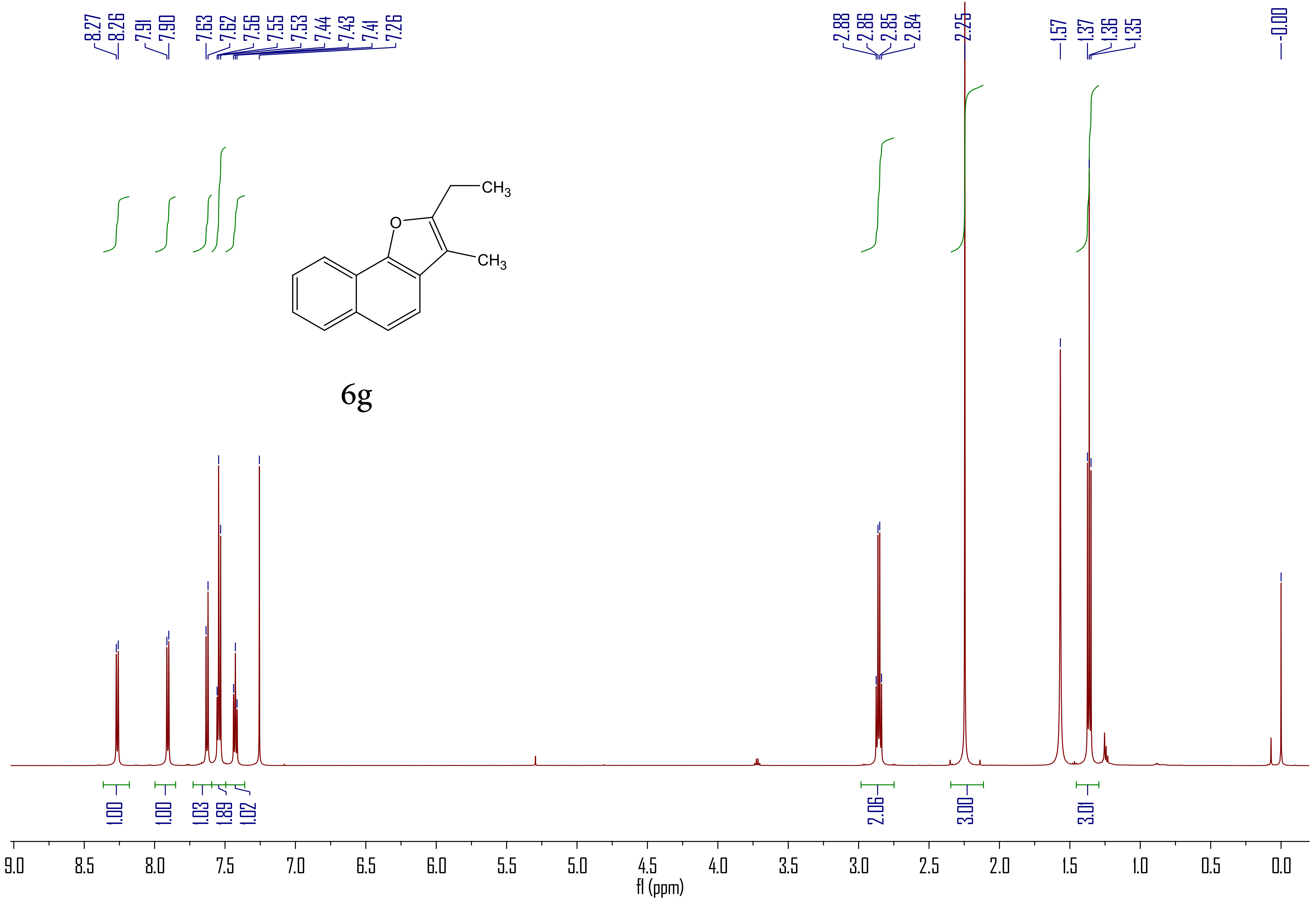
8.27
8.26
7.91
7.90
7.63
7.62
7.56
7.55
7.53
7.44
7.43
7.41
7.26
2.88
2.86
2.85
2.84
2.25
1.57
1.37
1.36
1.35
-0.00
O
CH3
CH3
6g
1.00
1.00
1.03
1.89
1.02
2.06
3.00
3.01
9.0
8.5
8.0
7.5
7.0
6.5
6.0
5.5
5.0
4.5
4.0
3.5
3.0
2.5
2.0
1.5
1.0
0.5
0.0
f1 (ppm)

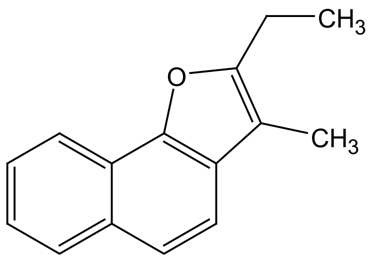
CH3
O
CH3

6g

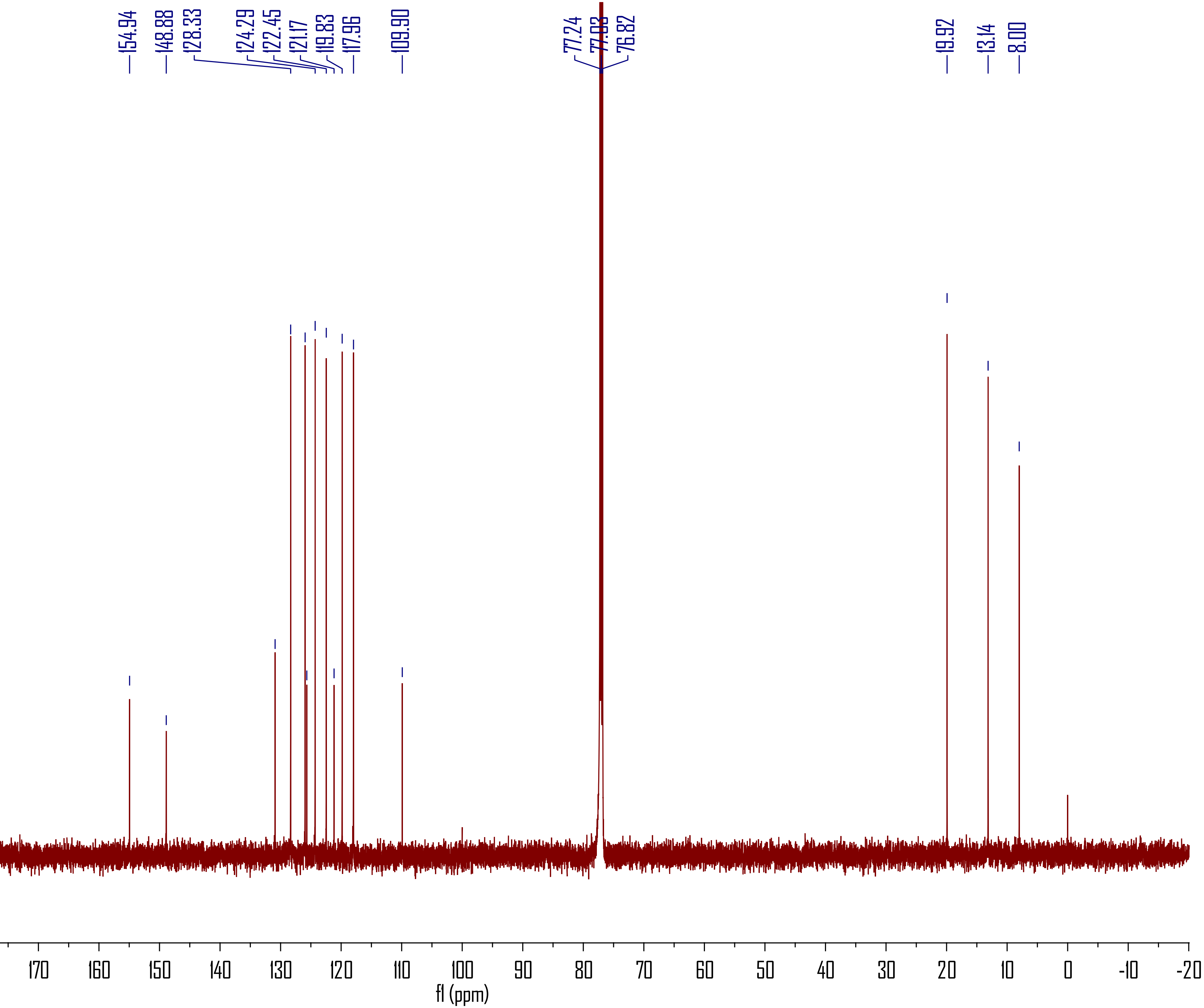
154.94
148.88
128.33
124.29
122.45
121.17
119.83
117.96
109.90
77.24
77.03
76.82
19.92
13.14
8.00
220 210 200 190 180 170 160 150 140 130 120 110 100 90 80 70 60 50 40 30 20 10 0 -10 -20
f1 (ppm)

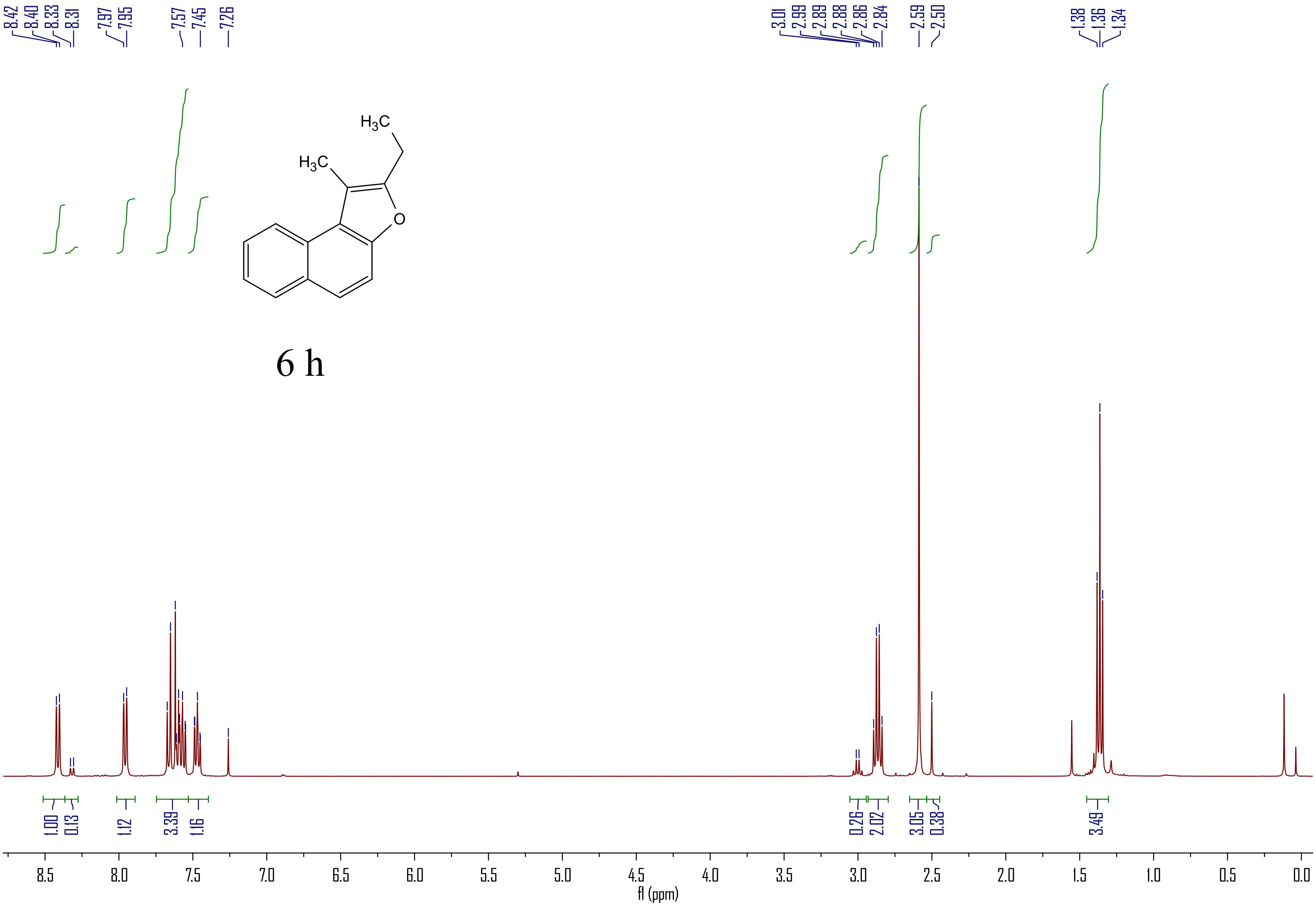
8.42
8.40
8.33
8.31
7.97
7.95
7.57
7.45
7.26
3.01
2.99
2.89
2.88
2.86
2.84
2.59
2.50
1.38
1.36
1.34
H_3C
H_3C
O
6 h
1.00
0.13
1.12
3.39
1.16
0.26
2.02
3.05
0.38
3.49
8.5
8.0
7.5
7.0
6.5
6.0
5.5
5.0
4.5
4.0
3.5
3.0
2.5
2.0
1.5
1.0
0.5
0.0
fl (ppm)

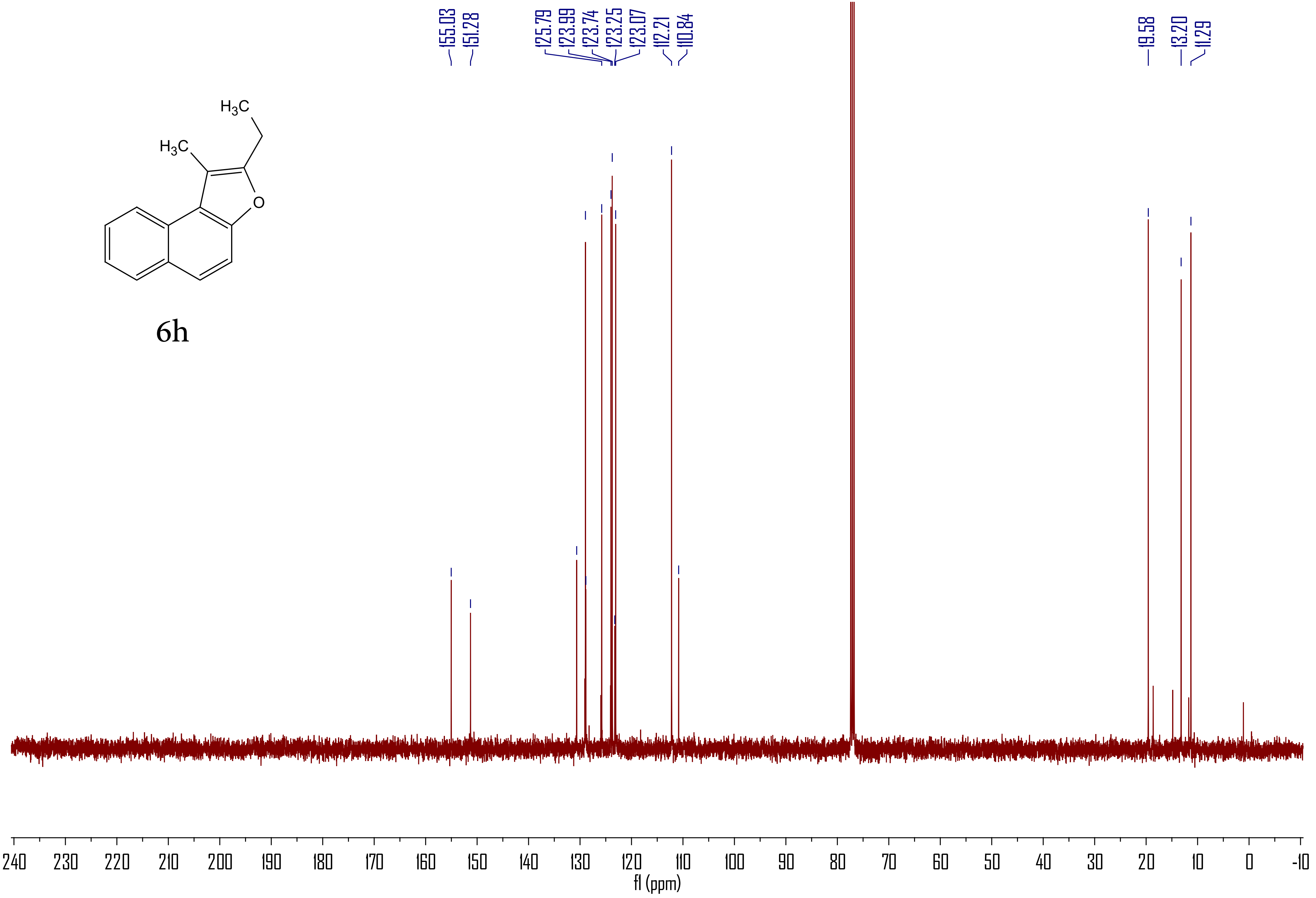
155.03
151.28
125.79
123.99
123.74
123.25
123.07
112.21
110.84
19.58
13.20
11.29
H_3C
H_3C
O
6h
240 230 220 210 200 190 180 170 160 150 140 130 120 110 100 90 80 70 60 50 40 30 20 10 0 -10
f1 (ppm)

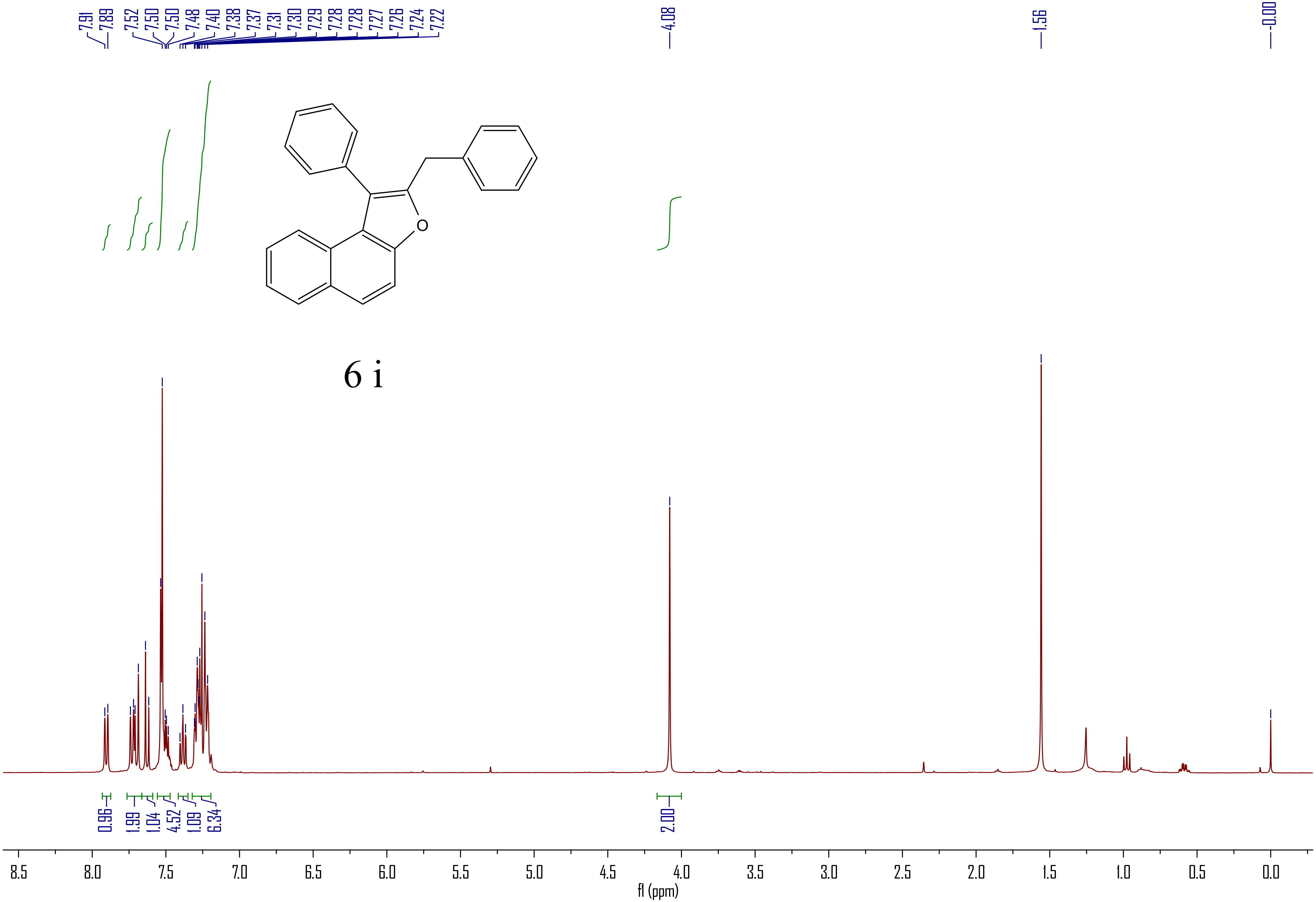

7.91
7.89
7.52
7.50
7.50
7.48
7.40
7.38
7.37
7.31
7.30
7.29
7.28
7.28
7.27
7.26
7.24
7.22
4.08
1.56
-0.00
O
6 i
0.96
1.99
1.04
4.52
1.09
6.34
2.00
8.5
8.0
7.5
7.0
6.5
6.0
5.5
5.0
4.5
4.0
3.5
3.0
2.5
2.0
1.5
1.0
0.5
0.0
f1 (ppm)

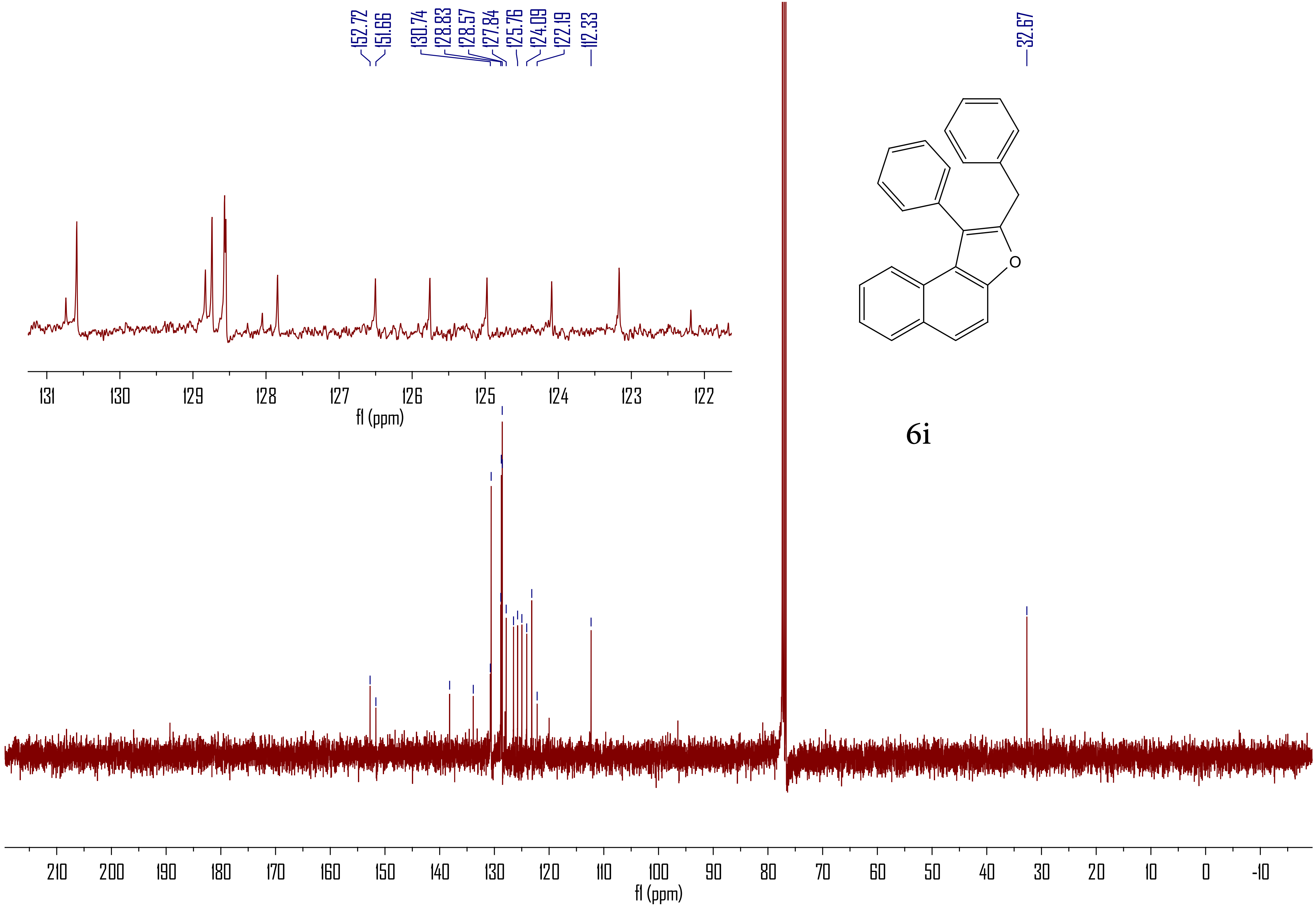
152.72
151.66
130.74
128.83
128.57
127.84
125.76
124.09
122.19
112.33
32.67
O
f1 (ppm)
f1 (ppm)

6i

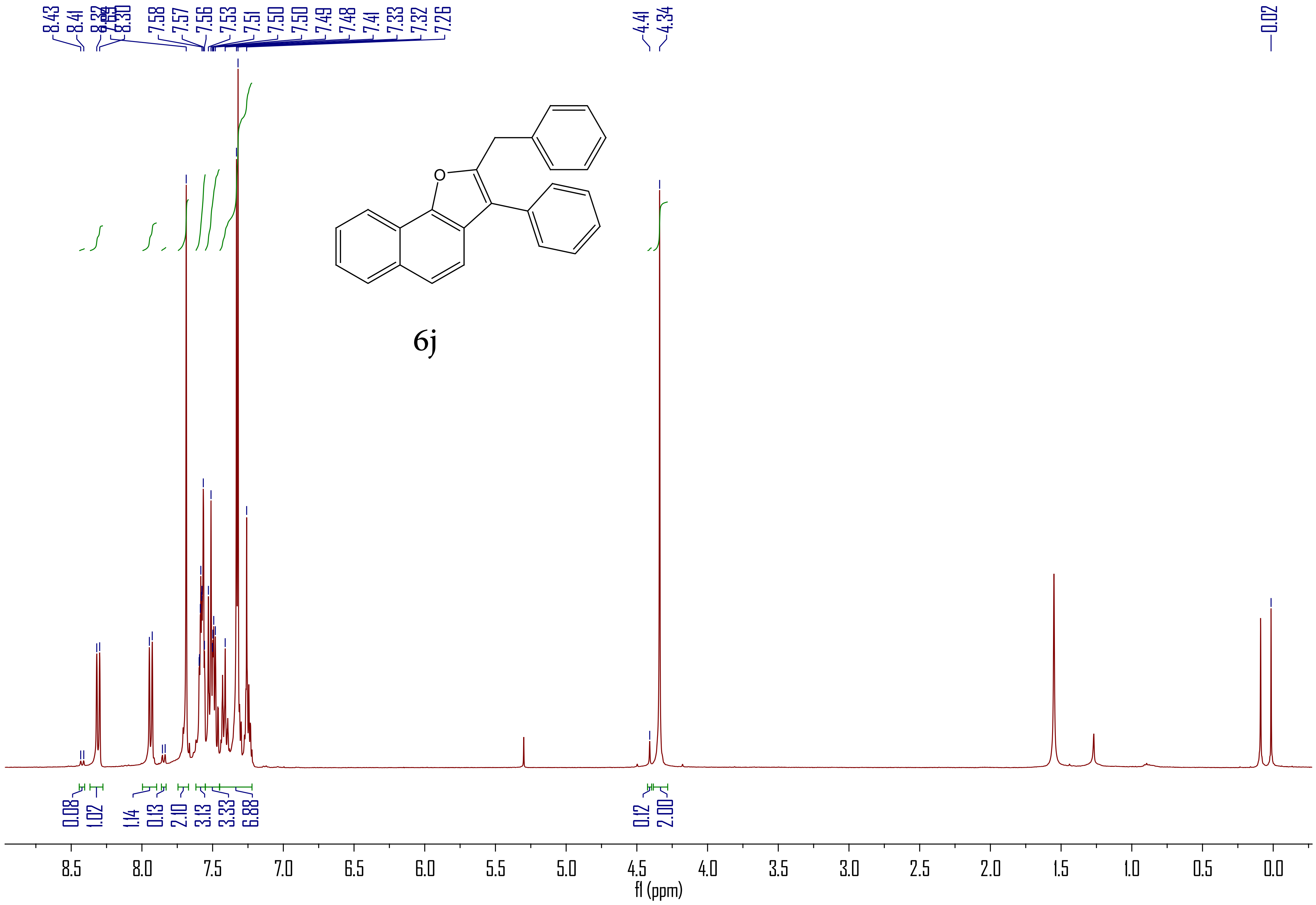
8.43
8.41
8.32
8.30
7.58
7.57
7.56
7.53
7.51
7.50
7.50
7.49
7.48
7.41
7.33
7.32
7.26
4.41
4.34
0.02
O
6j
0.08
1.02
1.14
0.13
2.10
3.13
3.33
6.88
0.12
2.00
8.5
8.0
7.5
7.0
6.5
6.0
5.5
5.0
4.5
4.0
3.5
3.0
2.5
2.0
1.5
1.0
0.5
0.0
f1 (ppm)

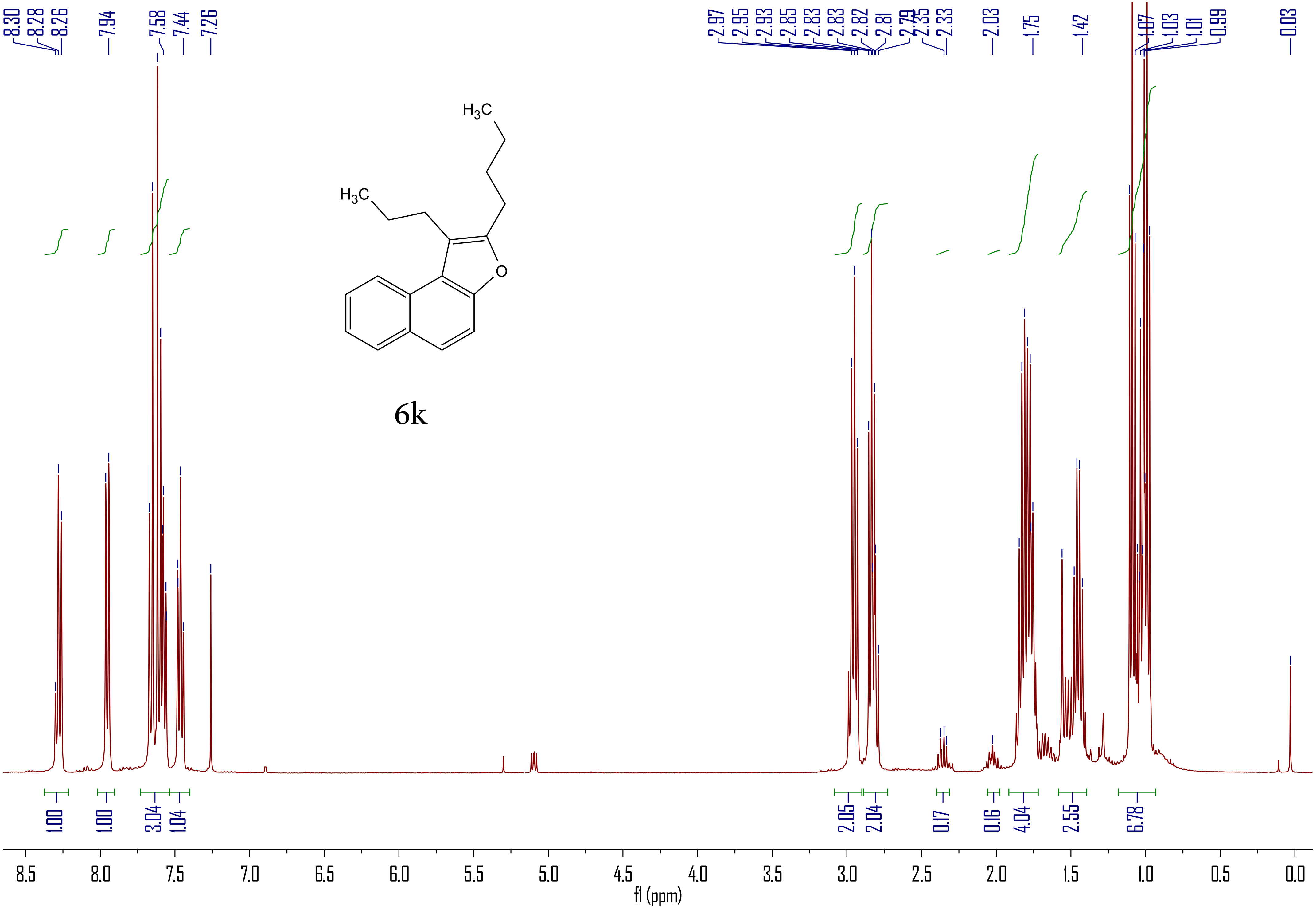
8.30
8.28
8.26
7.94
7.58
7.44
7.26
2.97
2.95
2.93
2.85
2.83
2.83
2.82
2.81
2.79
2.33
2.03
1.75
1.42
1.07
1.03
1.01
0.99
0.03
H3C
H3C
O
6k
1.00
1.00
3.04
1.04
2.05
2.04
0.17
0.16
4.04
2.55
6.78
8.5
8.0
7.5
7.0
6.5
6.0
5.5
5.0
4.5
4.0
3.5
3.0
2.5
2.0
1.5
1.0
0.5
0.0
f1 (ppm)

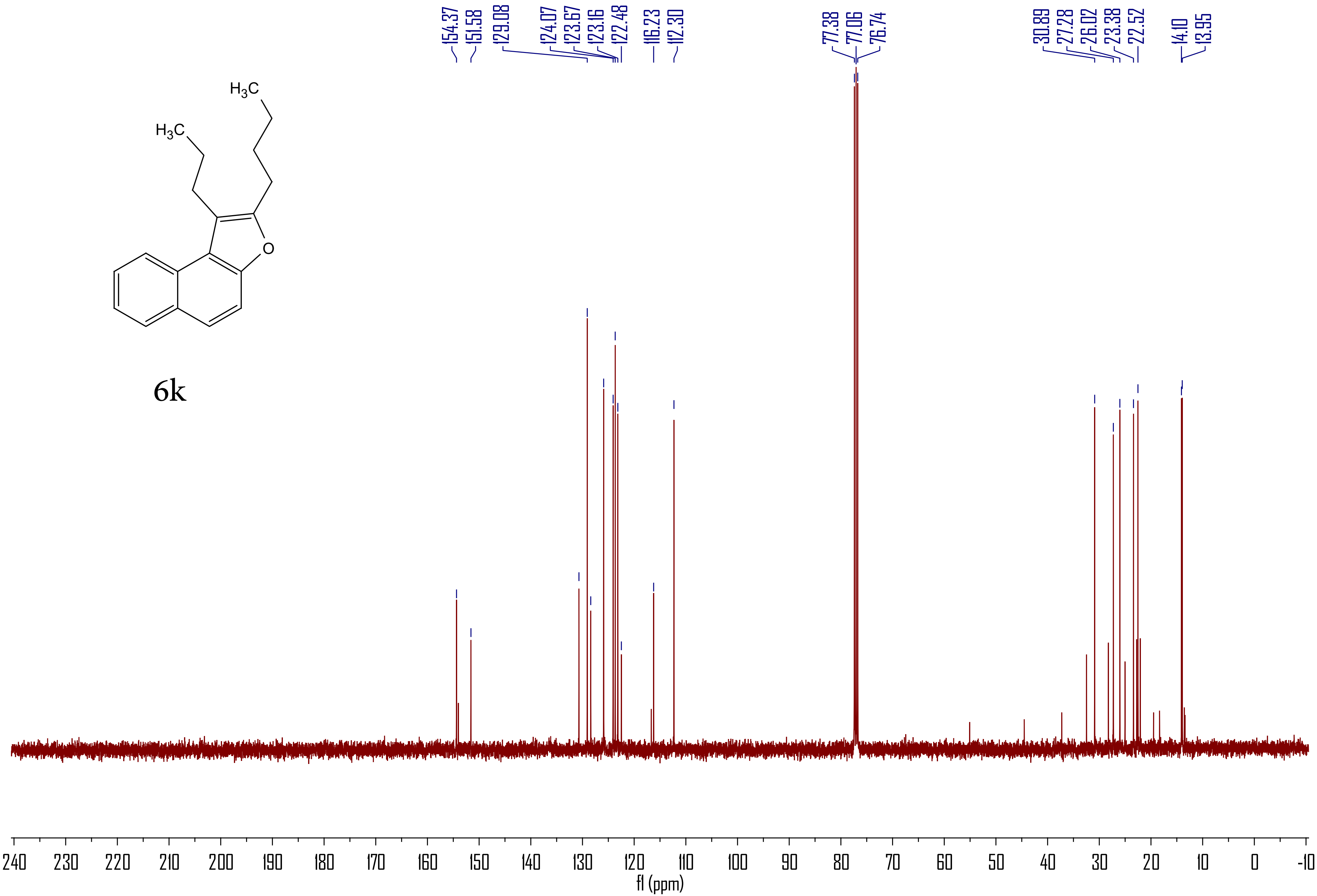

154.37
151.58
129.08
124.07
123.67
123.16
122.48
116.23
112.30
77.38
77.06
76.74
30.89
27.28
26.02
23.38
22.52
14.10
13.95
H3C
H3C
O
240 230 220 210 200 190 180 170 160 150 140 130 120 110 100 90 80 70 60 50 40 30 20 10 0 -10
f1 (ppm)

6k

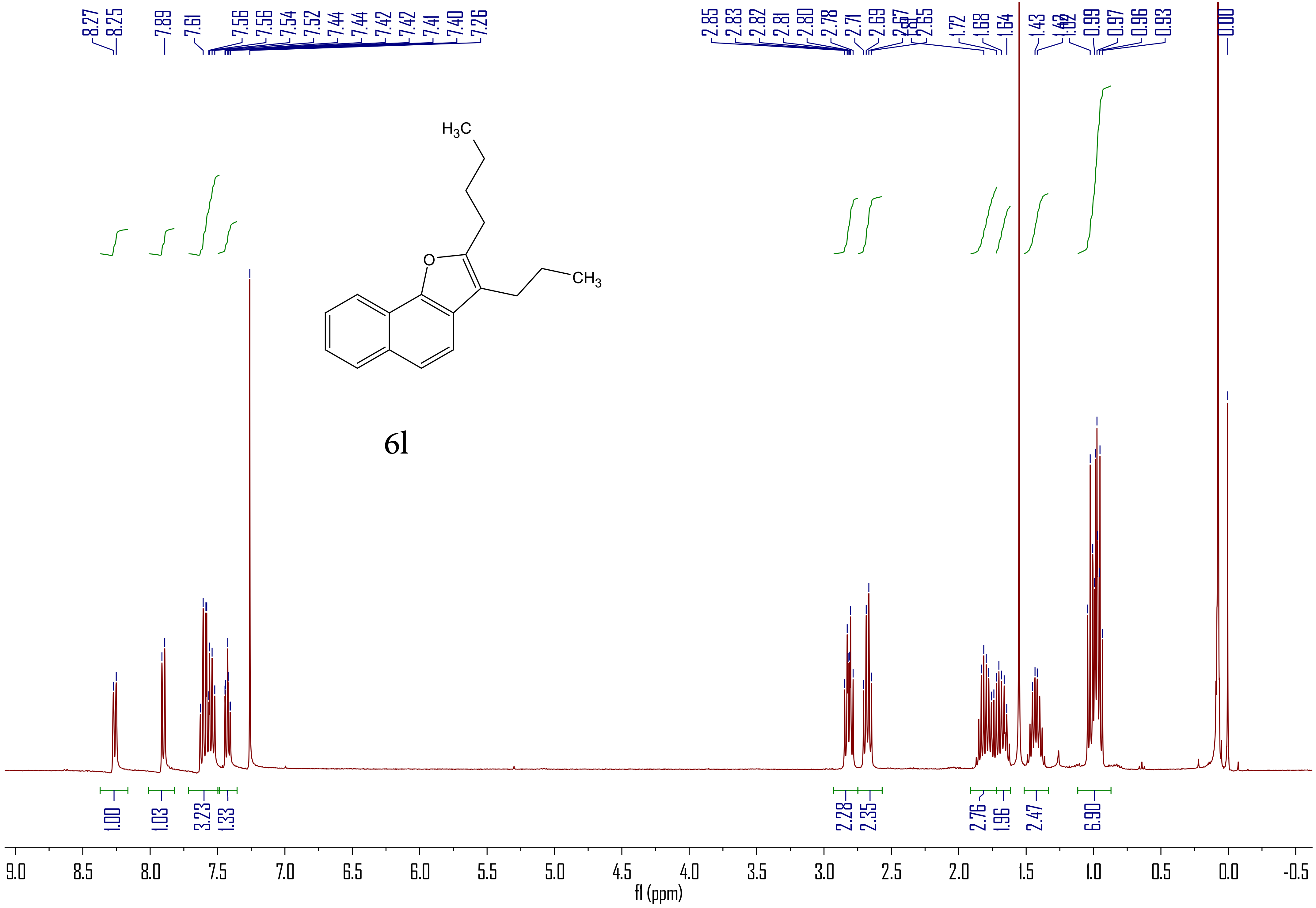
8.27
8.25
7.89
7.61
7.56
7.56
7.54
7.52
7.44
7.44
7.44
7.42
7.42
7.41
7.40
7.26
2.85
2.83
2.82
2.81
2.80
2.78
2.71
2.69
2.67
2.65
1.72
1.68
1.64
1.43
0.99
0.97
0.96
0.93
0.00
H3C
O
CH3
6l
1.00
1.03
3.23
1.33
2.28
2.35
2.76
1.96
2.47
6.90
9.0
8.5
8.0
7.5
7.0
6.5
6.0
5.5
5.0
4.5
4.0
3.5
3.0
2.5
2.0
1.5
1.0
0.5
0.0
-0.5
f1 (ppm)

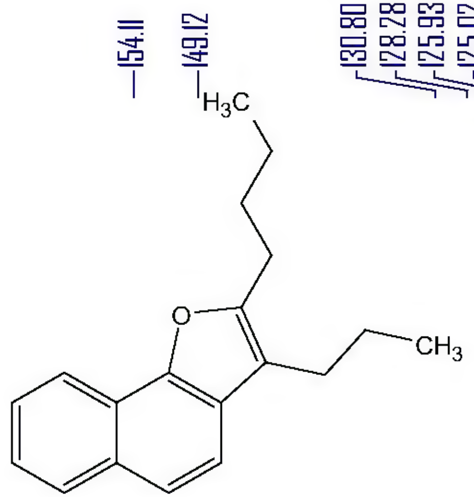

H3C
O
CH3

6l

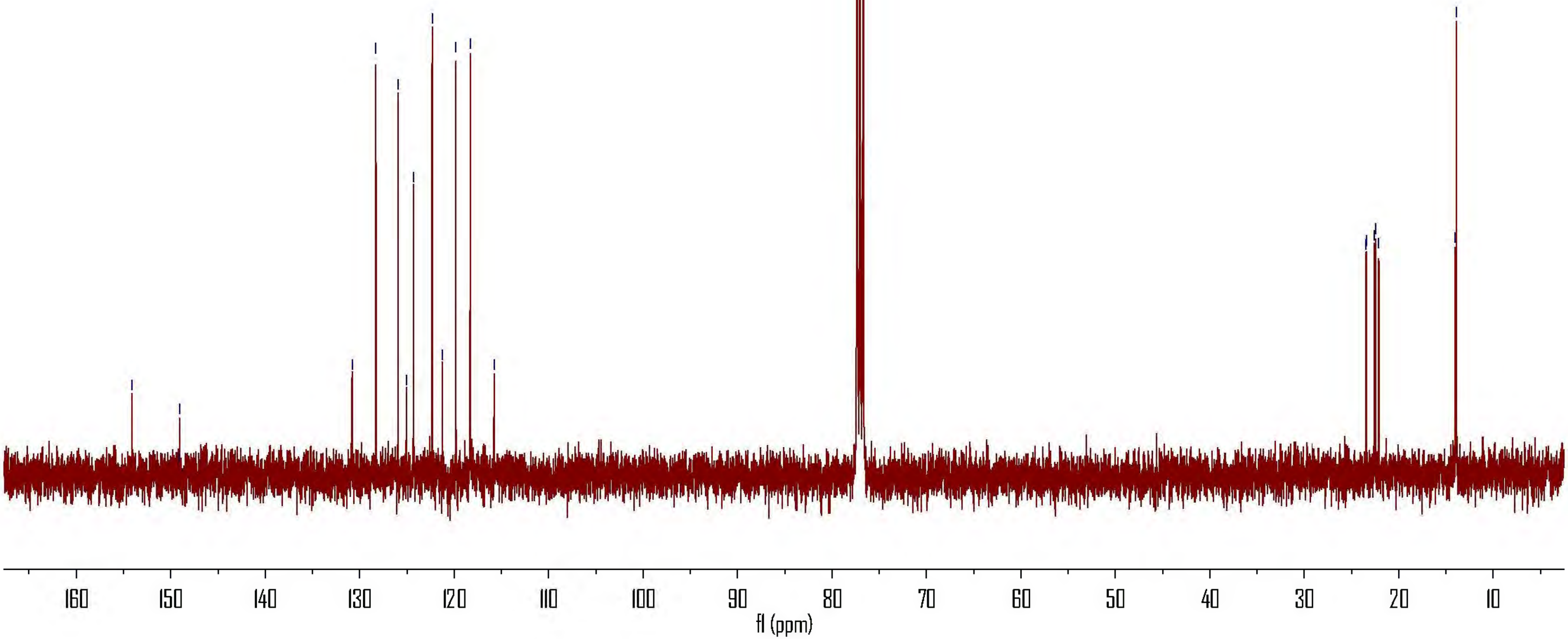

154.11
149.12
130.80
128.28
125.93
125.02
124.30
122.32
121.23
119.82
118.30
115.78
77.34
77.03
76.71
23.46
23.41
22.60
22.47
22.12
14.05
13.92
160
150
140
130
120
110
100
90
80
70
60
50
40
30
20
10
f1 (ppm)

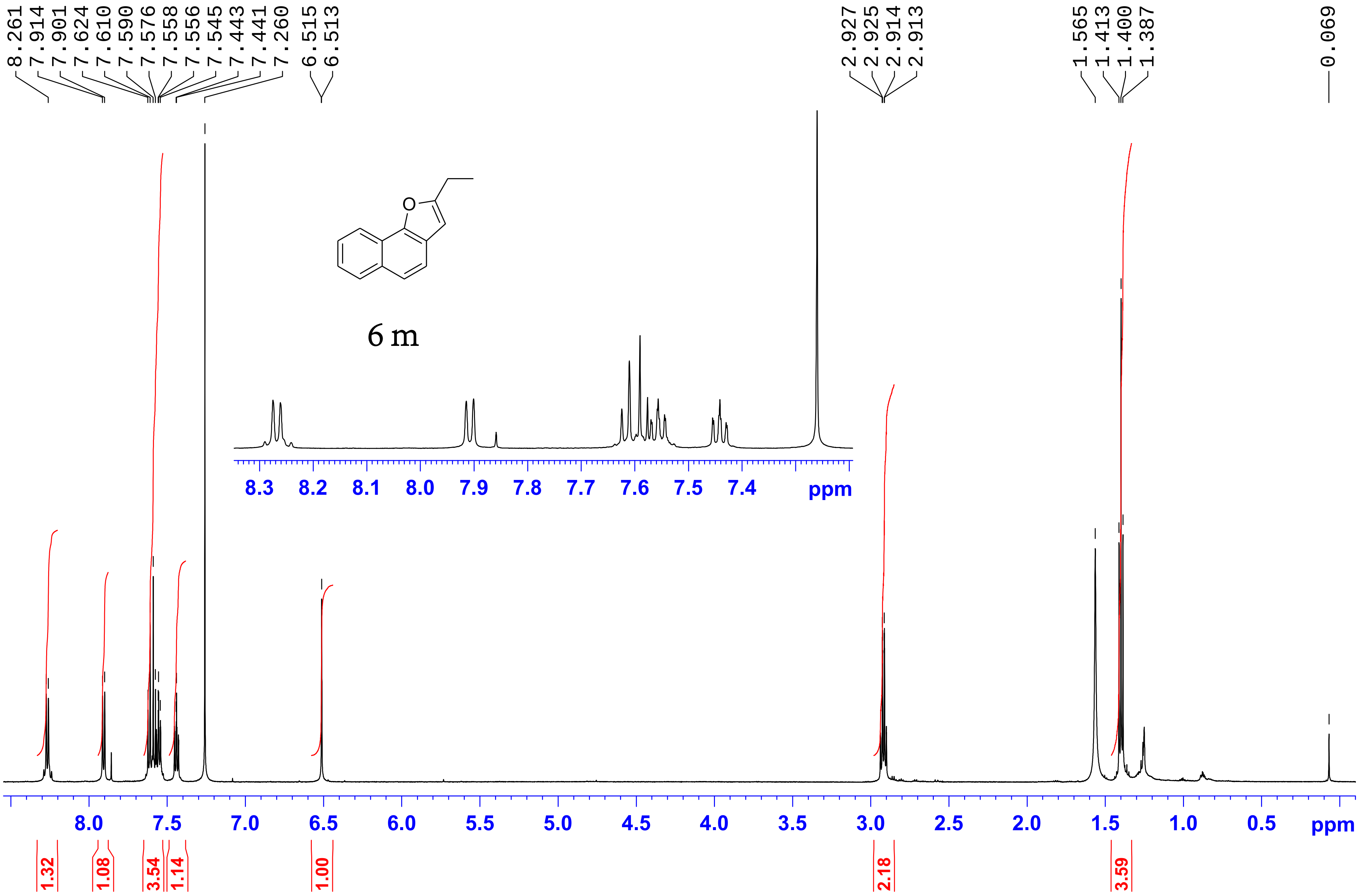

8.261
7.914
7.901
7.624
7.610
7.590
7.576
7.558
7.556
7.545
7.443
7.441
7.260
6.515
6.513
2.927
2.925
2.914
2.913
1.565
1.413
1.400
1.387
0.069
O
6 m
8.3 8.2 8.1 8.0 7.9 7.8 7.7 7.6 7.5 7.4 ppm
8.0 7.5 7.0 6.5 6.0 5.5 5.0 4.5 4.0 3.5 3.0 2.5 2.0 1.5 1.0 0.5 ppm
1.32
1.08
3.54
1.14
1.00
2.18
3.59

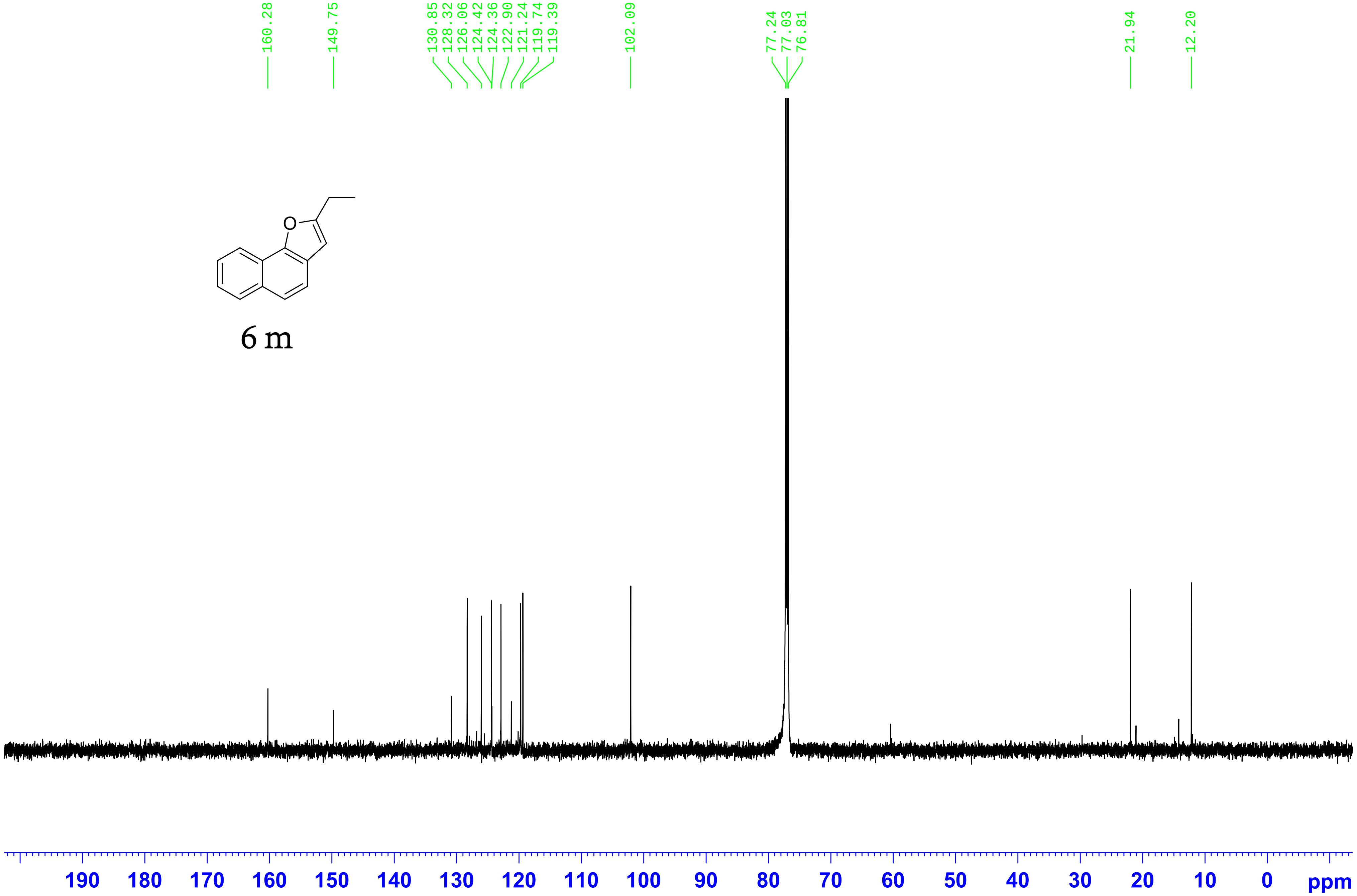
160.28
149.75
130.85
128.32
126.06
124.42
124.36
122.90
121.24
119.74
119.39
102.09
77.24
77.03
76.81
21.94
12.20
O
6 m
190 180 170 160 150 140 130 120 110 100 90 80 70 60 50 40 30 20 10 0 ppm

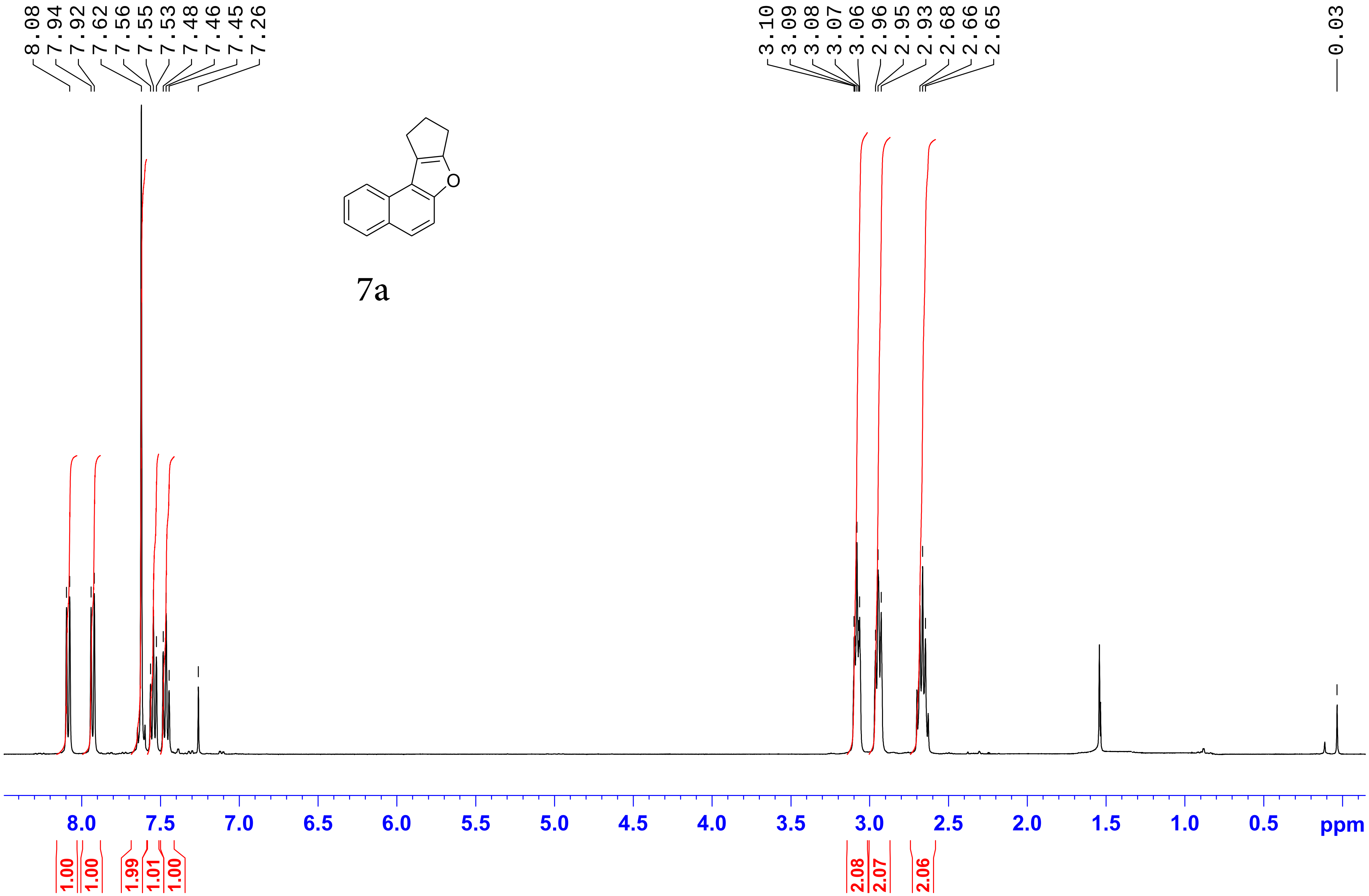

8.08
7.94
7.92
7.62
7.56
7.55
7.53
7.48
7.46
7.45
7.26
3.10
3.09
3.08
3.07
3.06
2.96
2.95
2.93
2.68
2.66
2.65
0.03
O
7a
8.0
7.5
7.0
6.5
6.0
5.5
5.0
4.5
4.0
3.5
3.0
2.5
2.0
1.5
1.0
0.5
ppm
1.00
1.00
1.99
1.01
1.00
2.08
2.07
2.06

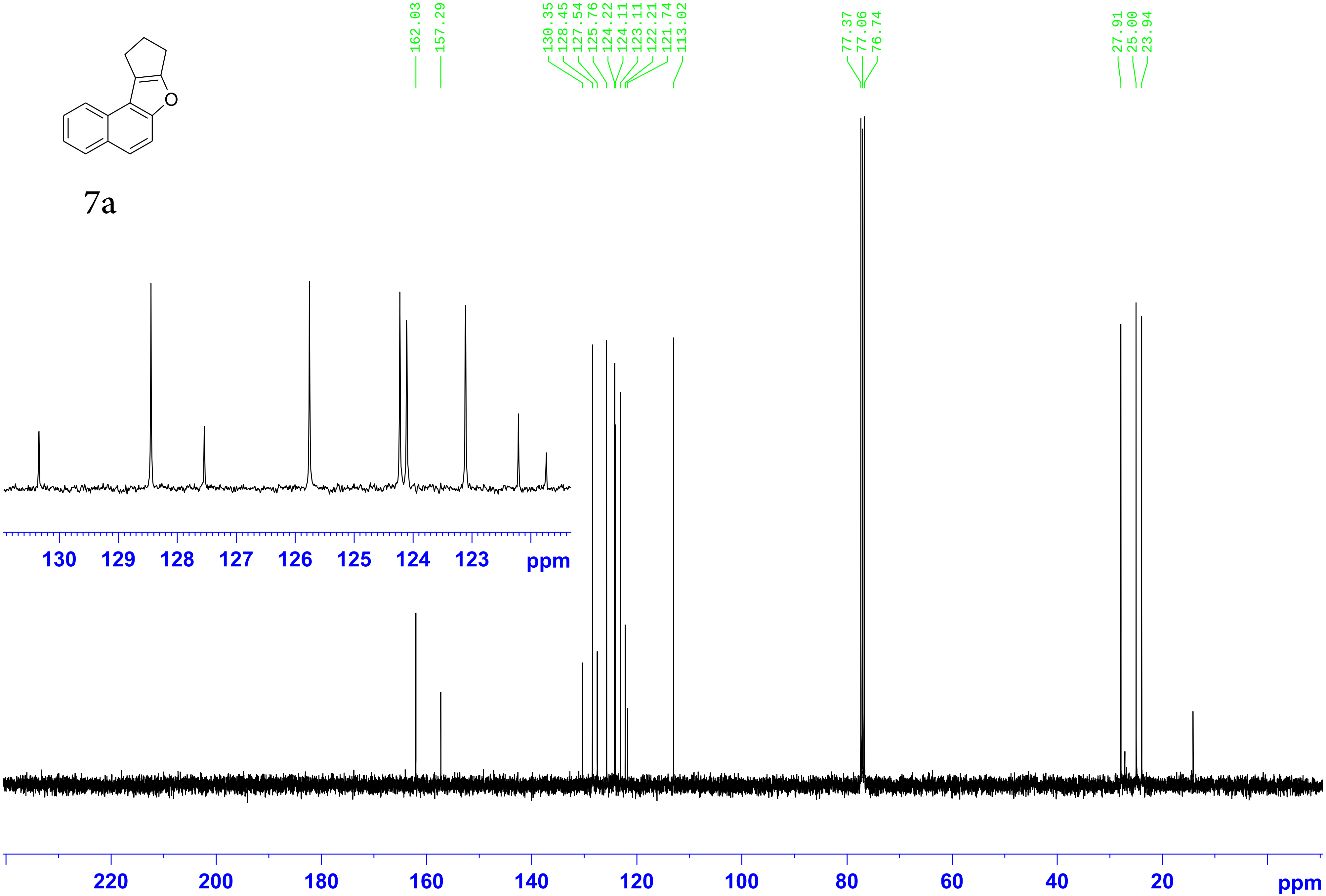
162.03
157.29
130.35
128.45
127.54
125.76
124.22
124.11
123.11
122.21
121.74
113.02
77.37
77.06
76.74
27.91
25.00
23.94
O
130 129 128 127 126 125 124 123 ppm
220 200 180 160 140 120 100 80 60 40 20 ppm

7a

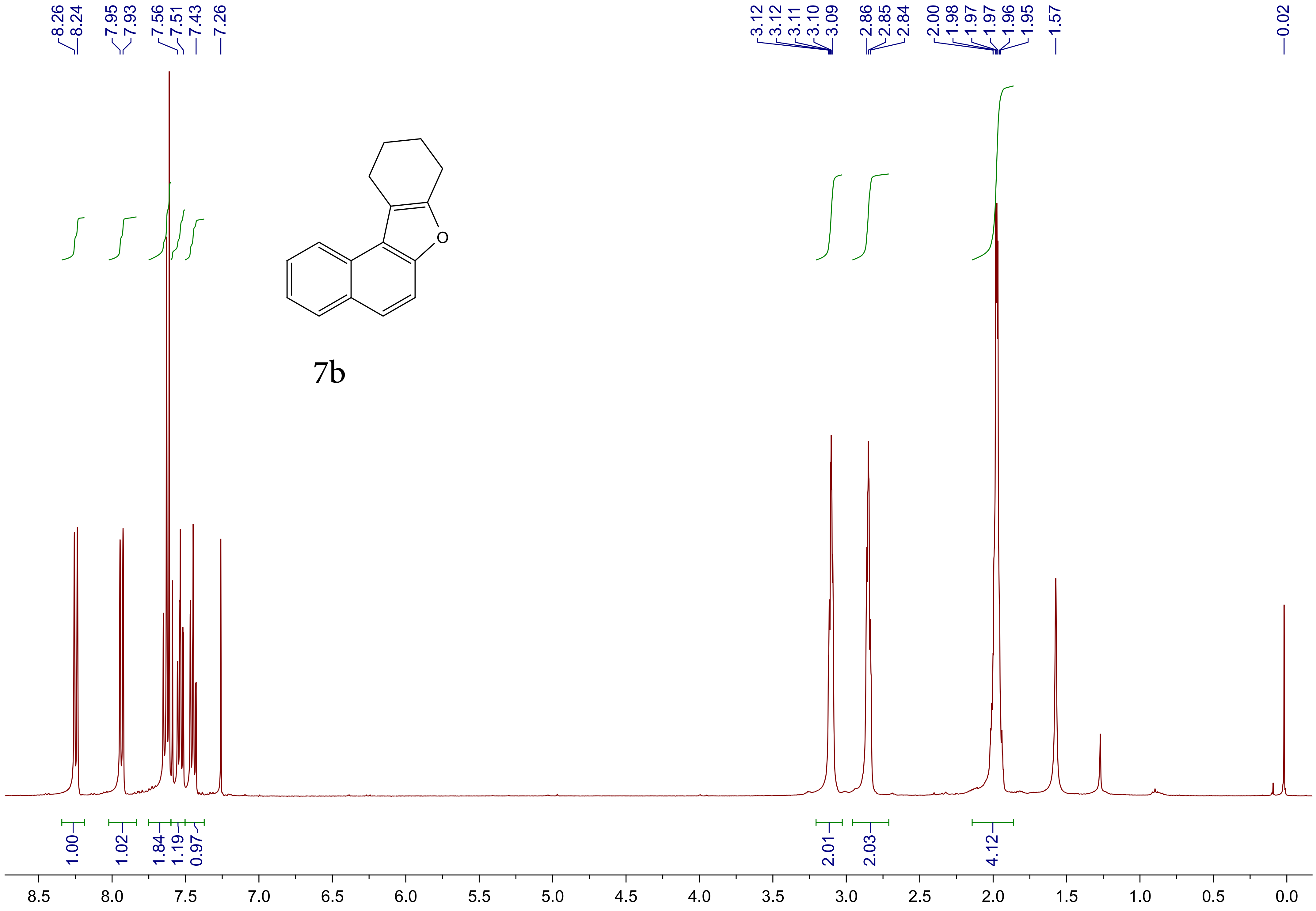
8.26
8.24
7.95
7.93
7.56
7.51
7.43
7.26
3.12
3.12
3.11
3.10
3.09
2.86
2.85
2.84
2.00
1.98
1.97
1.97
1.96
1.95
1.57
-0.02
O
7b
1.00
1.02
1.84
1.19
0.97
2.01
2.03
4.12
8.5
8.0
7.5
7.0
6.5
6.0
5.5
5.0
4.5
4.0
3.5
3.0
2.5
2.0
1.5
1.0
0.5
0.0

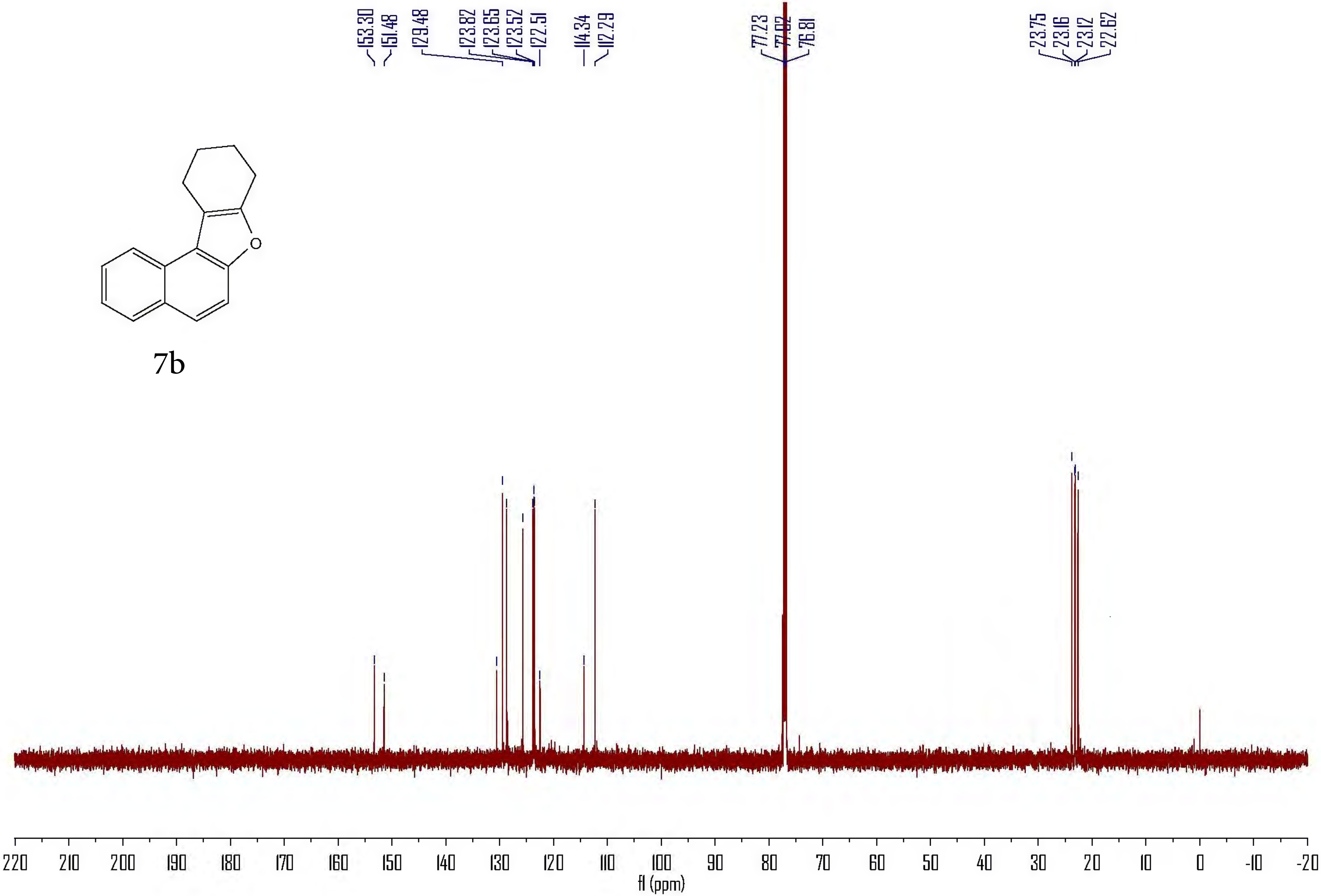
153.30
151.48
129.48
123.82
123.65
123.52
122.51
114.34
112.29
77.23
77.02
76.81
23.75
23.16
23.12
22.62
7b
220 210 200 190 180 170 160 150 140 130 120 110 100 90 80 70 60 50 40 30 20 10 0 -10 -20
f1 (ppm)

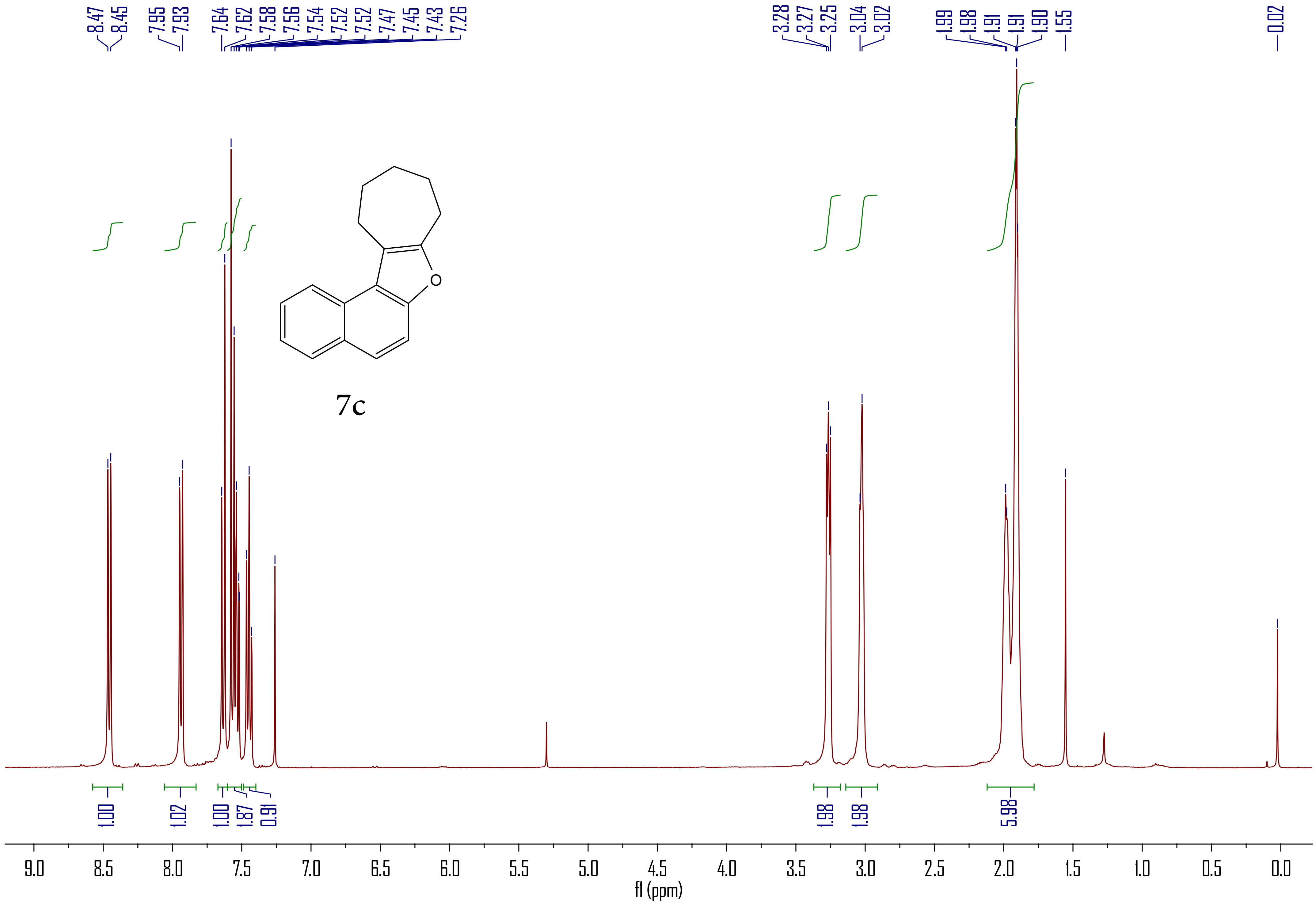
8.47
8.45
7.95
7.93
7.64
7.62
7.58
7.56
7.54
7.52
7.52
7.47
7.45
7.43
7.26
3.28
3.27
3.25
3.04
3.02
1.99
1.98
1.91
1.91
1.90
1.55
0.02
O
7c
1.00
1.02
1.00
1.87
0.91
1.98
1.98
5.98
9.0
8.5
8.0
7.5
7.0
6.5
6.0
5.5
5.0
4.5
4.0
3.5
3.0
2.5
2.0
1.5
1.0
0.5
0.0
f1 (ppm)

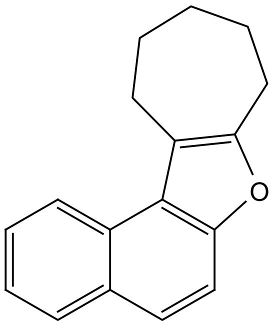

7c

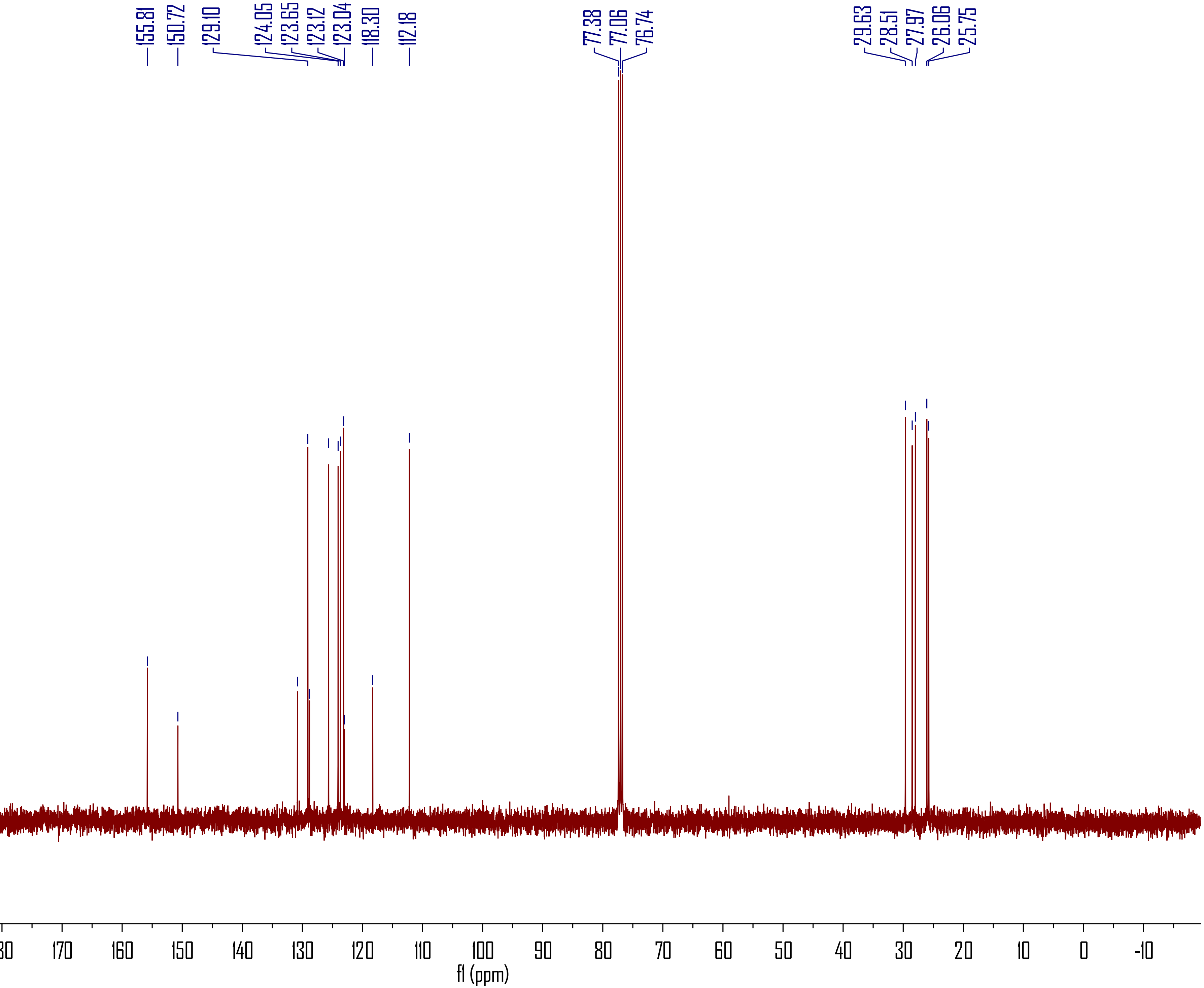
155.81
150.72
129.10
124.05
123.65
123.12
123.04
118.30
112.18
77.38
77.06
76.74
29.63
28.51
27.97
26.06
25.75
210 200 190 180 170 160 150 140 130 120 110 100 90 80 70 60 50 40 30 20 10 0 -10
f1 (ppm)

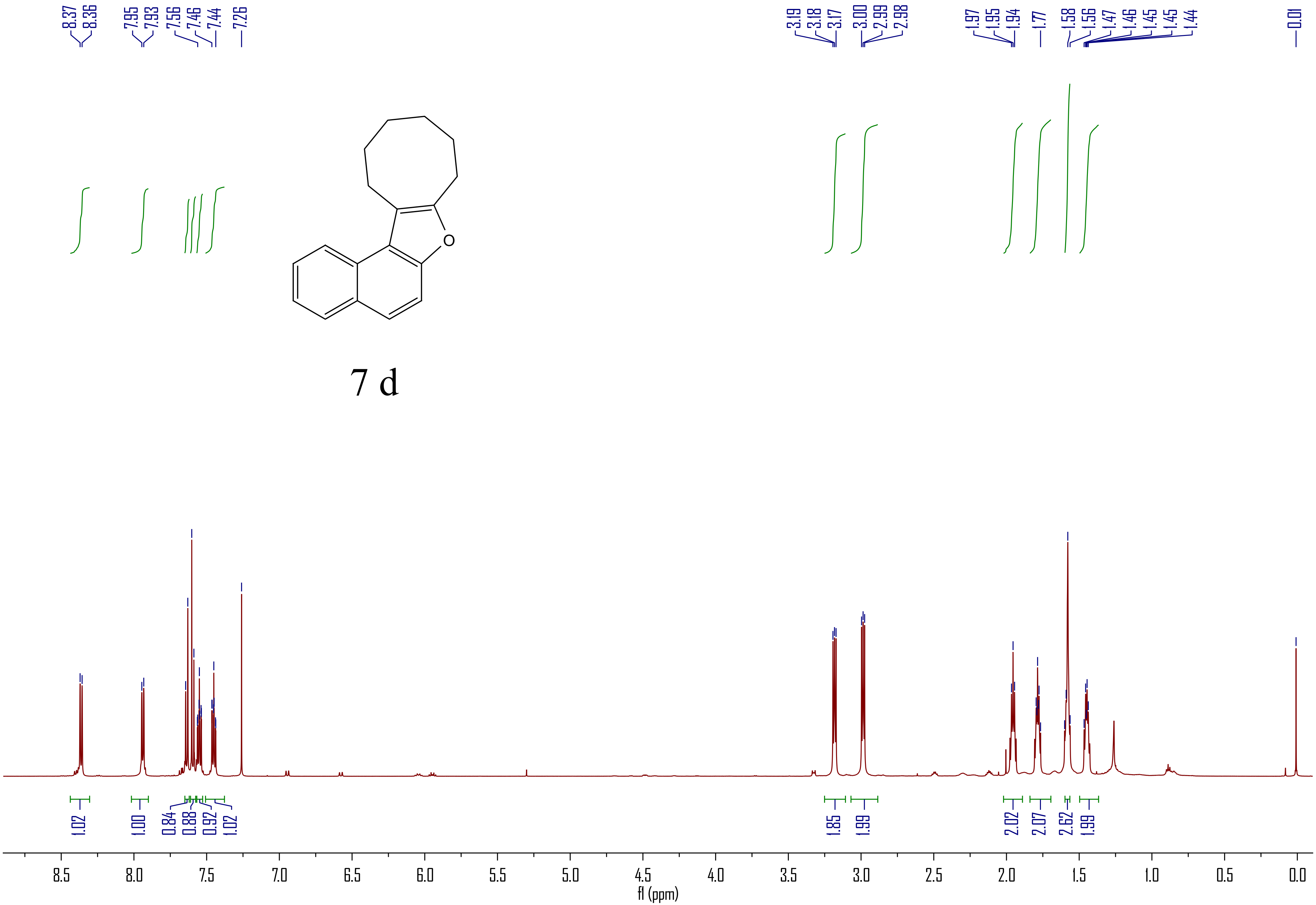
8.37
8.36
7.95
7.93
7.56
7.46
7.44
7.26
3.19
3.18
3.17
3.00
2.99
2.98
1.97
1.95
1.94
1.77
1.58
1.56
1.47
1.46
1.45
1.45
1.44
0.01
O
7 d
1.02
1.00
0.84
0.88
0.92
1.02
1.85
1.99
2.02
2.07
2.62
1.99
8.5
8.0
7.5
7.0
6.5
6.0
5.5
5.0
4.5
4.0
3.5
3.0
2.5
2.0
1.5
1.0
0.5
0.0
fl (ppm)

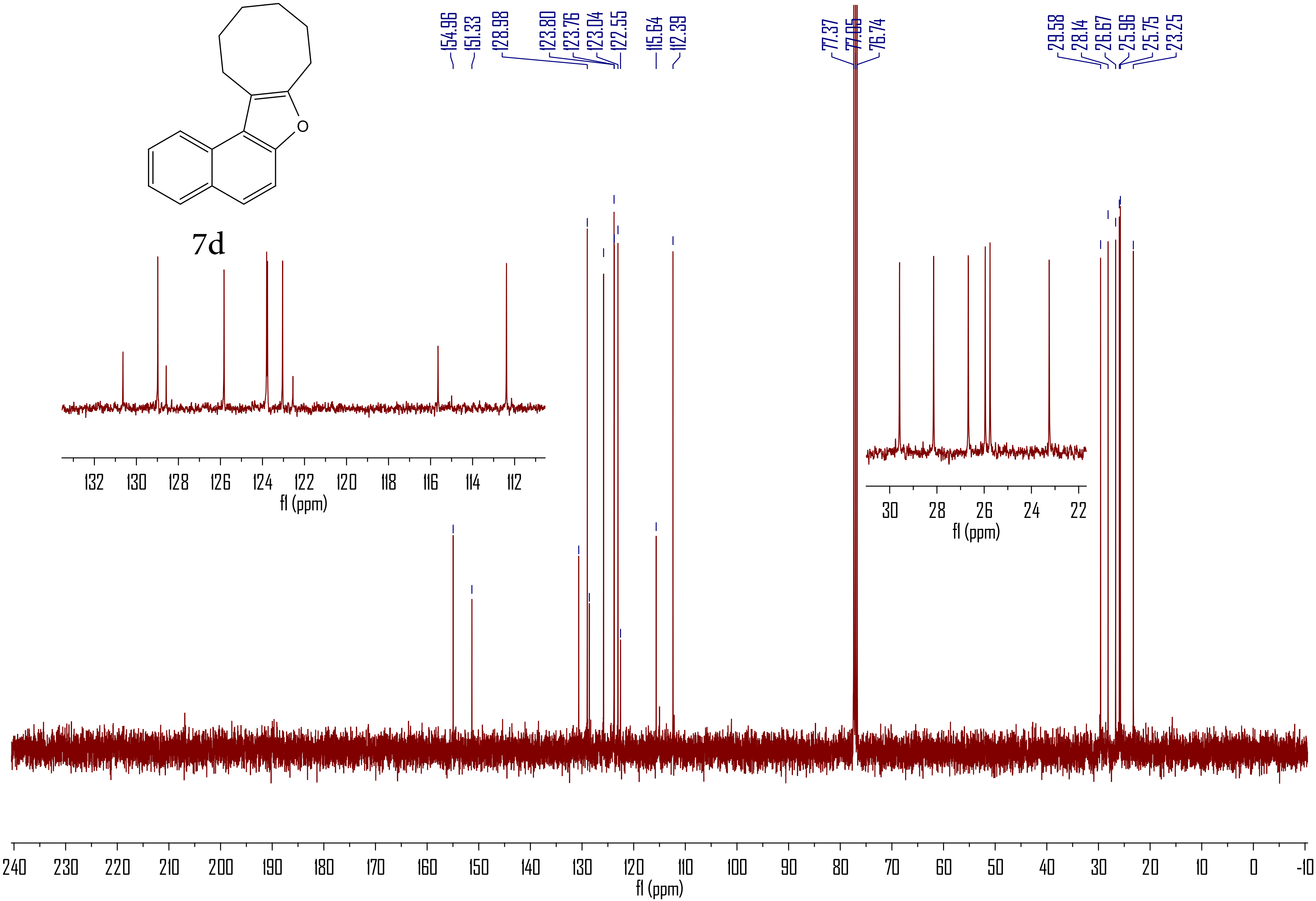
154.96
151.33
128.98
123.80
123.76
123.04
122.55
115.64
112.39
77.37
77.05
76.74
29.58
28.14
26.67
25.96
25.75
23.25
7d
fl (ppm)
fl (ppm)
fl (ppm)

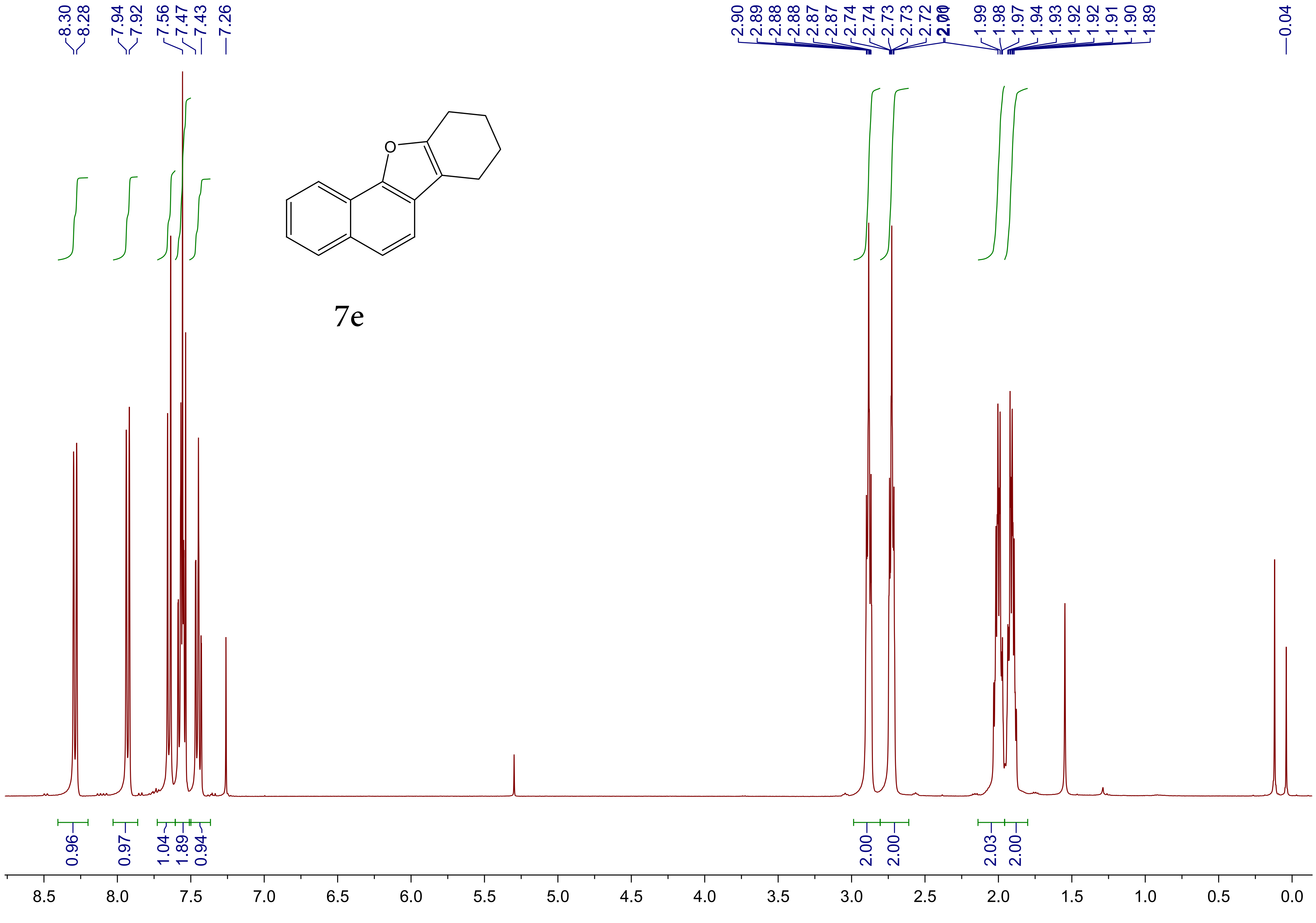
8.30
8.28
7.94
7.92
7.56
7.47
7.43
7.26
2.90
2.89
2.88
2.88
2.87
2.87
2.74
2.74
2.73
2.73
2.72
1.99
1.98
1.97
1.94
1.93
1.92
1.92
1.91
1.90
1.89
0.04
O
7e
0.96
0.97
1.04
1.89
0.94
2.00
2.00
2.03
2.00
8.5
8.0
7.5
7.0
6.5
6.0
5.5
5.0
4.5
4.0
3.5
3.0
2.5
2.0
1.5
1.0
0.5
0.0

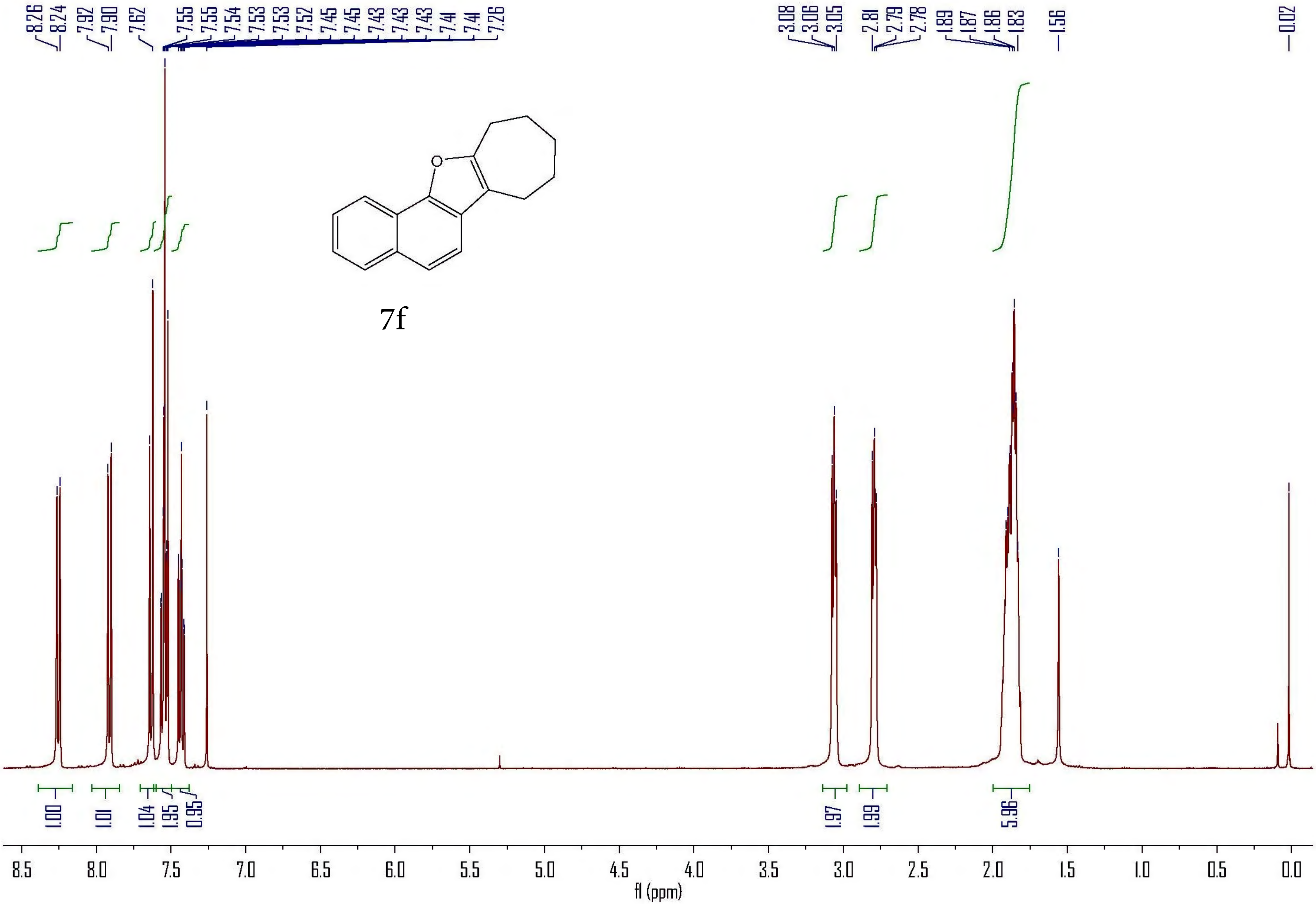
8.26
8.24
7.92
7.90
7.62
7.55
7.55
7.54
7.53
7.53
7.52
7.45
7.45
7.43
7.43
7.43
7.41
7.41
7.26
3.08
3.06
3.05
2.81
2.79
2.78
1.89
1.87
1.86
1.83
1.56
0.02
7f
1.00
1.01
1.04
1.95
0.95
1.97
1.99
5.96
8.5
8.0
7.5
7.0
6.5
6.0
5.5
5.0
4.5
4.0
3.5
3.0
2.5
2.0
1.5
1.0
0.5
0.0
f1 (ppm)

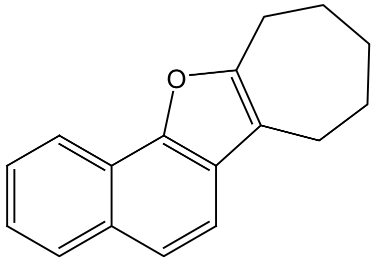
O

7f

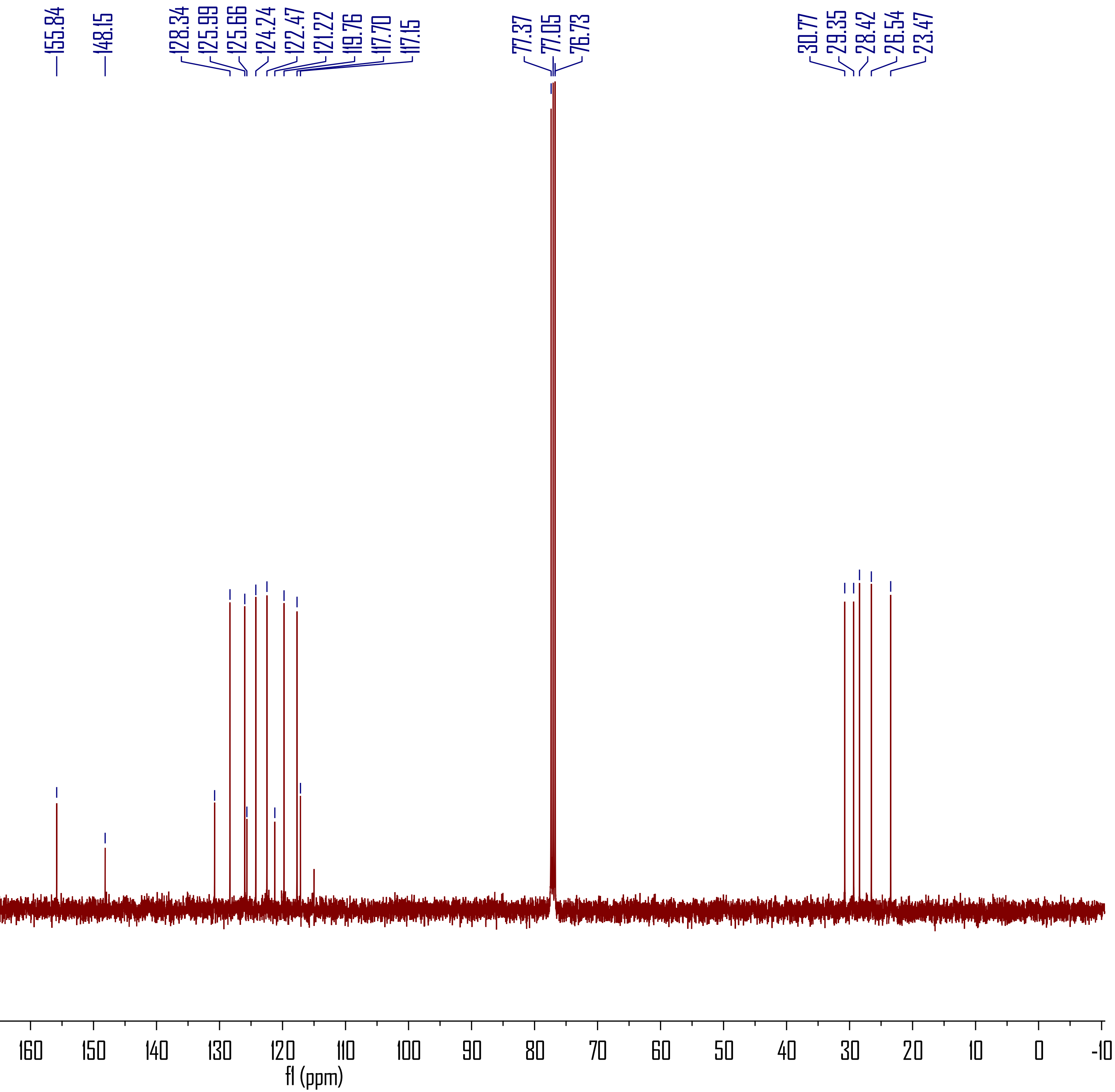
155.84
148.15
128.34
125.99
125.66
124.24
122.47
121.22
119.76
117.70
117.15
77.37
77.05
76.73
30.77
29.35
28.42
26.54
23.47
240 230 220 210 200 190 180 170 160 150 140 130 120 110 100 90 80 70 60 50 40 30 20 10 0 -10
f1 (ppm)

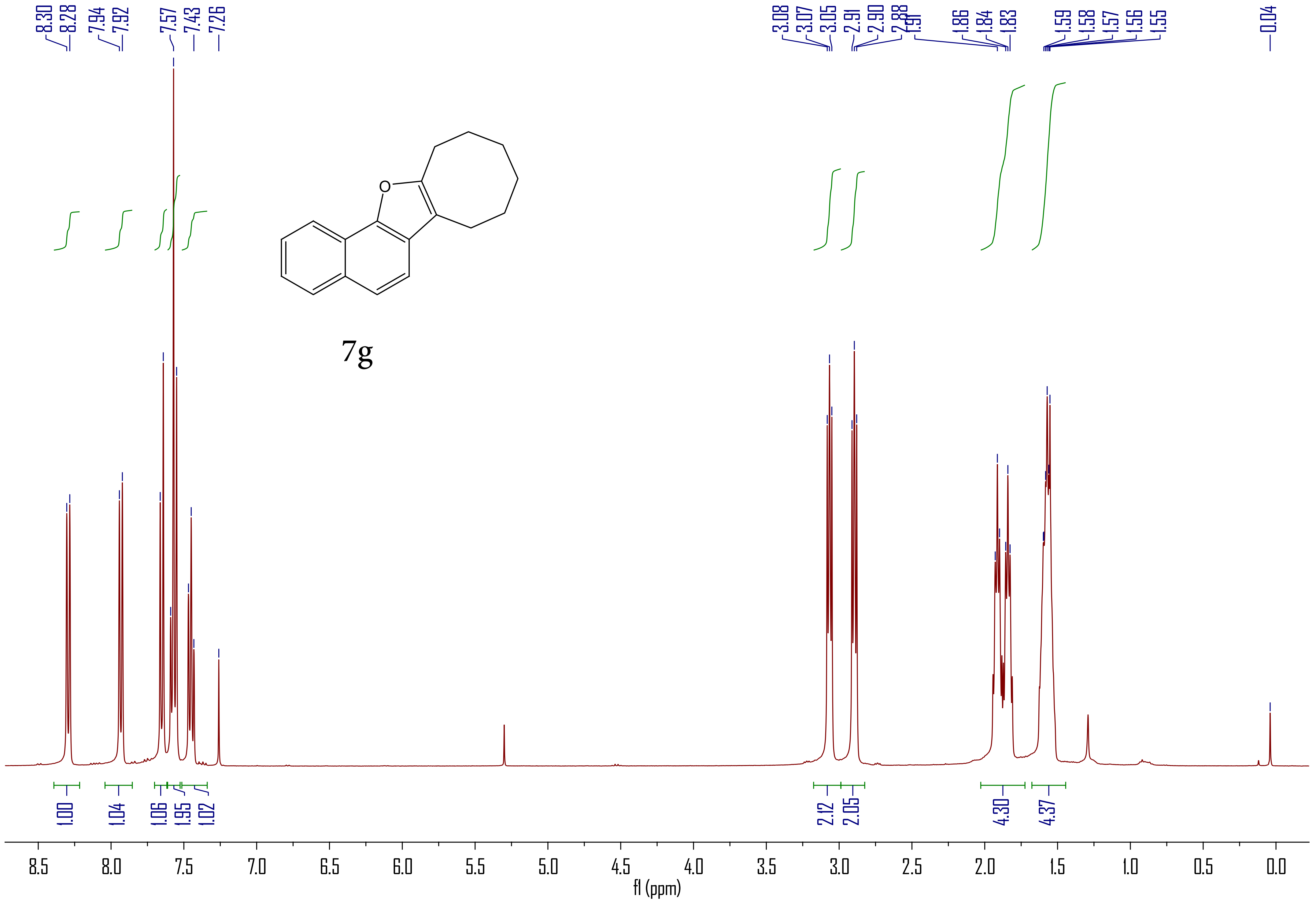

8.30
8.28
7.94
7.92
7.57
7.43
7.26
3.08
3.07
3.05
2.91
2.90
2.88
1.91
1.86
1.84
1.83
1.59
1.58
1.57
1.56
1.55
0.04
O
7g
1.00
1.04
1.06
1.95
1.02
2.12
2.05
4.30
4.37
8.5
8.0
7.5
7.0
6.5
6.0
5.5
5.0
4.5
4.0
3.5
3.0
2.5
2.0
1.5
1.0
0.5
0.0
f1 (ppm)

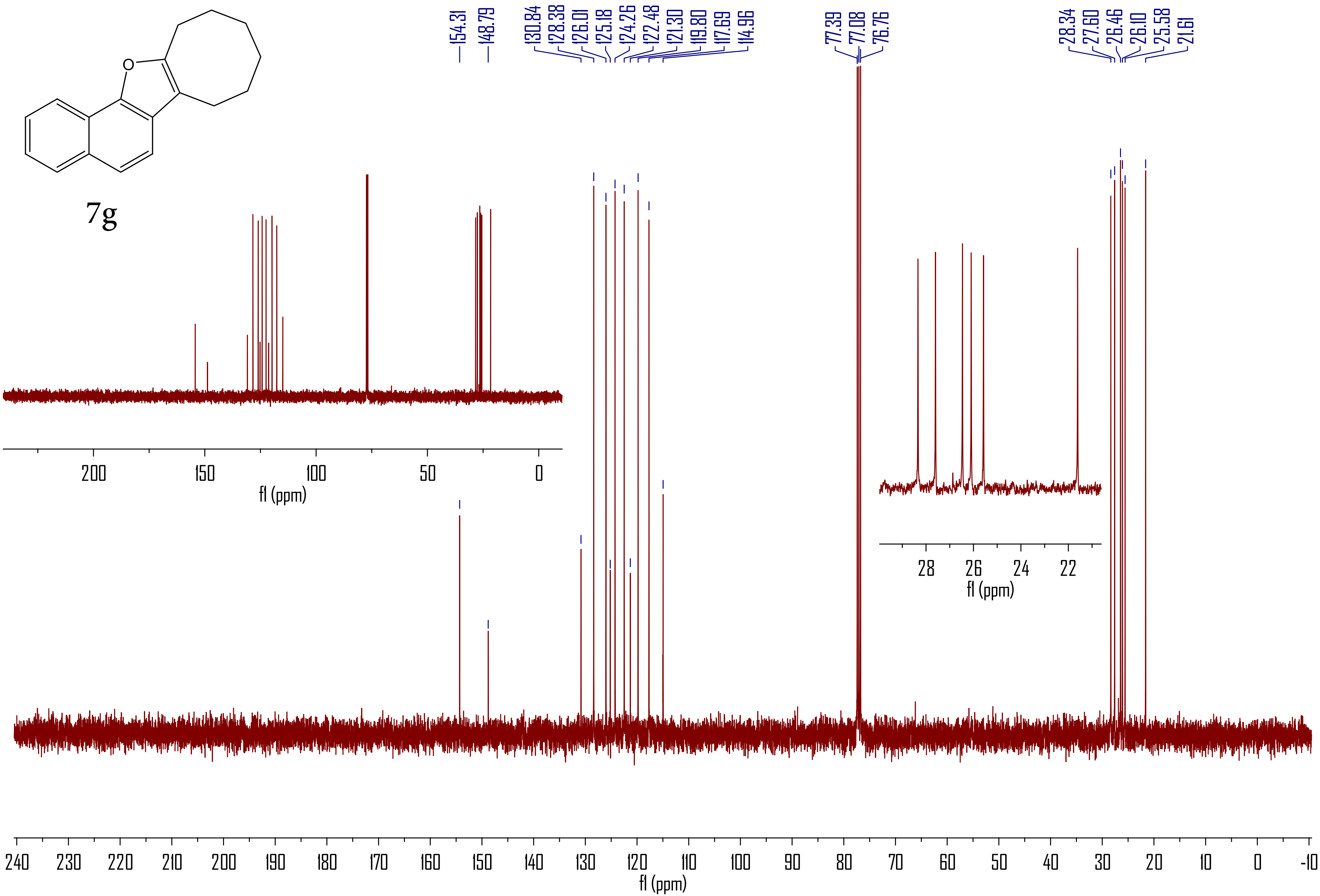

7g
154.31
148.79
130.84
128.38
126.01
125.18
124.26
122.48
121.30
119.80
117.69
114.96
77.39
77.08
76.76
28.34
27.60
26.46
26.10
25.58
21.61
200 150 100 50 0
f1 (ppm)
28 26 24 22
f1 (ppm)
240 230 220 210 200 190 180 170 160 150 140 130 120 110 100 90 80 70 60 50 40 30 20 10 0 -10
f1 (ppm)

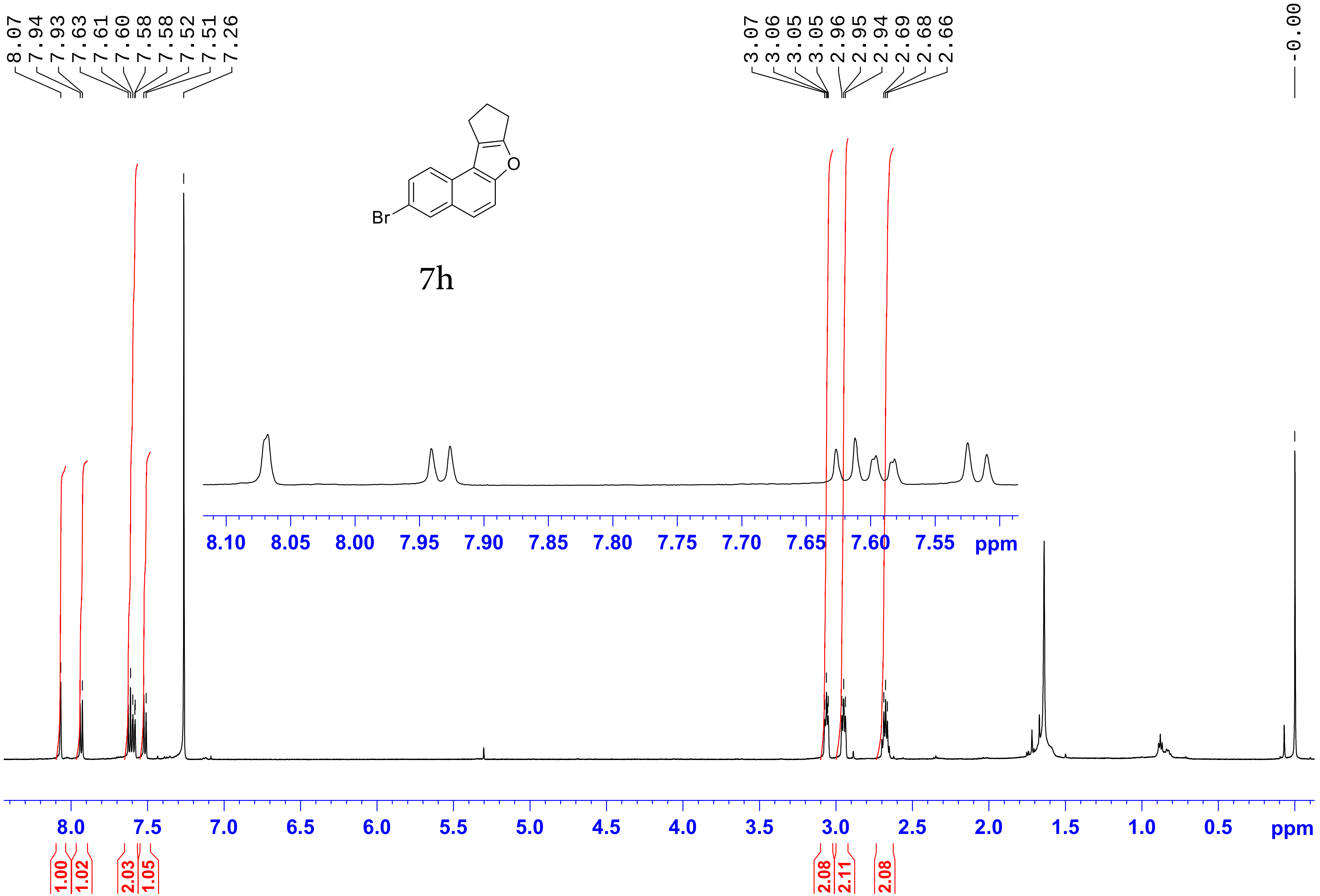
8.07
7.94
7.93
7.63
7.61
7.60
7.58
7.58
7.52
7.51
7.26
3.07
3.06
3.05
3.05
2.96
2.95
2.94
2.69
2.68
2.66
-0.00
Br
O
7h
8.10
8.05
8.00
7.95
7.90
7.85
7.80
7.75
7.70
7.65
7.60
7.55
ppm
8.0
7.5
7.0
6.5
6.0
5.5
5.0
4.5
4.0
3.5
3.0
2.5
2.0
1.5
1.0
0.5
ppm
1.00
1.02
2.03
1.05
2.08
2.11
2.08

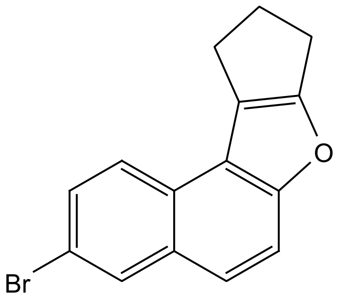
O
Br

7h

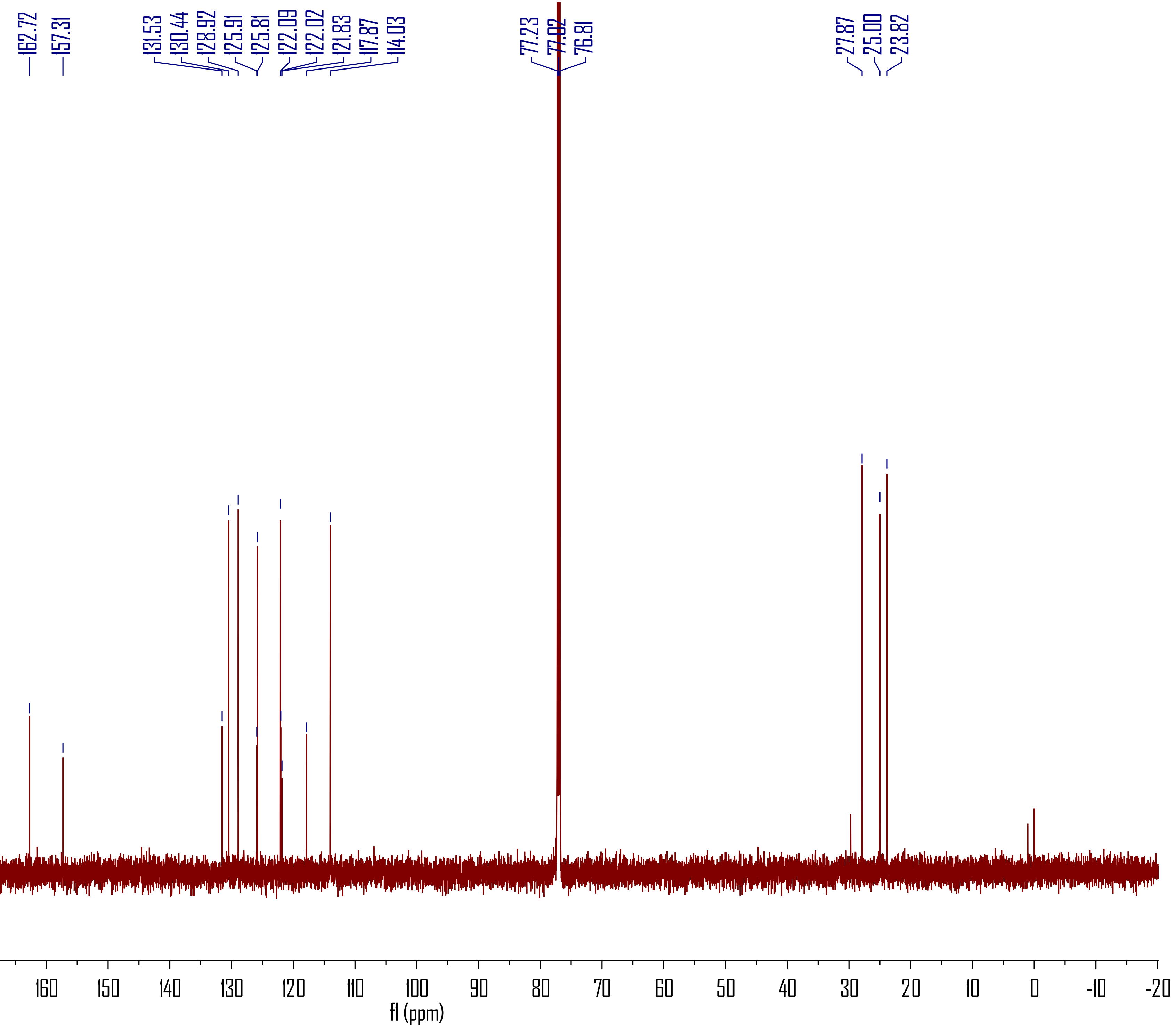
162.72
157.31
131.53
130.44
128.92
125.91
125.81
122.09
122.02
121.83
117.87
114.03
77.23
77.02
76.81
27.87
25.00
23.82
220 210 200 190 180 170 160 150 140 130 120 110 100 90 80 70 60 50 40 30 20 10 0 -10 -20
f1 (ppm)

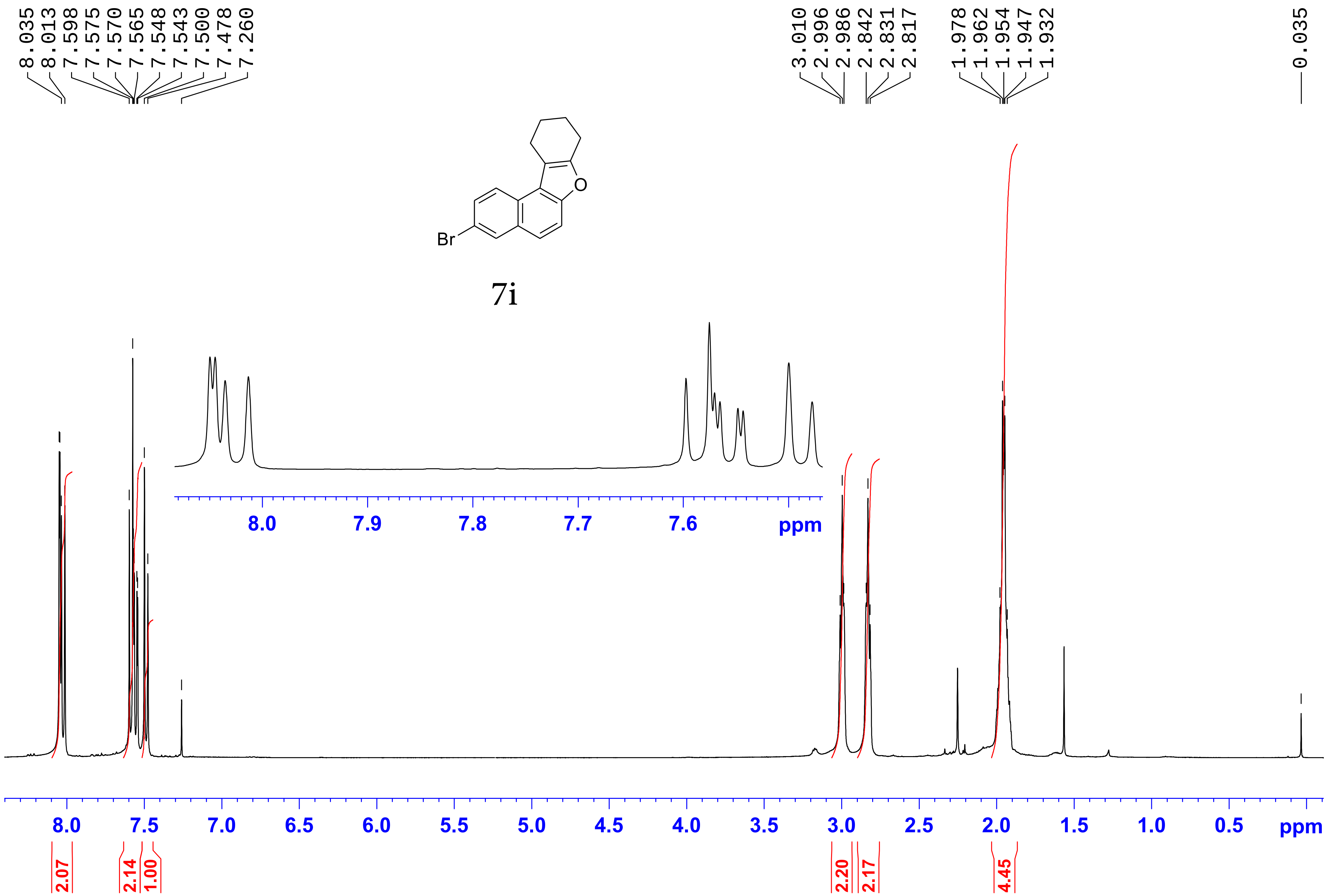

8.035
8.013
7.598
7.575
7.570
7.565
7.548
7.543
7.500
7.478
7.260
3.010
2.996
2.986
2.842
2.831
2.817
1.978
1.962
1.954
1.947
1.932
0.035
Br
O
7i
8.0
7.9
7.8
7.7
7.6
ppm
8.0
7.5
7.0
6.5
6.0
5.5
5.0
4.5
4.0
3.5
3.0
2.5
2.0
1.5
1.0
0.5
ppm
2.07
2.14
1.00
2.20
2.17
4.45

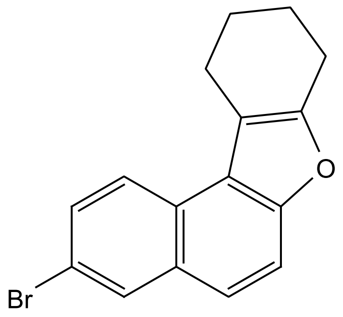
Br
O

7i

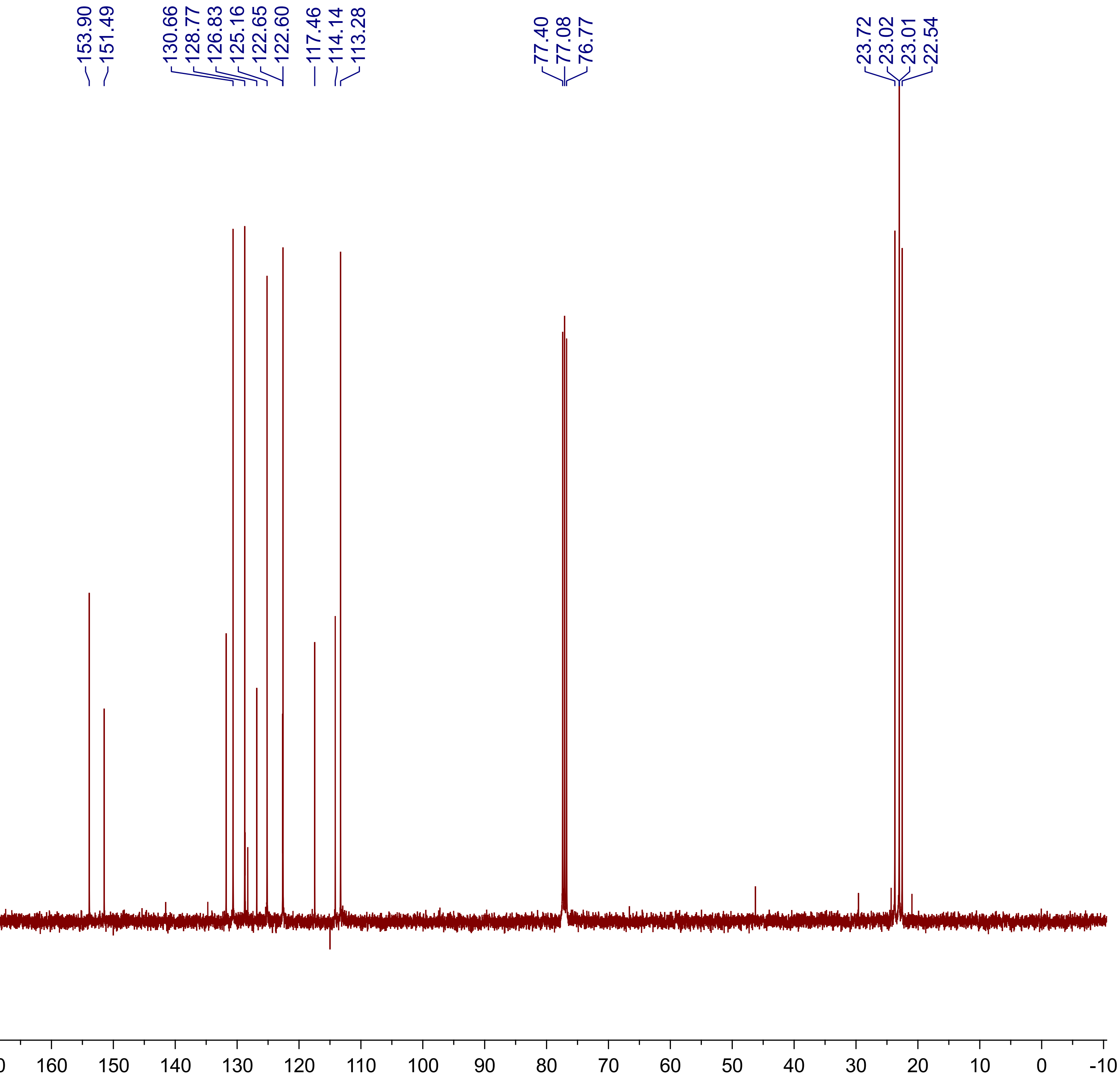
153.90
151.49
130.66
128.77
126.83
125.16
122.65
122.60
117.46
114.14
113.28
77.40
77.08
76.77
23.72
23.02
23.01
22.54
240 230 220 210 200 190 180 170 160 150 140 130 120 110 100 90 80 70 60 50 40 30 20 10 0 -10

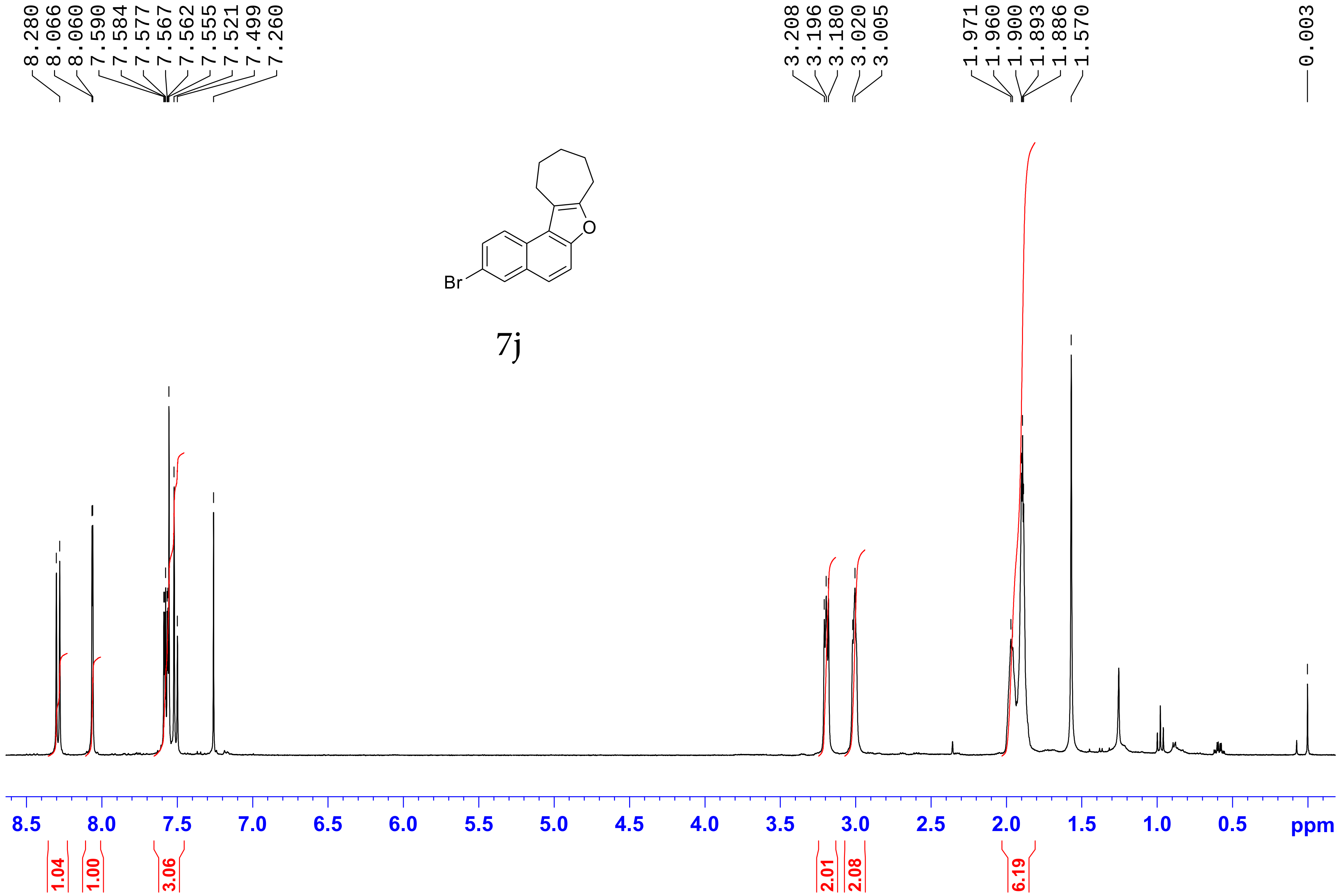

8.280
8.066
8.060
7.590
7.584
7.577
7.567
7.562
7.555
7.521
7.499
7.260
3.208
3.196
3.180
3.020
3.005
1.971
1.960
1.900
1.893
1.886
1.570
0.003
Br
O
7j
8.5
8.0
7.5
7.0
6.5
6.0
5.5
5.0
4.5
4.0
3.5
3.0
2.5
2.0
1.5
1.0
0.5
ppm
1.04
1.00
3.06
2.01
2.08
6.19

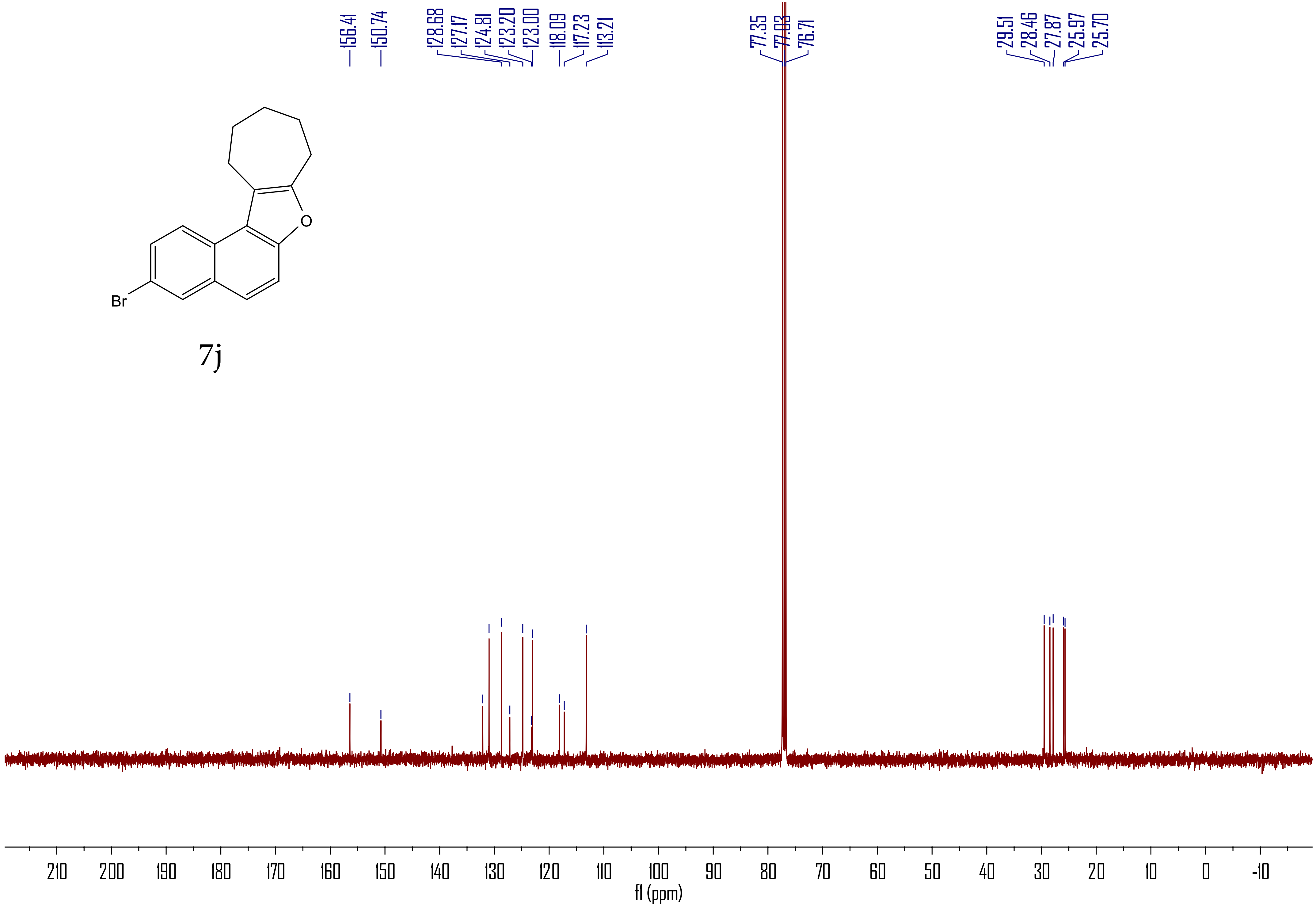
156.41
150.74
128.68
127.17
124.81
123.20
123.00
118.09
117.23
113.21
77.35
77.03
76.71
29.51
28.46
27.87
25.97
25.70
O
Br
7j
210 200 190 180 170 160 150 140 130 120 110 100 90 80 70 60 50 40 30 20 10 0 -10
f1 (ppm)

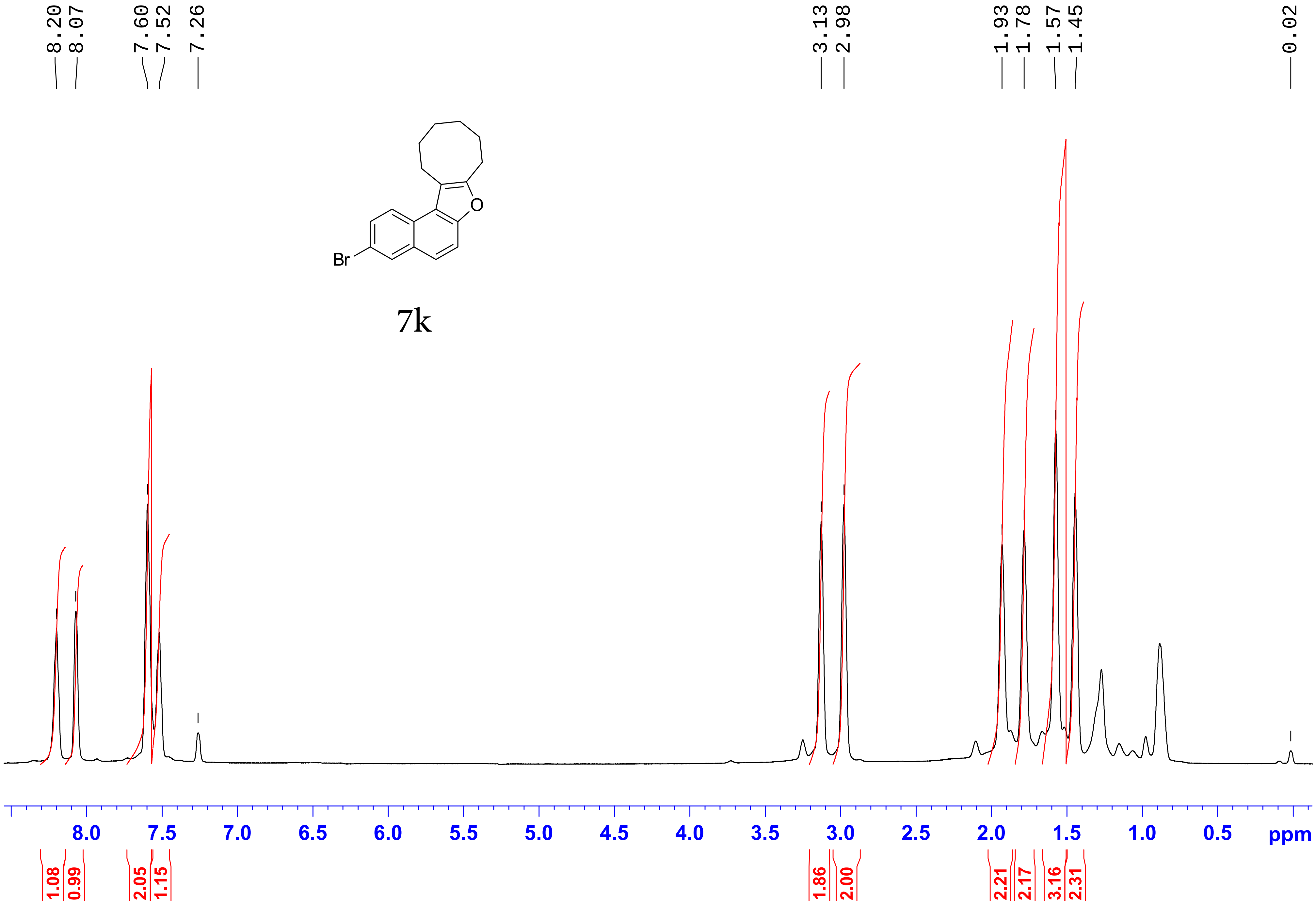
8.20
8.07
7.60
7.52
7.26
3.13
2.98
1.93
1.78
1.57
1.45
0.02
Br
O
7k
8.0
7.5
7.0
6.5
6.0
5.5
5.0
4.5
4.0
3.5
3.0
2.5
2.0
1.5
1.0
0.5
ppm
1.08
0.99
2.05
1.15
1.86
2.00
2.21
2.17
3.16
2.31

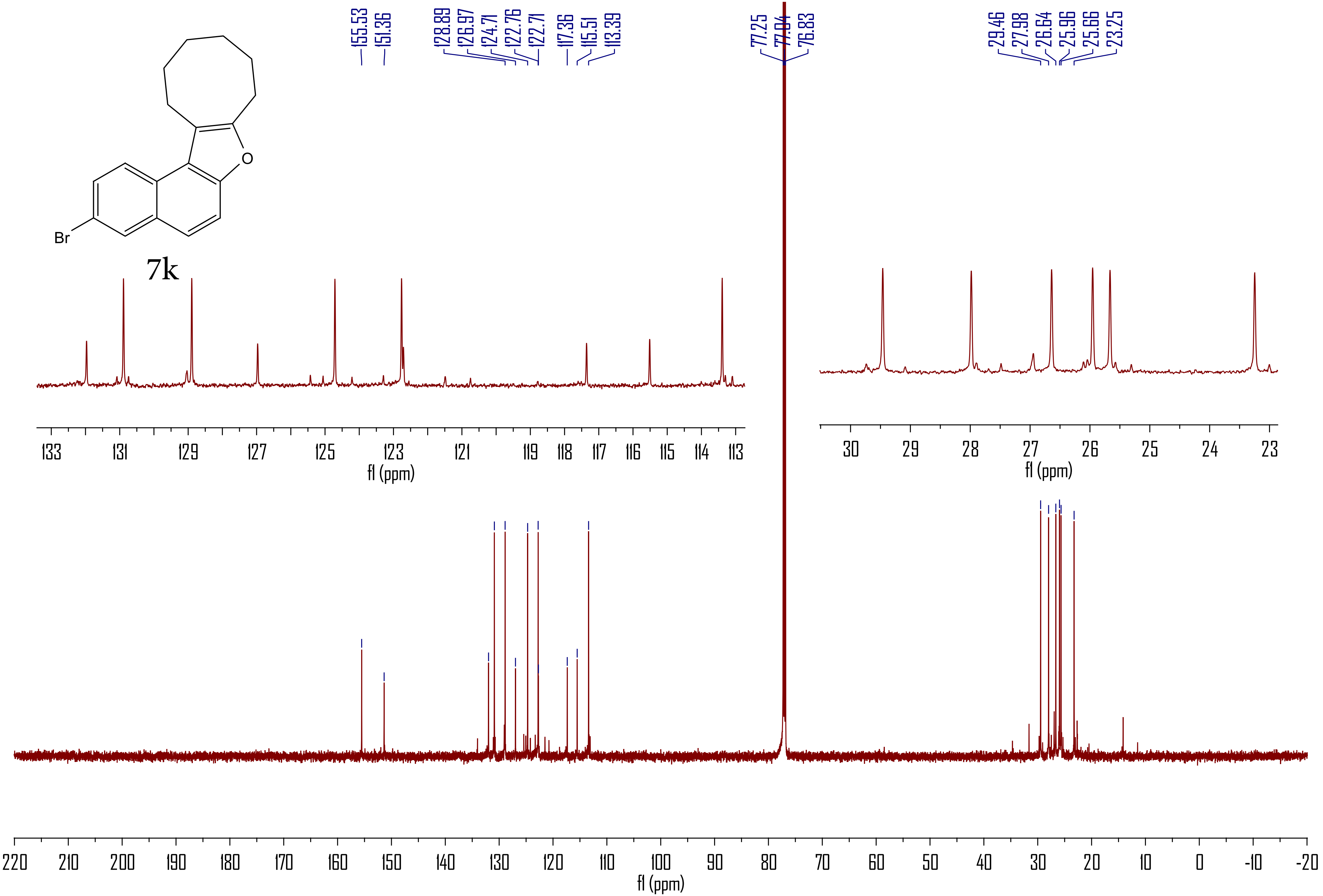
155.53
151.36
128.89
126.97
124.71
122.76
122.71
117.36
115.51
113.39
77.25
77.04
76.83
29.46
27.98
26.64
25.96
25.66
23.25
Br
O
7k
f1 (ppm)
f1 (ppm)
f1 (ppm)

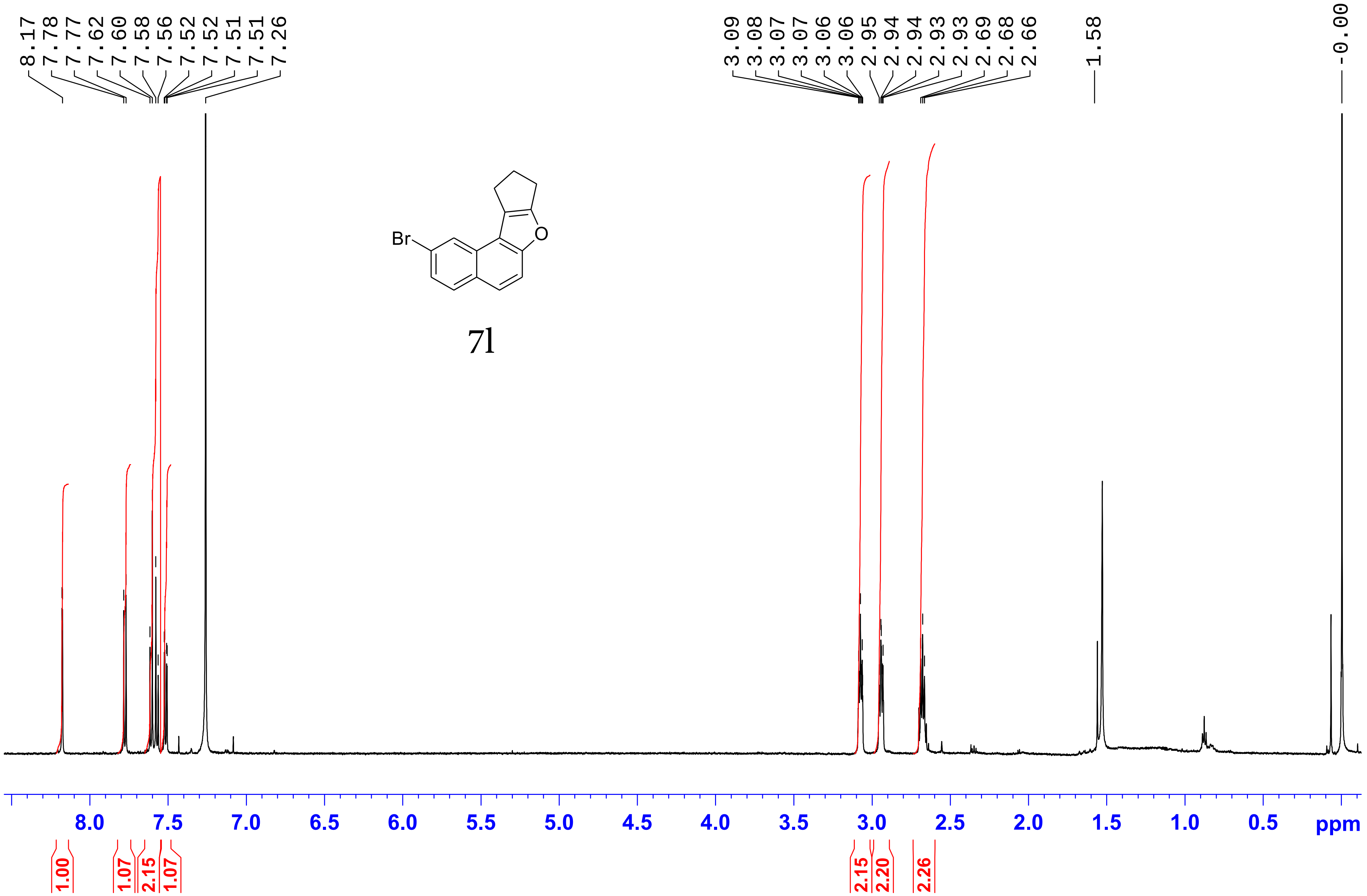
8.17
7.78
7.77
7.62
7.60
7.58
7.56
7.52
7.52
7.51
7.51
7.26
3.09
3.08
3.07
3.07
3.06
3.06
2.95
2.94
2.94
2.93
2.93
2.69
2.68
2.66
1.58
-0.00
Br
O
7l
8.0
7.5
7.0
6.5
6.0
5.5
5.0
4.5
4.0
3.5
3.0
2.5
2.0
1.5
1.0
0.5
ppm
1.00
1.07
2.15
1.07
2.15
2.20
2.26

7l

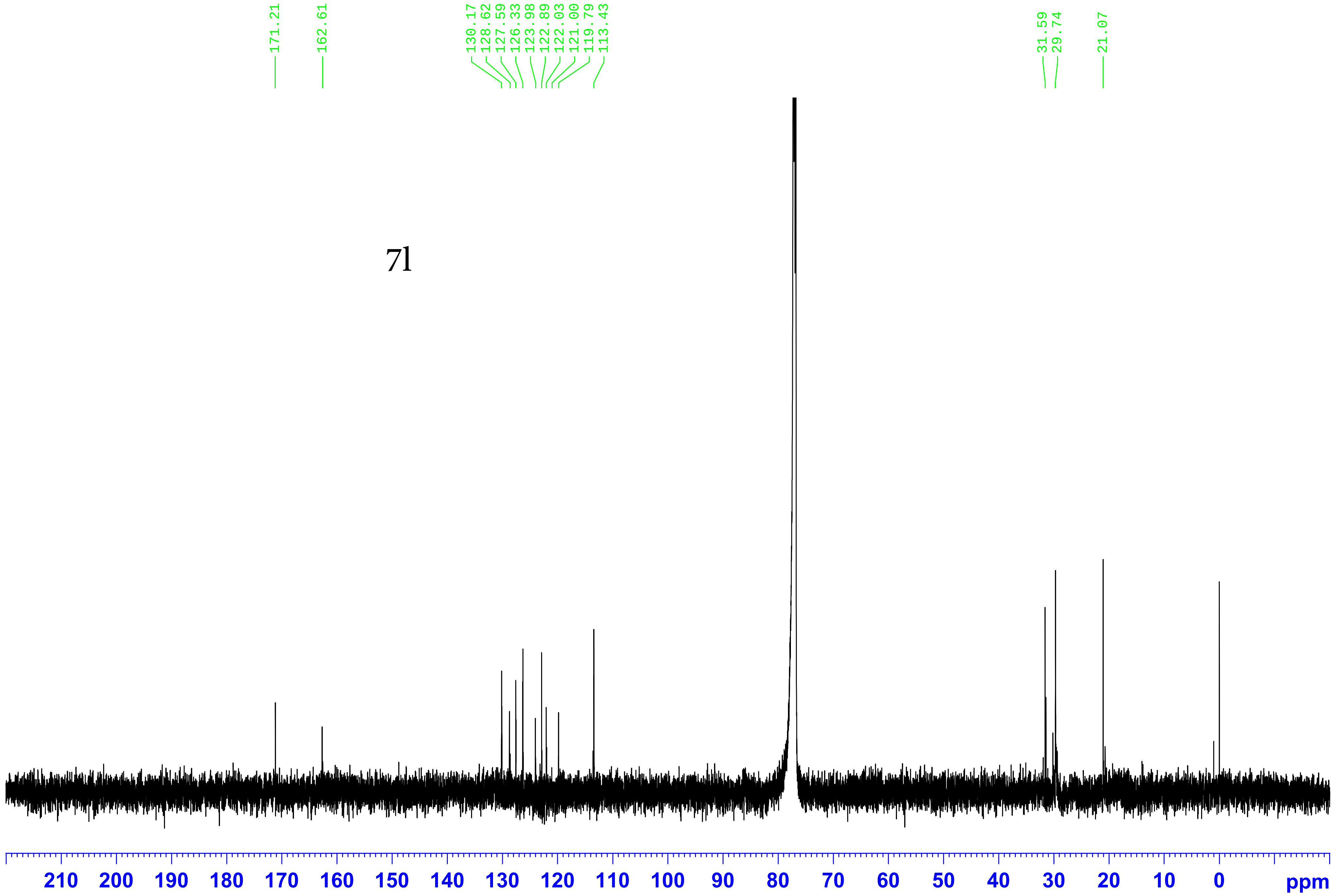

171.21
162.61
130.17
128.62
127.59
126.33
123.98
122.89
122.03
121.00
119.79
113.43
31.59
29.74
21.07
210
200
190
180
170
160
150
140
130
120
110
100
90
80
70
60
50
40
30
20
10
0
ppm

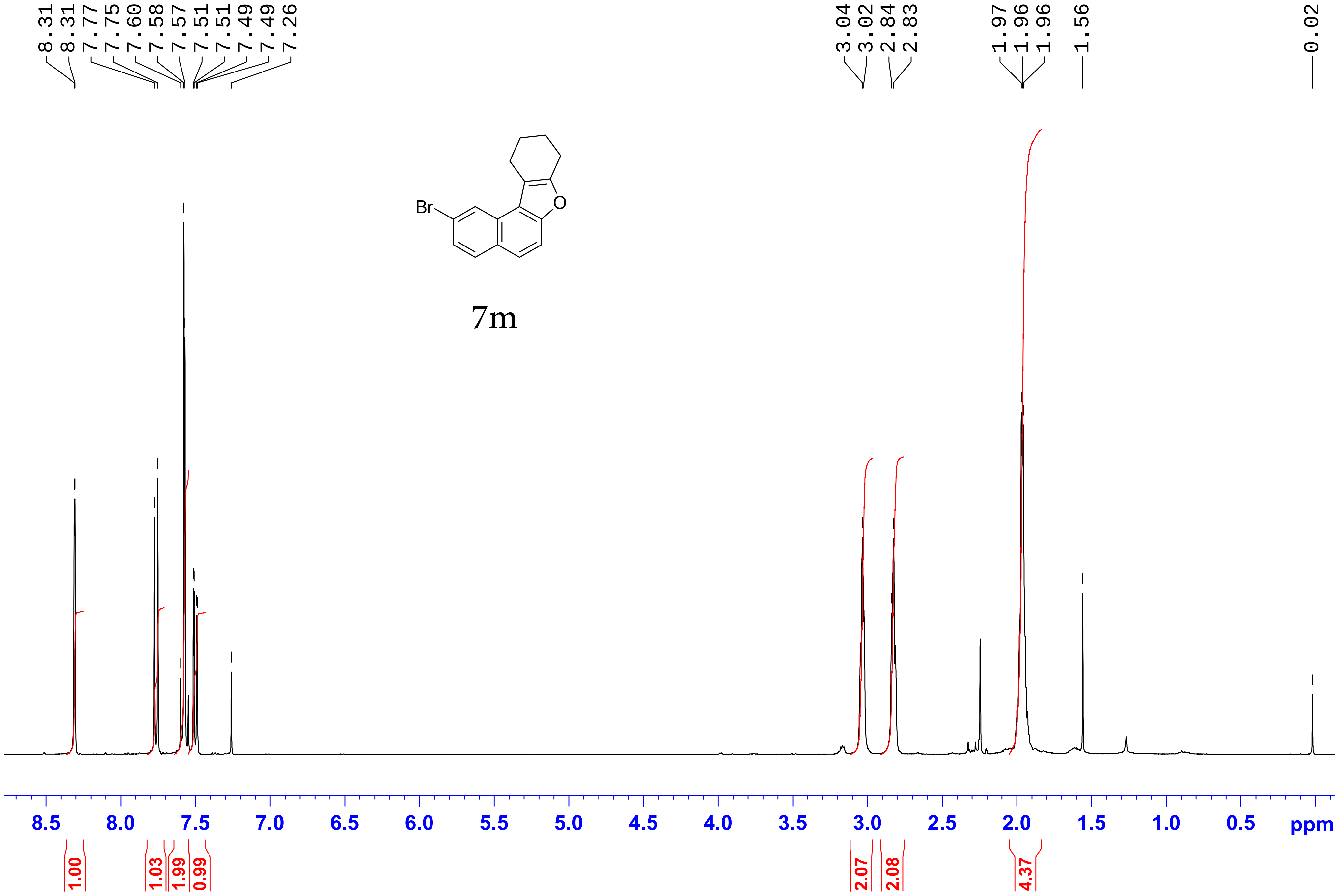

8.31
8.31
7.77
7.75
7.60
7.58
7.57
7.51
7.51
7.49
7.49
7.26
3.04
3.02
2.84
2.83
1.97
1.96
1.96
1.56
0.02
Br
O
7m
8.5
8.0
7.5
7.0
6.5
6.0
5.5
5.0
4.5
4.0
3.5
3.0
2.5
2.0
1.5
1.0
0.5
ppm
1.00
1.03
1.99
0.99
2.07
2.08
4.37

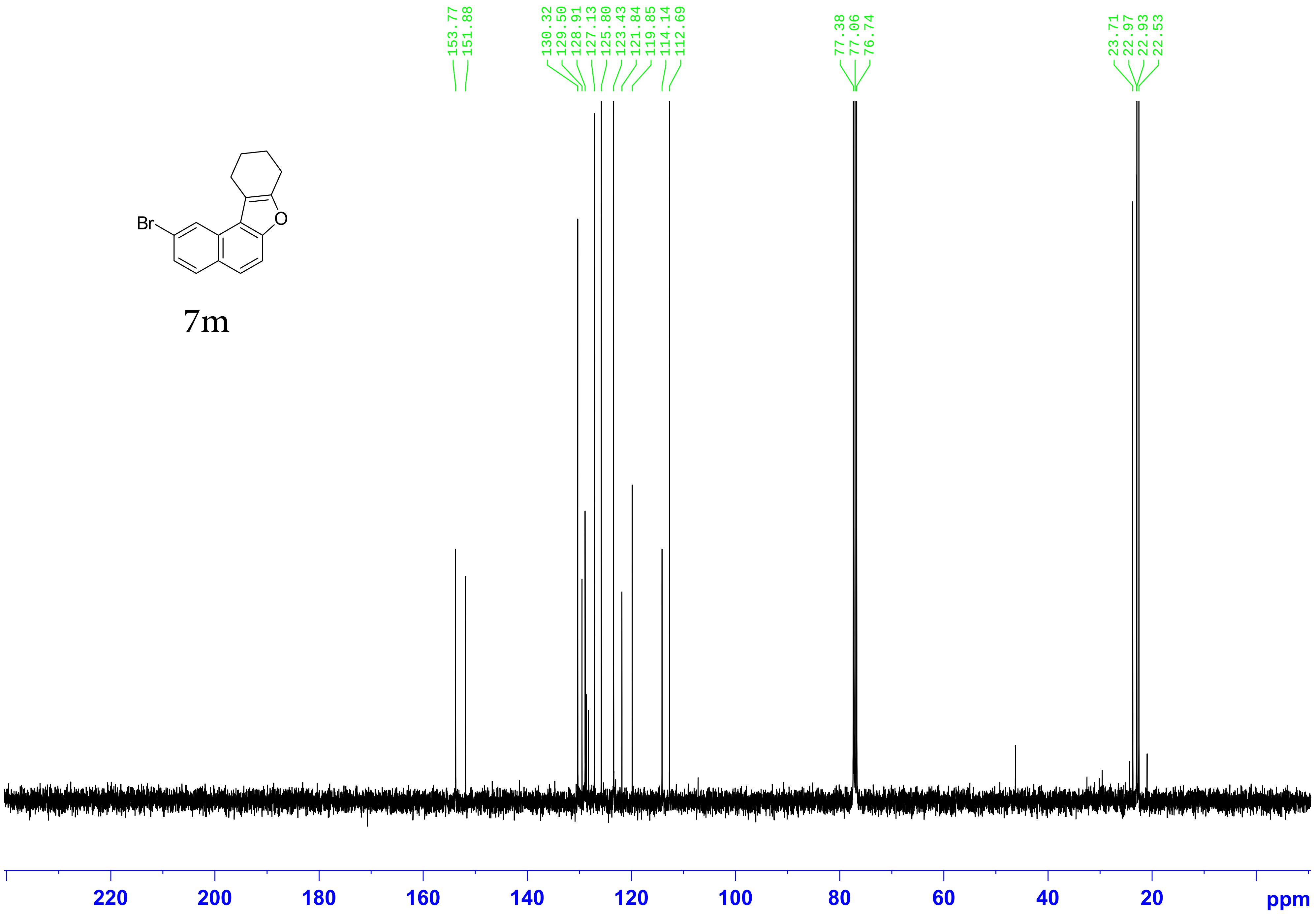
153.77
151.88
130.32
129.50
128.91
127.13
125.80
123.43
121.84
119.85
114.14
112.69
77.38
77.06
76.74
23.71
22.97
22.93
22.53
220
200
180
160
140
120
100
80
60
40
20
ppm

7m

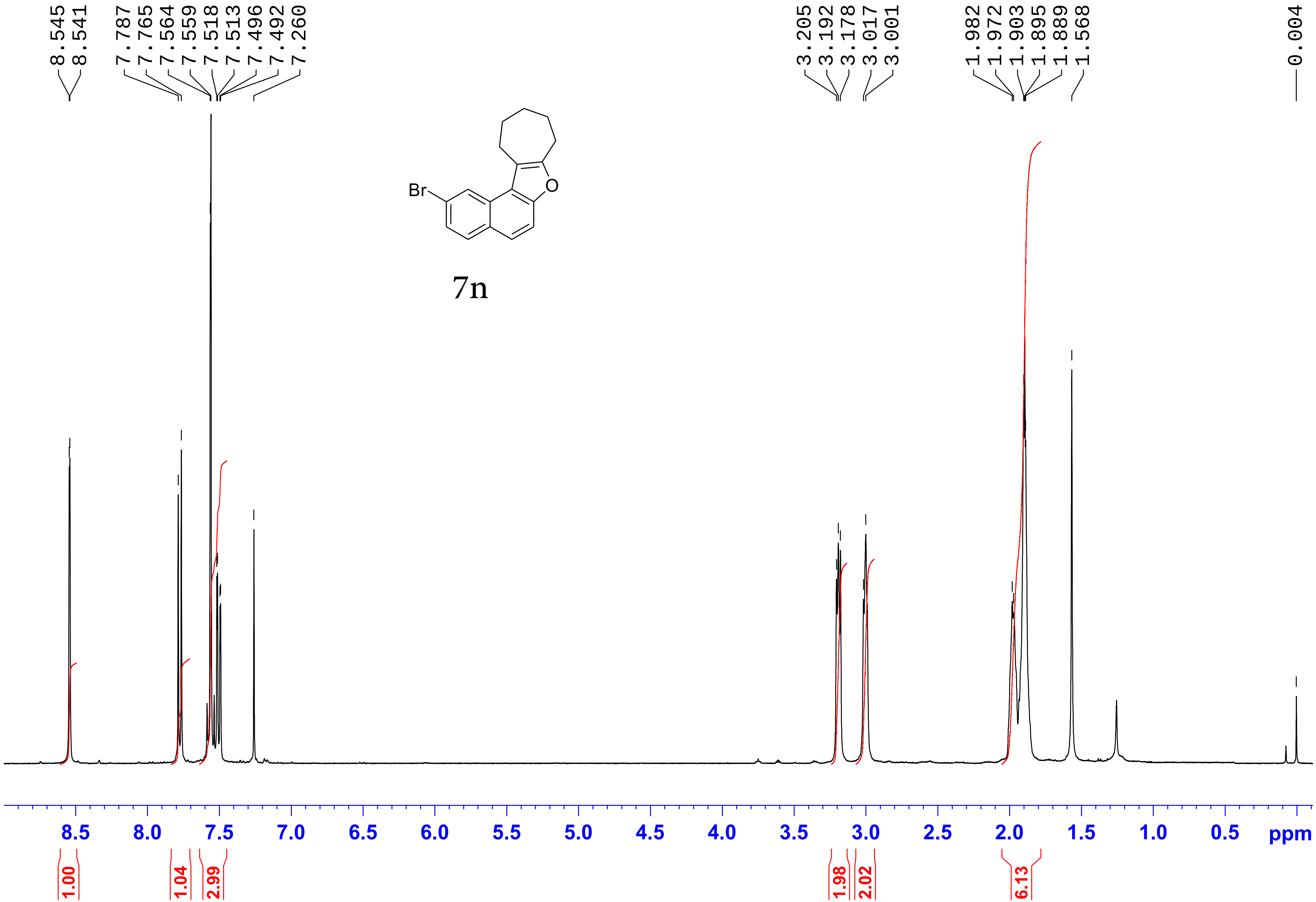
8.545
8.541
7.787
7.765
7.564
7.559
7.518
7.513
7.496
7.492
7.260
3.205
3.192
3.178
3.017
3.001
1.982
1.972
1.903
1.895
1.889
1.568
0.004
Br
O
7n
8.5
8.0
7.5
7.0
6.5
6.0
5.5
5.0
4.5
4.0
3.5
3.0
2.5
2.0
1.5
1.0
0.5
ppm
1.00
1.04
2.99
1.98
2.02
6.13

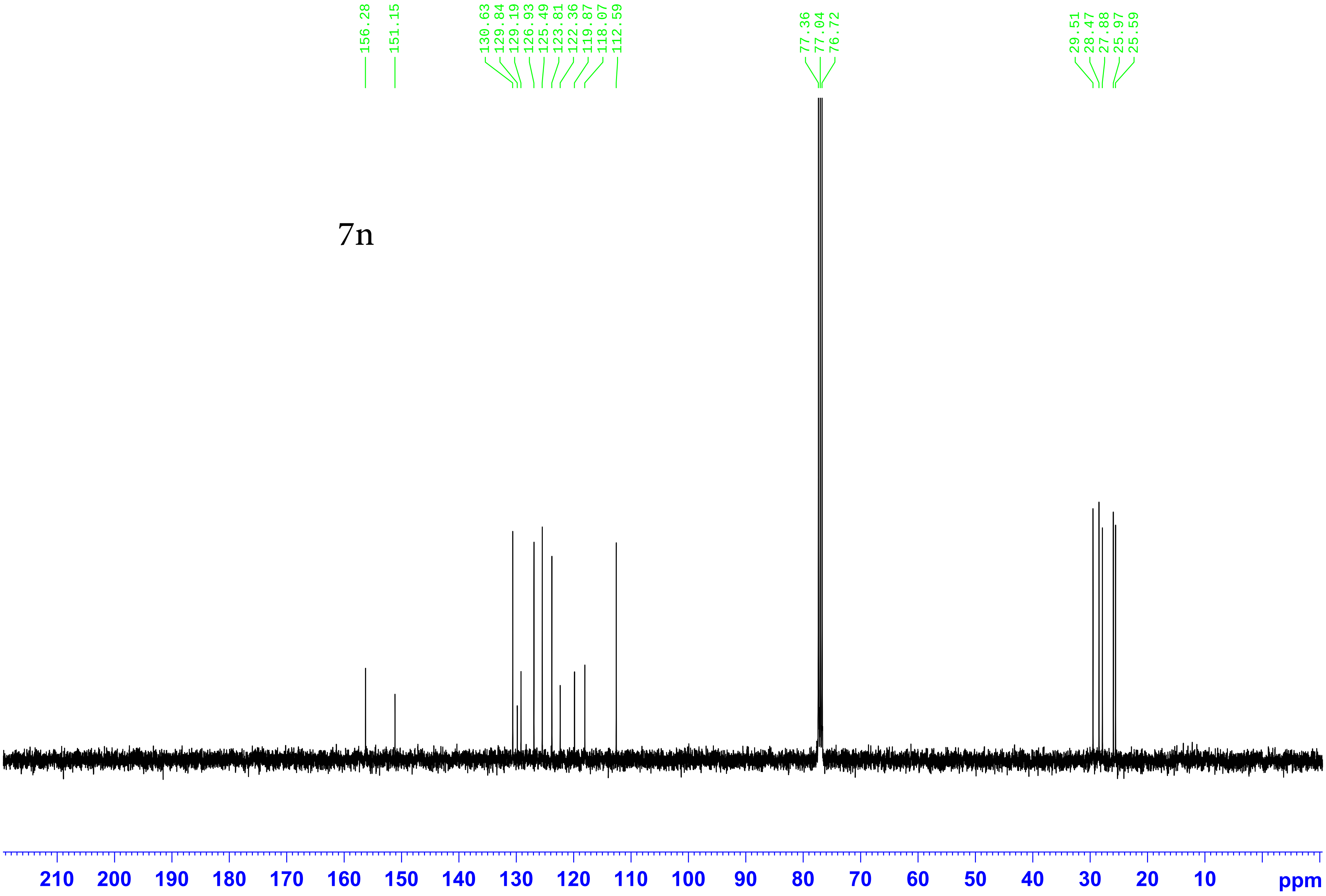
156.28
151.15
130.63
129.84
129.19
126.93
125.49
123.81
122.36
119.87
118.07
112.59
77.36
77.04
76.72
29.51
28.47
27.88
25.97
25.59
7n
210 200 190 180 170 160 150 140 130 120 110 100 90 80 70 60 50 40 30 20 10
ppm

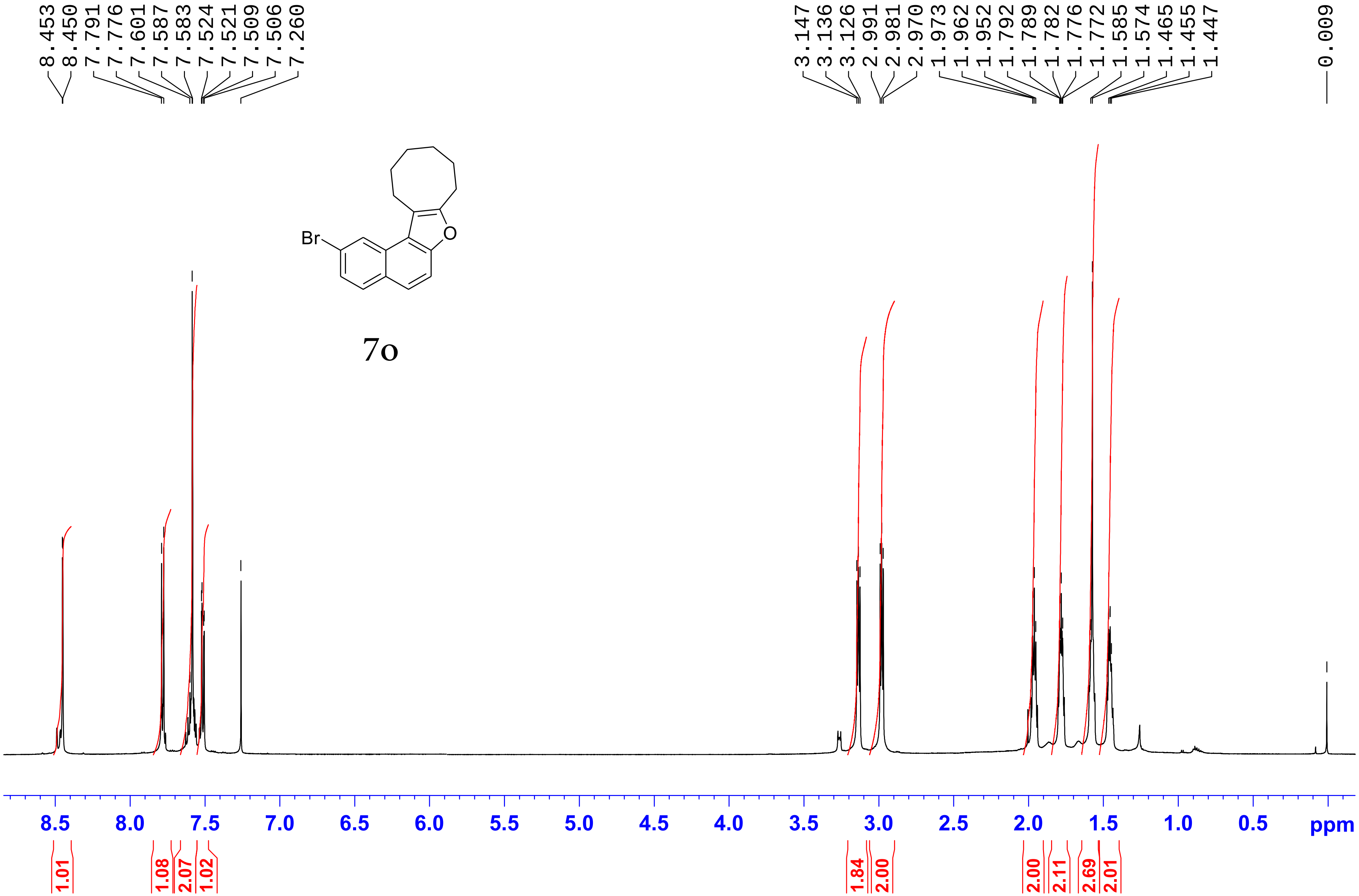

8.453
8.450
7.791
7.776
7.601
7.587
7.583
7.524
7.521
7.509
7.506
7.260
3.147
3.136
3.126
2.991
2.981
2.970
1.973
1.962
1.952
1.792
1.789
1.782
1.776
1.772
1.585
1.574
1.465
1.455
1.447
0.009
Br
O
8.5
8.0
7.5
7.0
6.5
6.0
5.5
5.0
4.5
4.0
3.5
3.0
2.5
2.0
1.5
1.0
0.5
ppm
1.01
1.08
2.07
1.02
1.84
2.00
2.00
2.11
2.69
2.01

7o

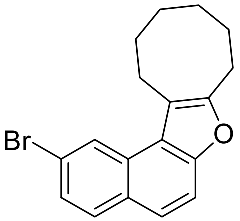
Br
O

7o

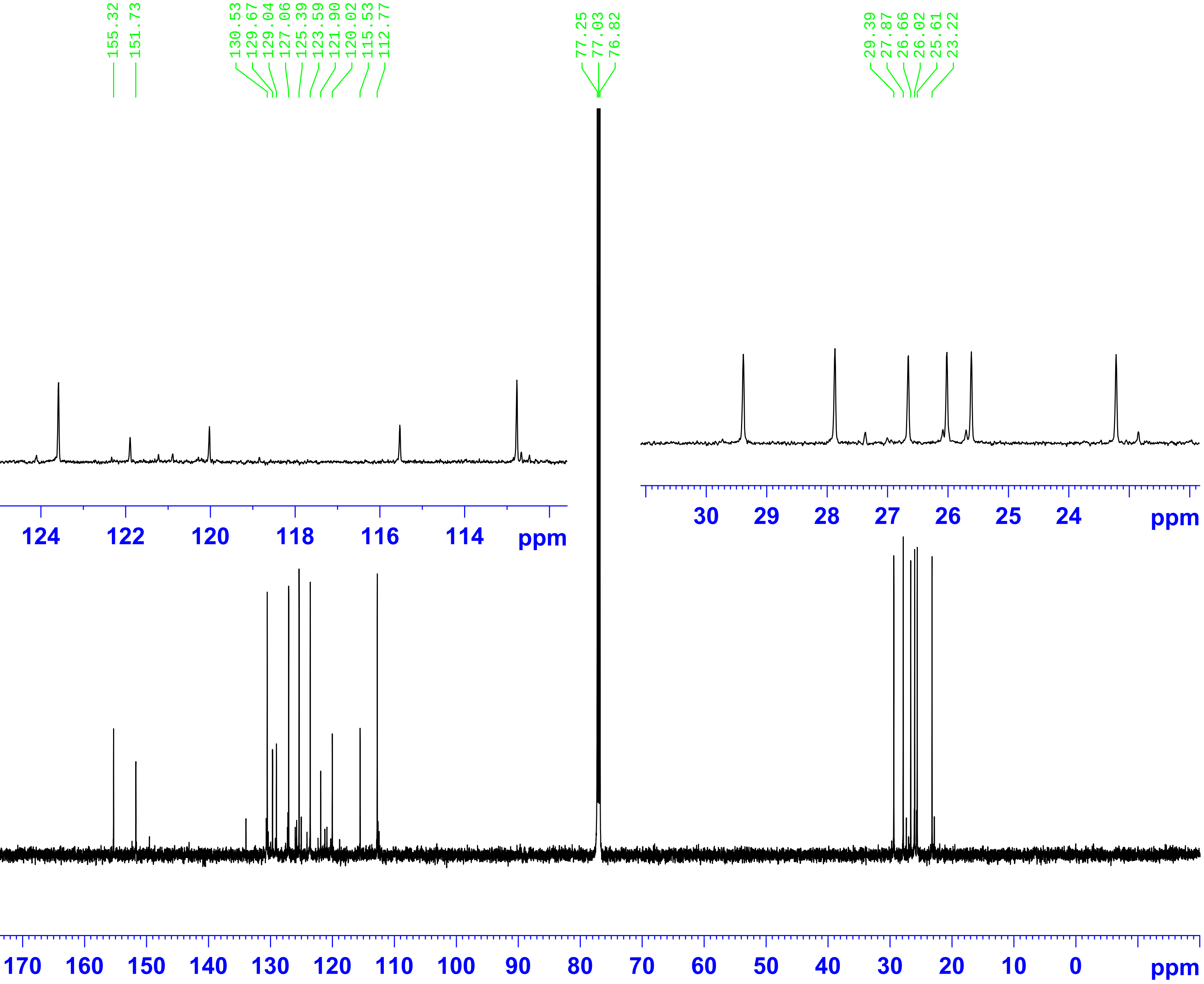
155.32
151.73
130.53
129.67
129.04
127.06
125.39
123.59
121.90
120.02
115.53
112.77
77.25
77.03
76.82
29.39
27.87
26.66
26.02
25.61
23.22
ppm
ppm
ppm

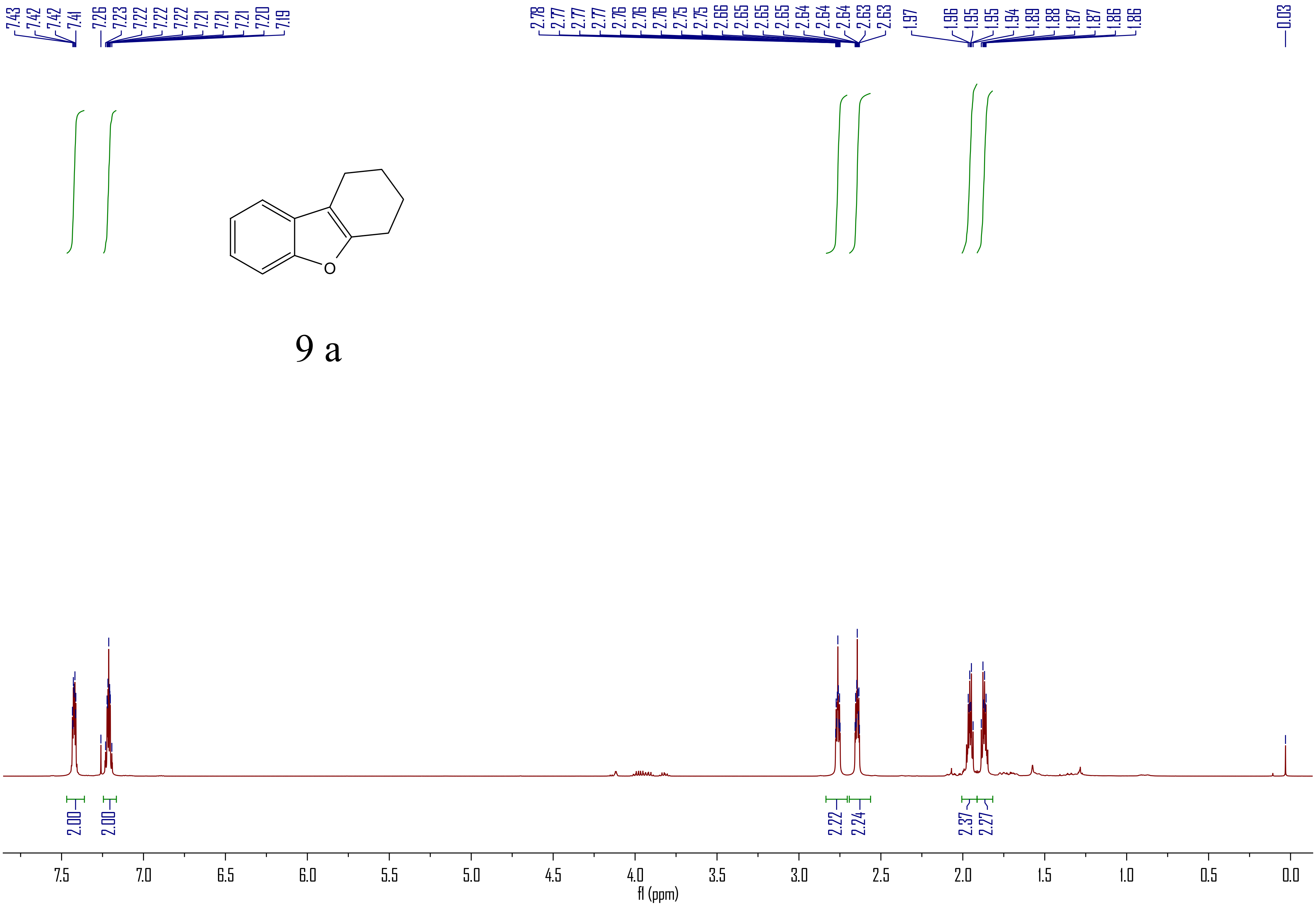
7.43
7.42
7.42
7.41
7.26
7.23
7.22
7.22
7.22
7.21
7.21
7.21
7.20
7.19
2.78
2.77
2.77
2.77
2.76
2.76
2.76
2.75
2.75
2.66
2.65
2.65
2.65
2.64
2.64
2.64
2.63
2.63
1.97
1.96
1.95
1.95
1.94
1.89
1.88
1.87
1.87
1.86
1.86
0.03
O
9 a
2.00
2.00
2.22
2.24
2.37
2.27
7.5
7.0
6.5
6.0
5.5
5.0
4.5
4.0
3.5
3.0
2.5
2.0
1.5
1.0
0.5
0.0
f1 (ppm)

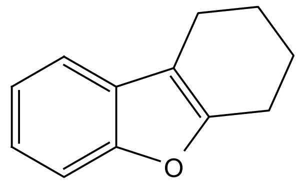

9a

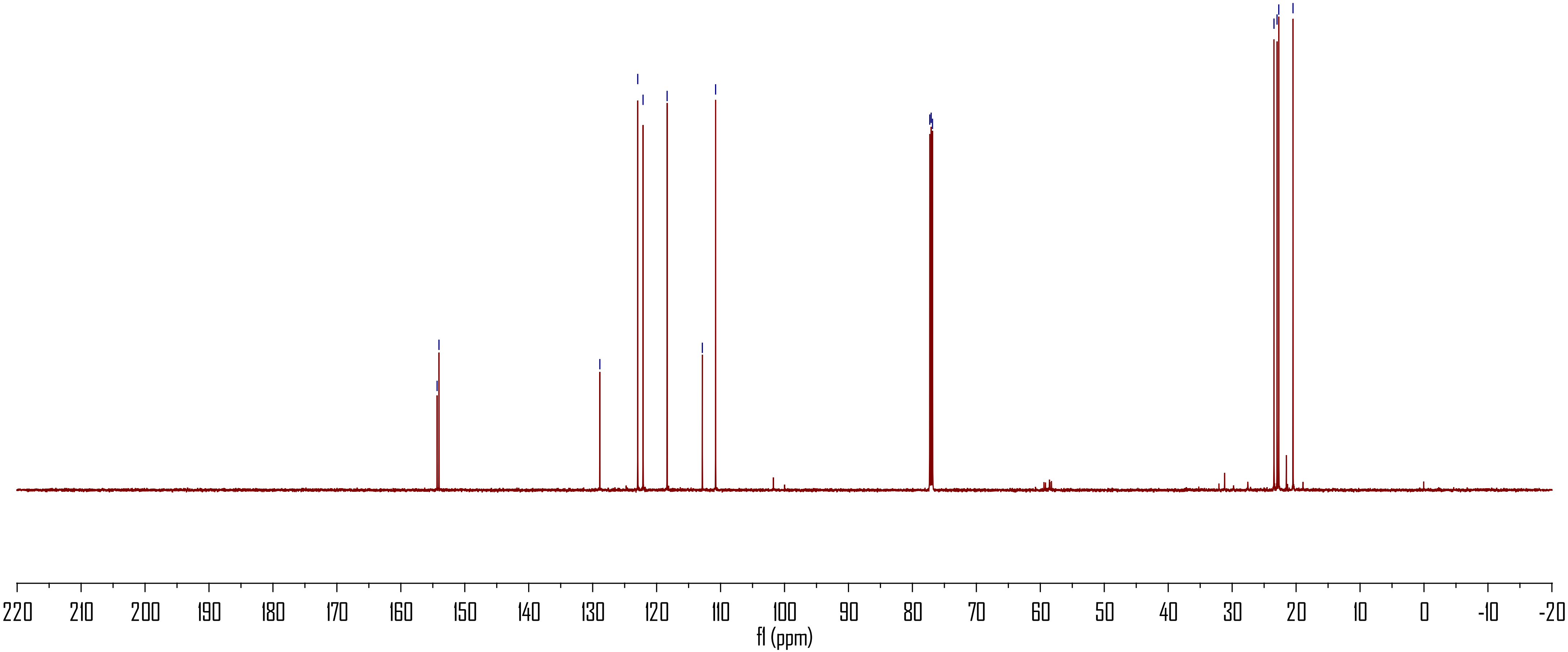

154.33
154.04
128.88
122.95
118.36
112.85
110.78
77.28
77.07
76.86
23.46
22.98
22.71
20.48
220 210 200 190 180 170 160 150 140 130 120 110 100 90 80 70 60 50 40 30 20 10 0 -10 -20
f1 (ppm)

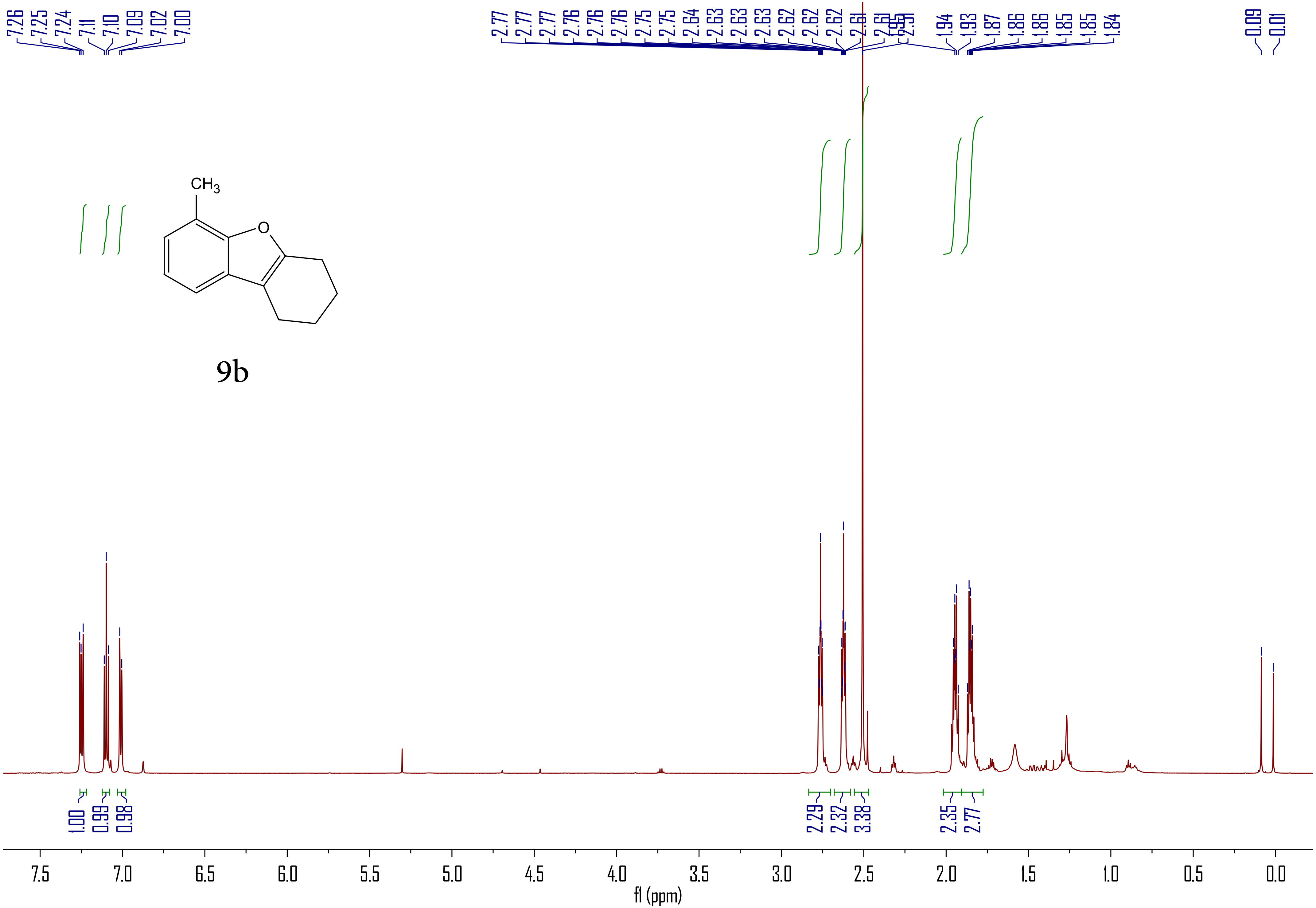

7.26
7.25
7.24
7.11
7.10
7.09
7.02
7.00
2.77
2.77
2.77
2.76
2.76
2.76
2.75
2.75
2.64
2.63
2.63
2.63
2.62
2.62
2.62
2.61
2.51
2.50
2.31
1.94
1.93
1.87
1.86
1.86
1.85
1.85
1.84
0.09
0.01
CH3
O
9b
1.00
0.99
0.98
2.29
2.32
3.38
2.35
2.77
7.5
7.0
6.5
6.0
5.5
5.0
4.5
4.0
3.5
3.0
2.5
2.0
1.5
1.0
0.5
0.0
f1 (ppm)

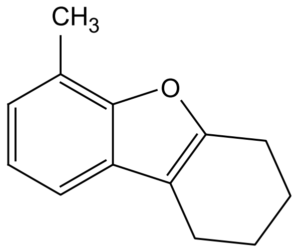
CH3
O

9b

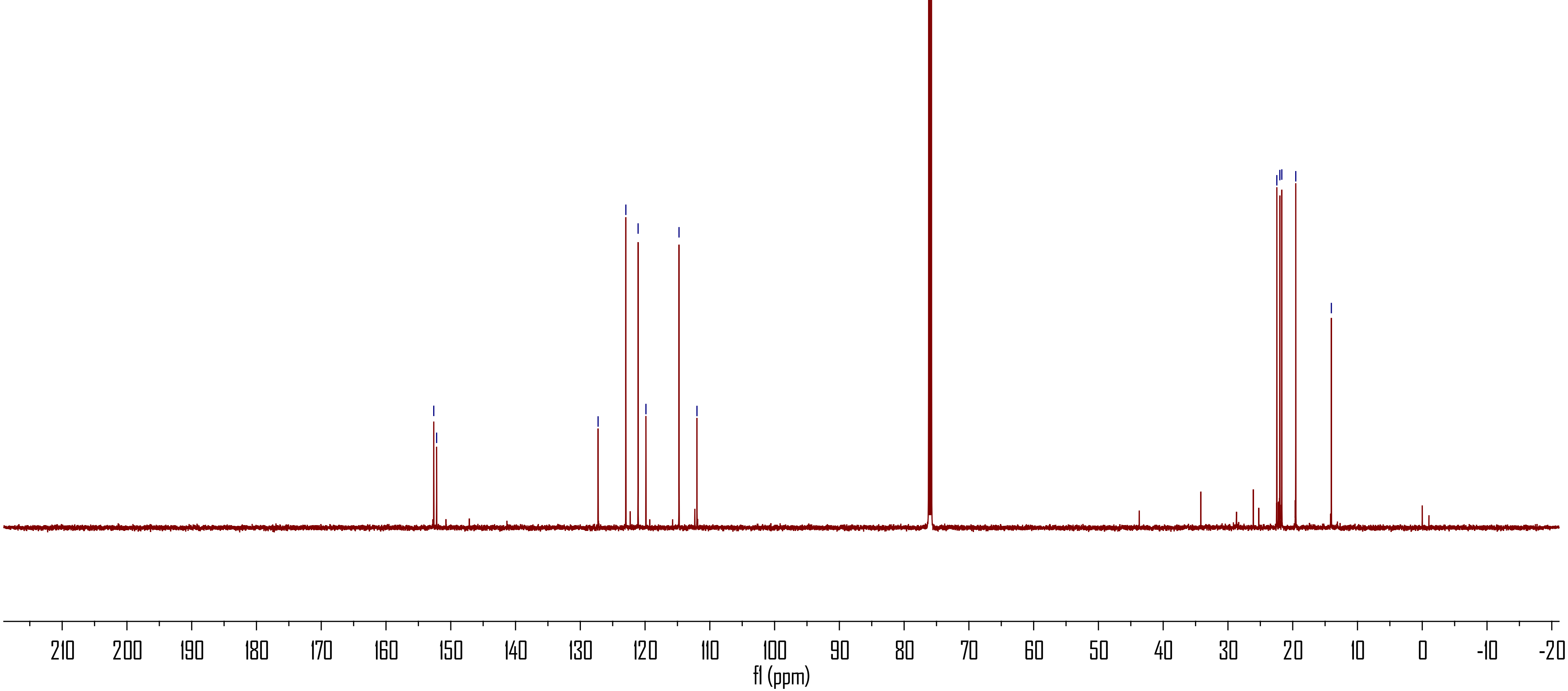
152.62
152.18
127.25
122.97
121.07
119.86
114.76
111.99
76.20
75.99
75.78
22.45
21.99
21.68
19.53
14.03
210
200
190
180
170
160
150
140
130
120
110
100
90
80
70
60
50
40
30
20
10
0
-10
-20
f1 (ppm)

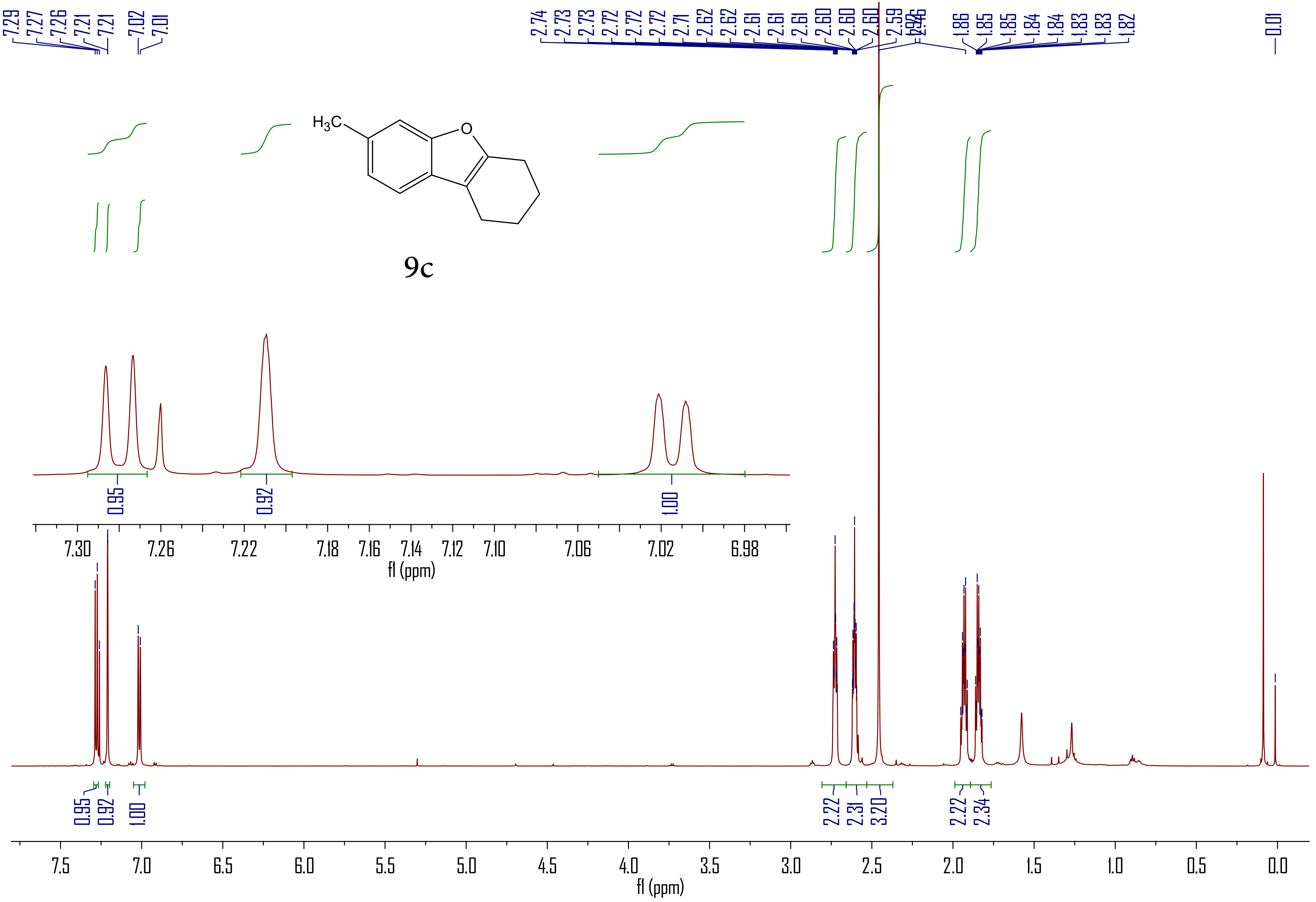

7.29
7.27
7.26
7.21
7.21
7.02
7.01
2.74
2.73
2.73
2.72
2.72
2.72
2.71
2.62
2.62
2.61
2.61
2.61
2.60
2.60
2.60
2.59
1.86
1.85
1.85
1.84
1.84
1.83
1.83
1.82
0.01
H_3C
O
9c
0.95
0.92
1.00
7.30
7.26
7.22
7.18
7.16
7.14
7.12
7.10
7.06
7.02
6.98
f1 (ppm)
2.22
2.31
3.20
2.22
2.34
7.5
7.0
6.5
6.0
5.5
5.0
4.5
4.0
3.5
3.0
2.5
2.0
1.5
1.0
0.5
0.0
f1 (ppm)

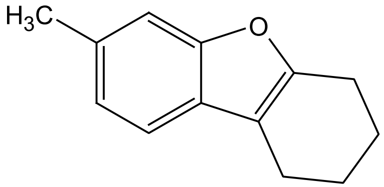
H3C
O

9c

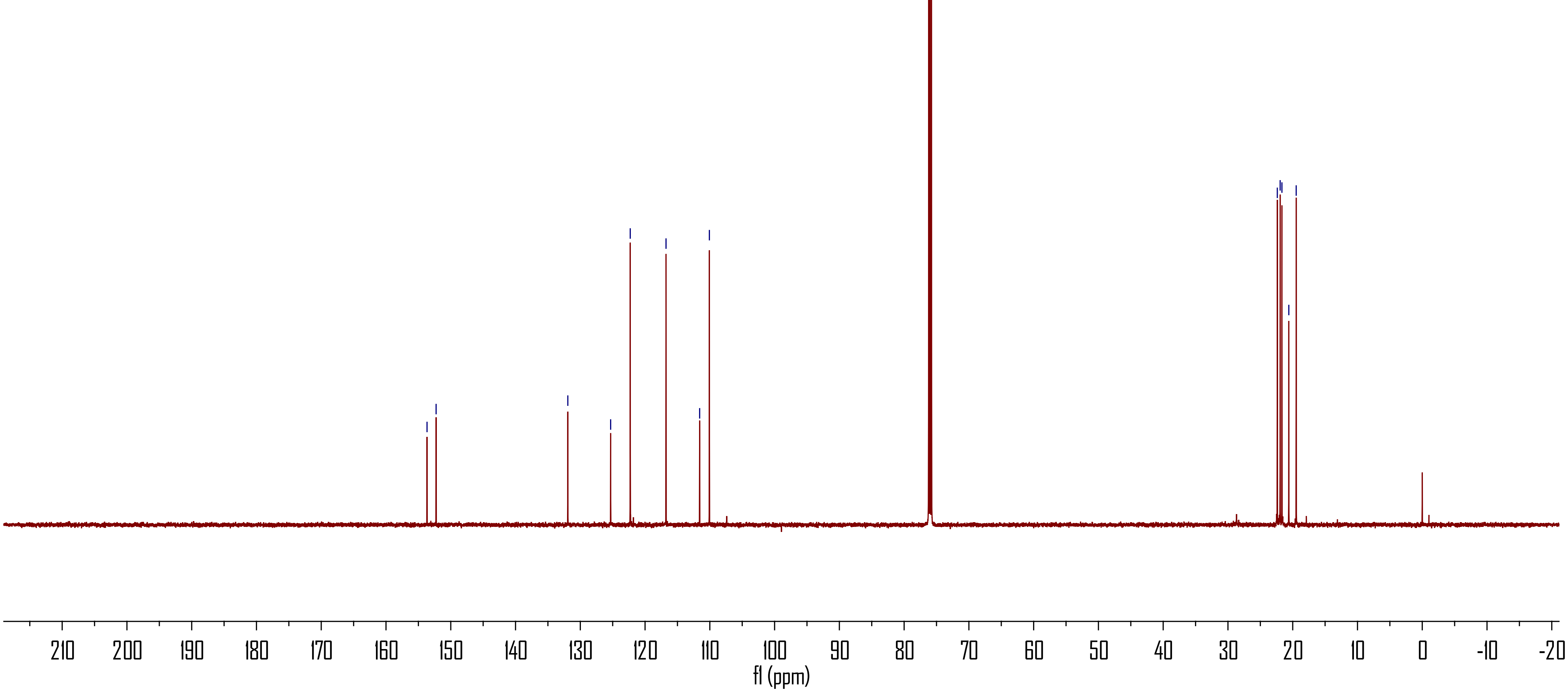
153.66
152.26
131.93
122.29
116.76
111.57
110.07
76.20
75.99
75.78
22.38
21.94
21.67
20.60
19.46
210
200
190
180
170
160
150
140
130
120
110
100
90
80
70
60
50
40
30
20
10
0
-10
-20
f1 (ppm)

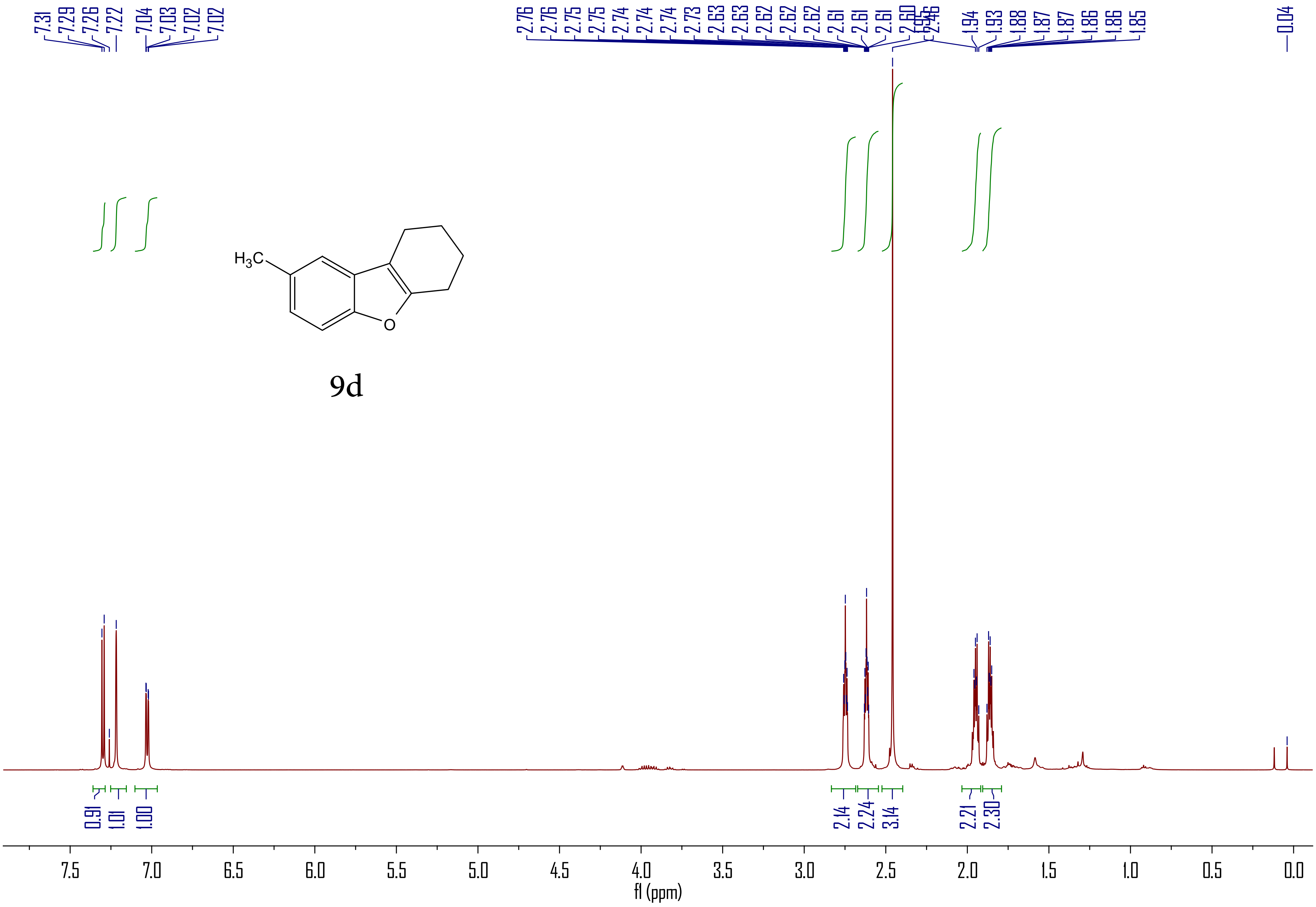

7.31
7.29
7.26
7.22
7.04
7.03
7.02
7.02
2.76
2.76
2.75
2.75
2.74
2.74
2.74
2.73
2.63
2.63
2.62
2.62
2.62
2.61
2.61
2.61
2.60
2.46
1.94
1.93
1.88
1.87
1.87
1.86
1.86
1.85
0.04
H_3C
O
9d
0.91
1.01
1.00
2.14
2.24
3.14
2.21
2.30
7.5
7.0
6.5
6.0
5.5
5.0
4.5
4.0
3.5
3.0
2.5
2.0
1.5
1.0
0.5
0.0
f1 (ppm)

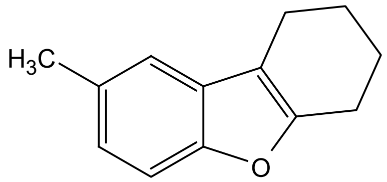
H_3C
O

9d

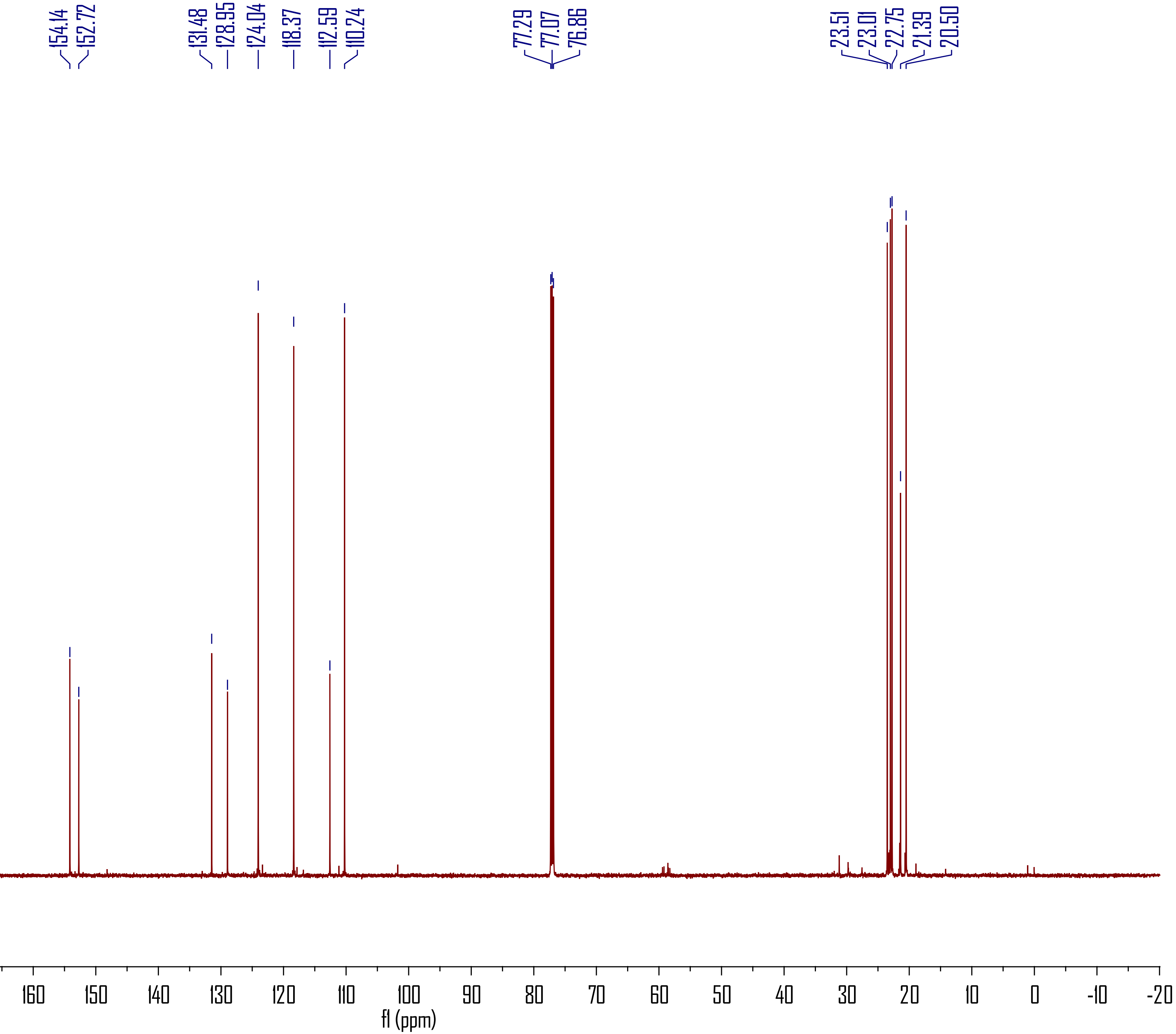
154.14
152.72
131.48
128.95
124.04
118.37
112.59
110.24
77.29
77.07
76.86
23.51
23.01
22.75
21.39
20.50
220 210 200 190 180 170 160 150 140 130 120 110 100 90 80 70 60 50 40 30 20 10 0 -10 -20
f1 (ppm)

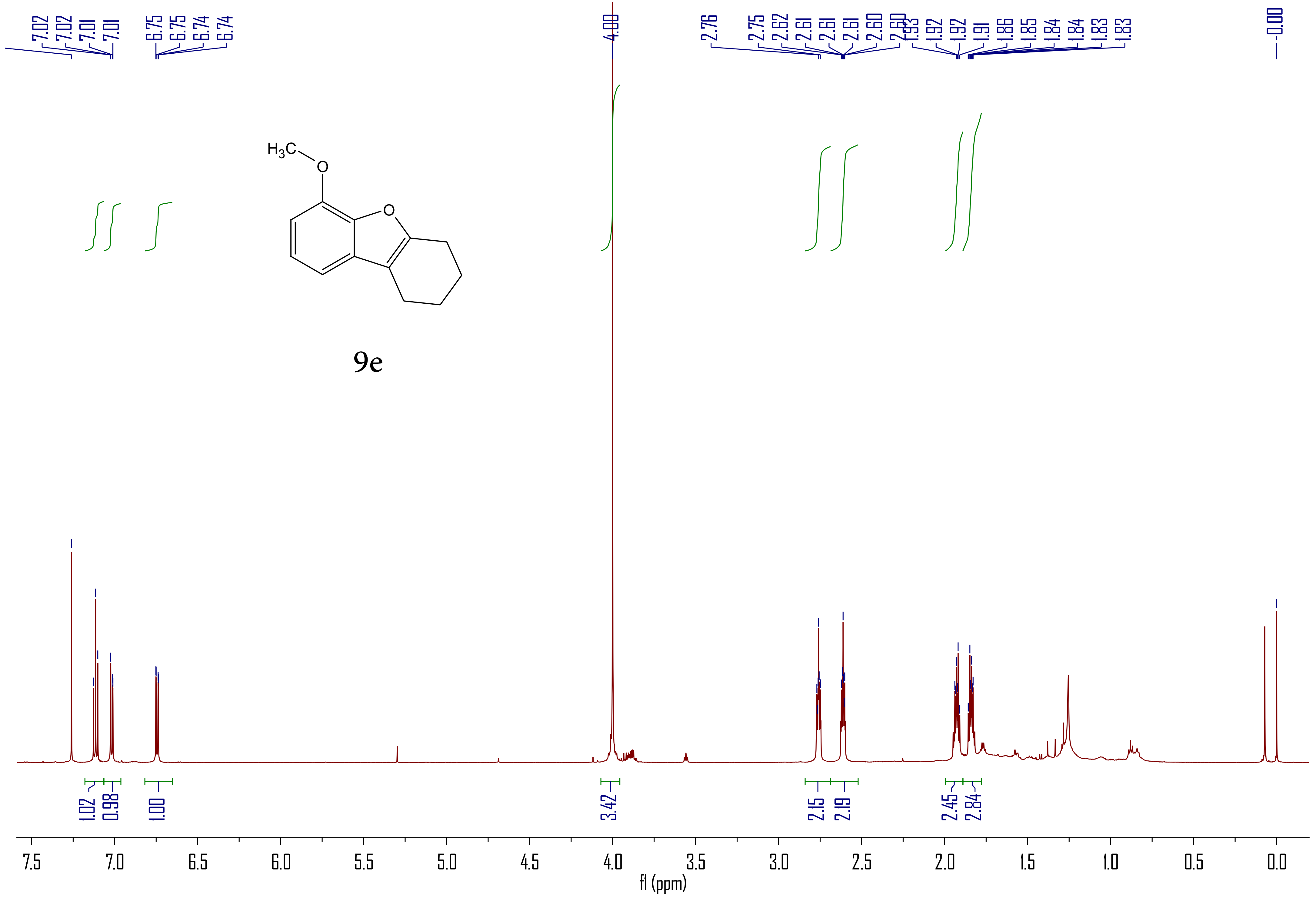

7.02
7.02
7.01
7.01
6.75
6.75
6.74
6.74
4.00
2.76
2.75
2.62
2.61
2.61
2.61
2.60
1.93
1.92
1.92
1.91
1.86
1.85
1.84
1.84
1.83
1.83
-0.00
H3C
O
O
9e
1.02
0.98
1.00
3.42
2.15
2.19
2.45
2.84
7.5
7.0
6.5
6.0
5.5
5.0
4.5
4.0
3.5
3.0
2.5
2.0
1.5
1.0
0.5
0.0
f1 (ppm)

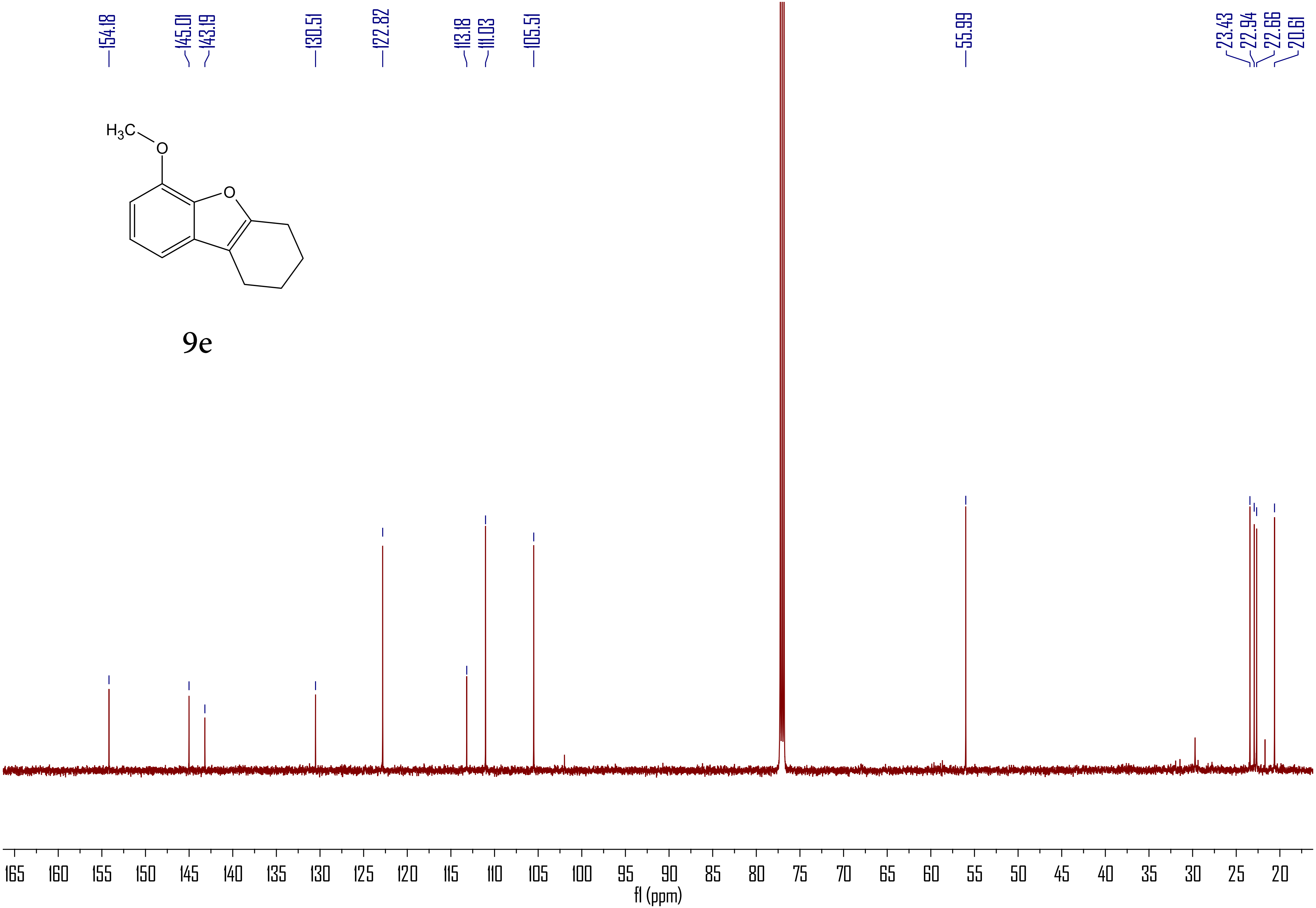
154.18
145.01
143.19
130.51
122.82
113.18
111.03
105.51
55.99
23.43
22.94
22.66
20.61
H3C
O
O
9e
165 160 155 150 145 140 135 130 125 120 115 110 105 100 95 90 85 80 75 70 65 60 55 50 45 40 35 30 25 20
f1 (ppm)

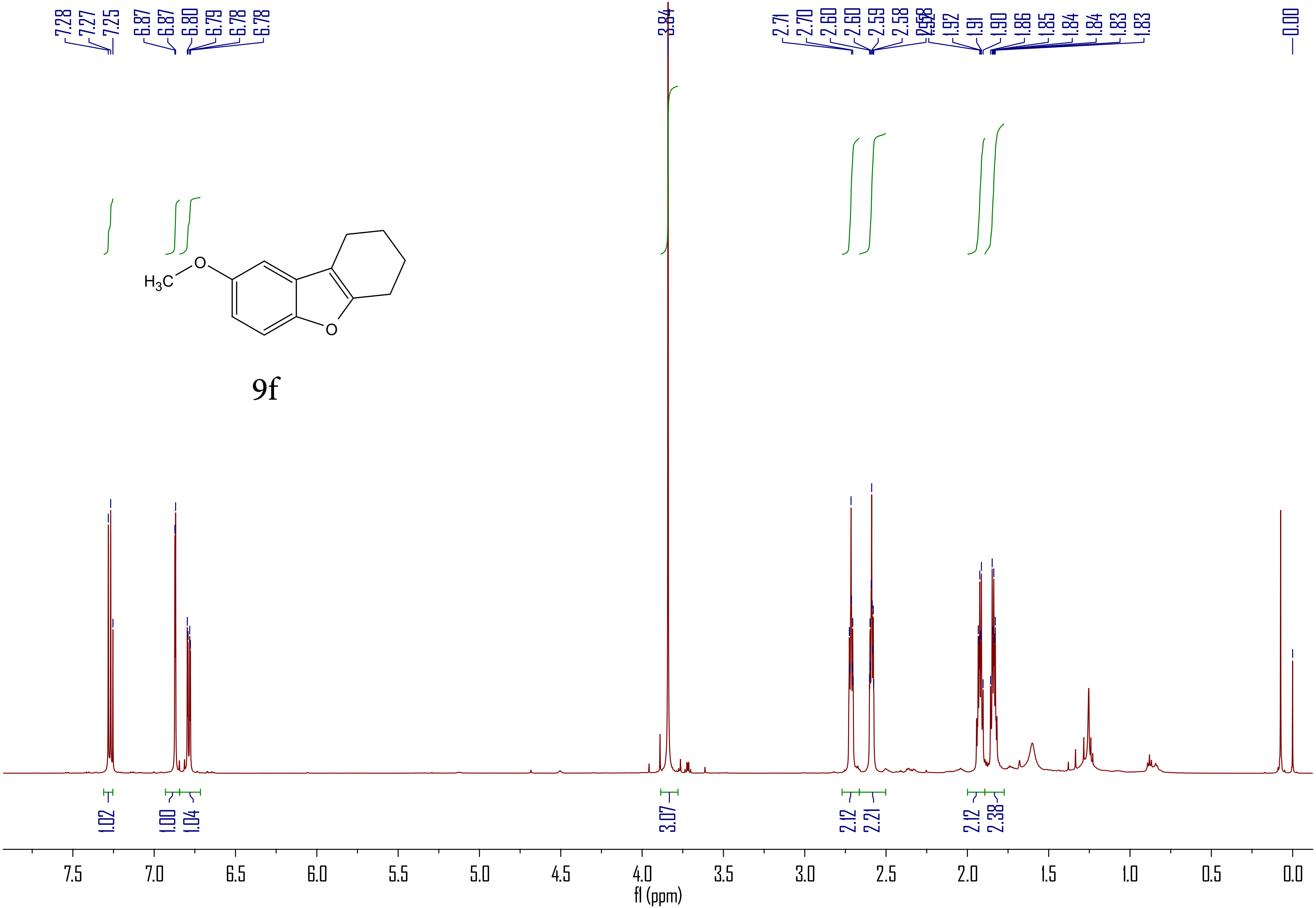
7.28
7.27
7.25
6.87
6.87
6.80
6.79
6.78
6.78
3.84
2.71
2.70
2.60
2.60
2.59
2.58
1.92
1.91
1.90
1.86
1.85
1.84
1.84
1.83
1.83
0.00
H3C
O
O
9f
1.02
1.00
1.04
3.07
2.12
2.21
2.12
2.38
7.5
7.0
6.5
6.0
5.5
5.0
4.5
4.0
3.5
3.0
2.5
2.0
1.5
1.0
0.5
0.0
f1 (ppm)

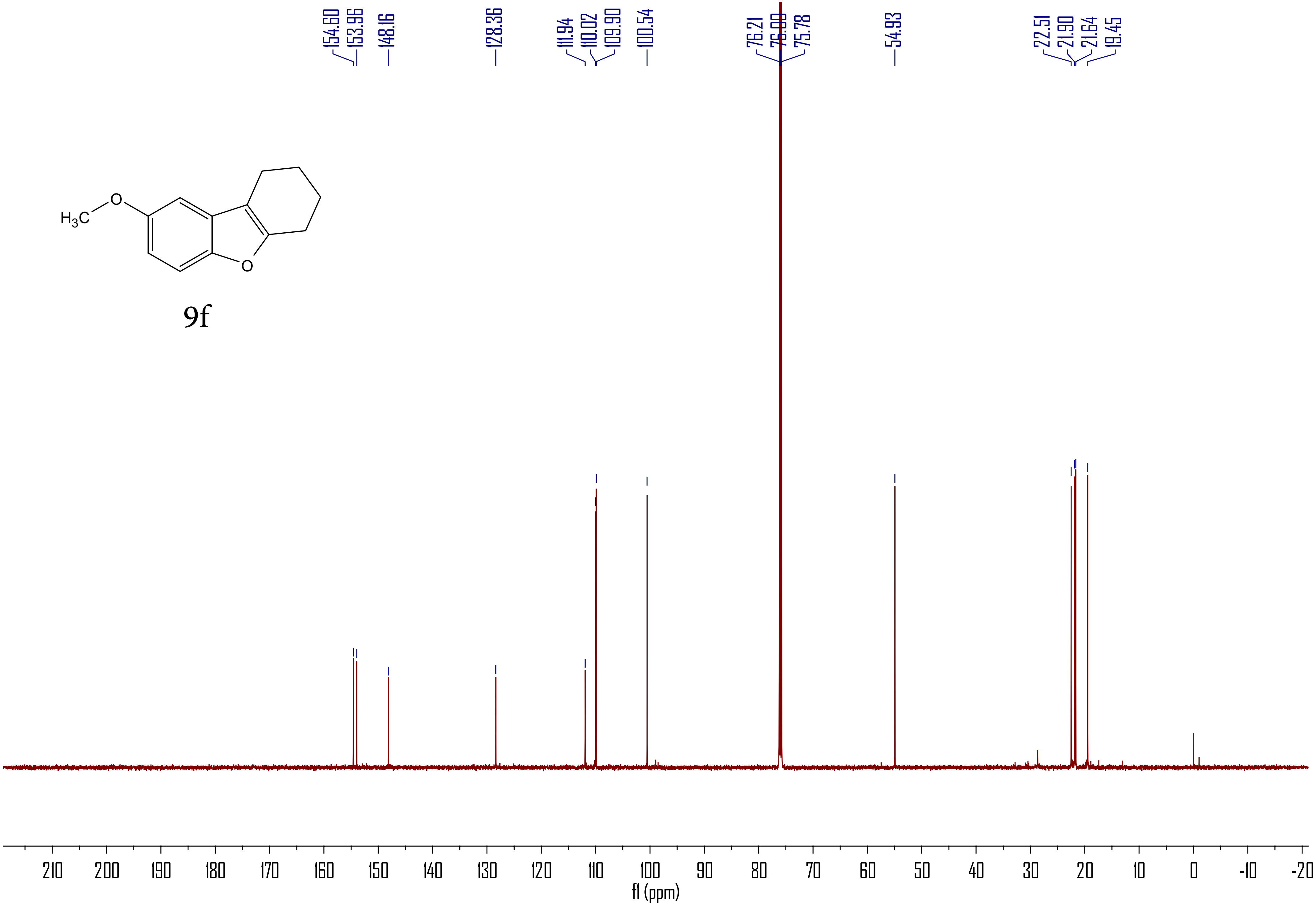

154.60
153.96
148.16
128.36
111.94
110.02
109.90
100.54
76.21
76.00
75.78
54.93
22.51
21.90
21.64
19.45
H3C
O
O
9f
210
200
190
180
170
160
150
140
130
120
110
100
90
80
70
60
50
40
30
20
10
0
-10
-20
f1 (ppm)

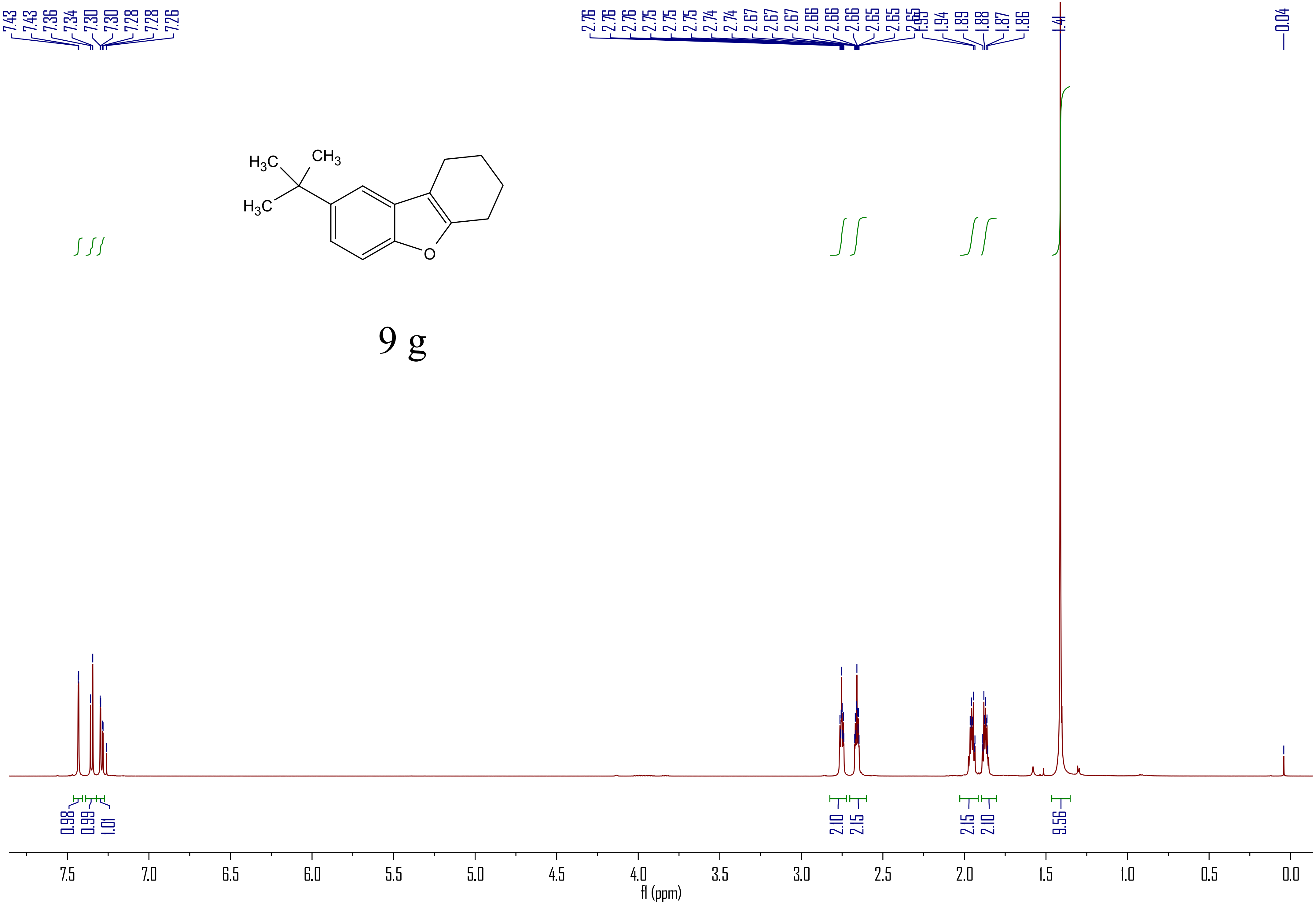

7.43
7.43
7.36
7.34
7.30
7.30
7.28
7.28
7.26
2.76
2.76
2.76
2.75
2.75
2.75
2.74
2.74
2.67
2.67
2.67
2.66
2.66
2.66
2.65
2.65
1.94
1.89
1.88
1.87
1.86
1.41
0.04
H_3C
CH_3
H_3C
O
9 g
0.98
0.99
1.01
2.10
2.15
2.15
2.10
9.56
7.5
7.0
6.5
6.0
5.5
5.0
4.5
4.0
3.5
3.0
2.5
2.0
1.5
1.0
0.5
0.0
f1 (ppm)

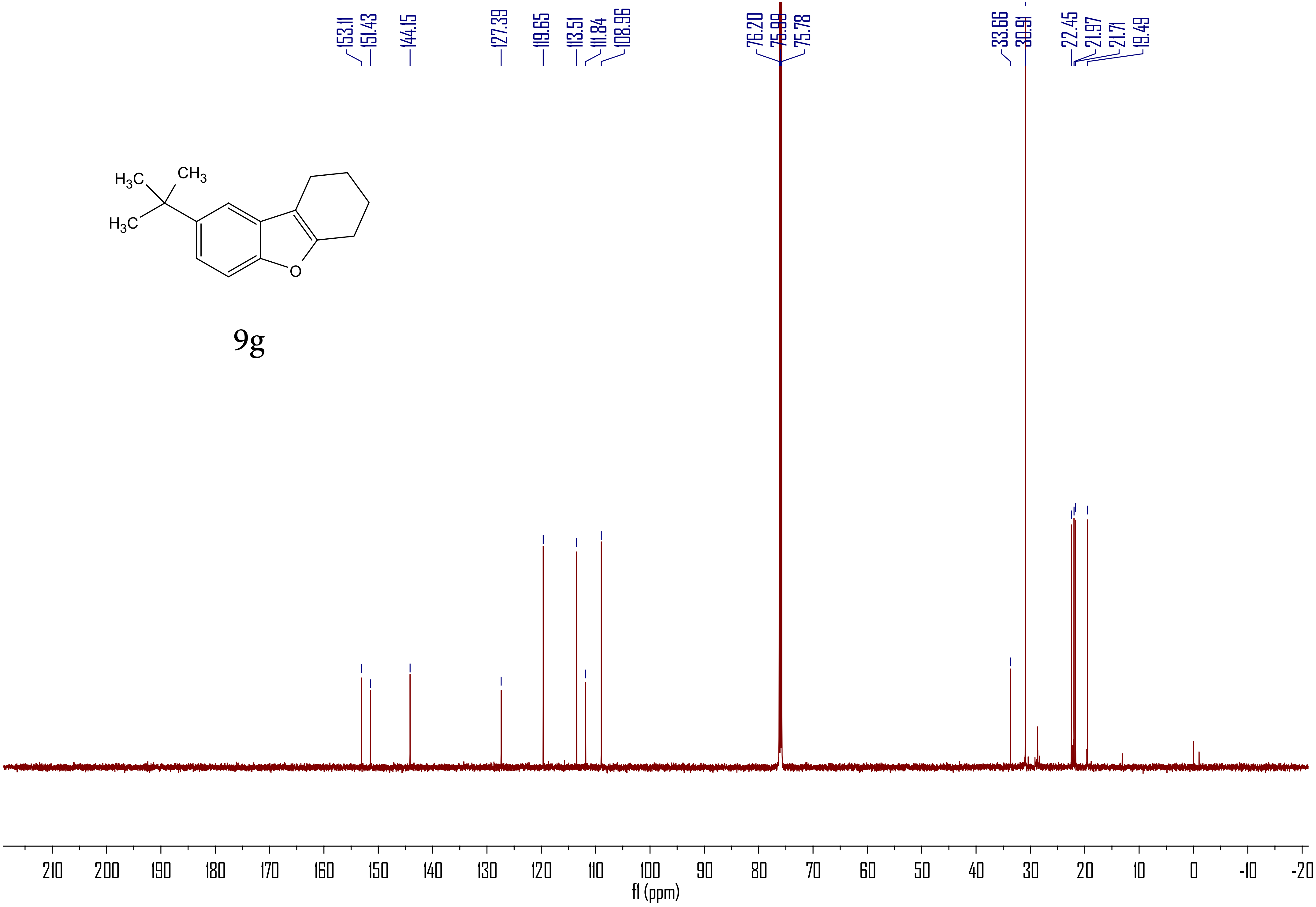

H3C
CH3
H3C
O
153.11
151.43
144.15
127.39
119.65
113.51
111.84
108.96
76.20
75.78
33.66
30.91
22.45
21.97
21.71
19.49
210
200
190
180
170
160
150
140
130
120
110
100
90
80
70
60
50
40
30
20
10
0
-10
-20
f1 (ppm)

9g

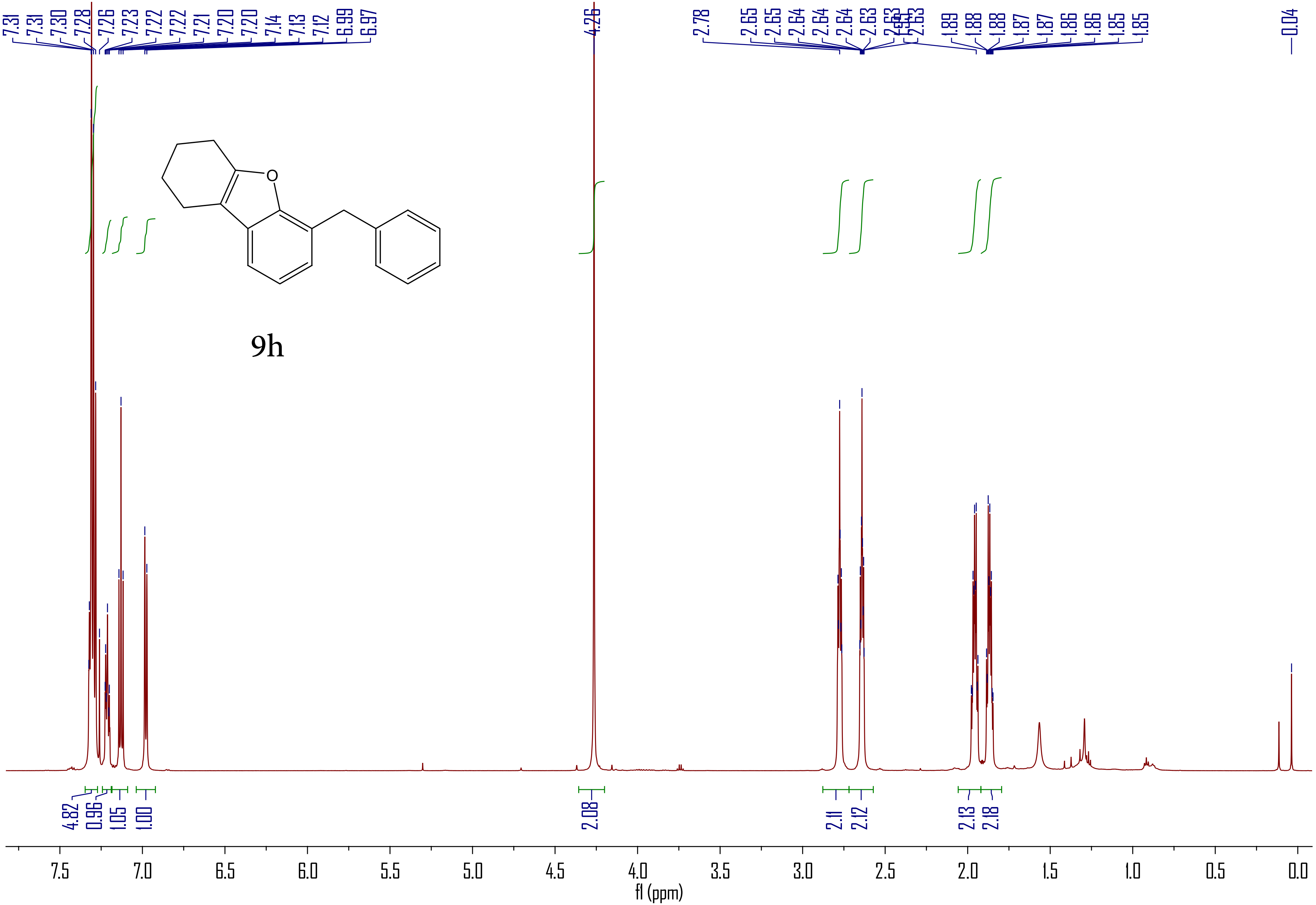
7.31
7.31
7.30
7.28
7.26
7.23
7.22
7.22
7.21
7.20
7.20
7.14
7.13
7.12
6.99
6.97
4.26
2.78
2.65
2.65
2.64
2.64
2.64
2.63
2.63
2.63
1.89
1.88
1.88
1.87
1.87
1.86
1.86
1.85
1.85
0.04
O
9h
4.82
0.96
1.05
1.00
2.08
2.11
2.12
2.13
2.18
7.5
7.0
6.5
6.0
5.5
5.0
4.5
4.0
3.5
3.0
2.5
2.0
1.5
1.0
0.5
0.0
f1 (ppm)

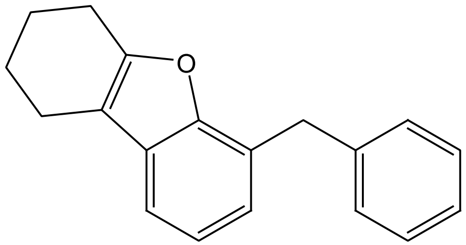

9h

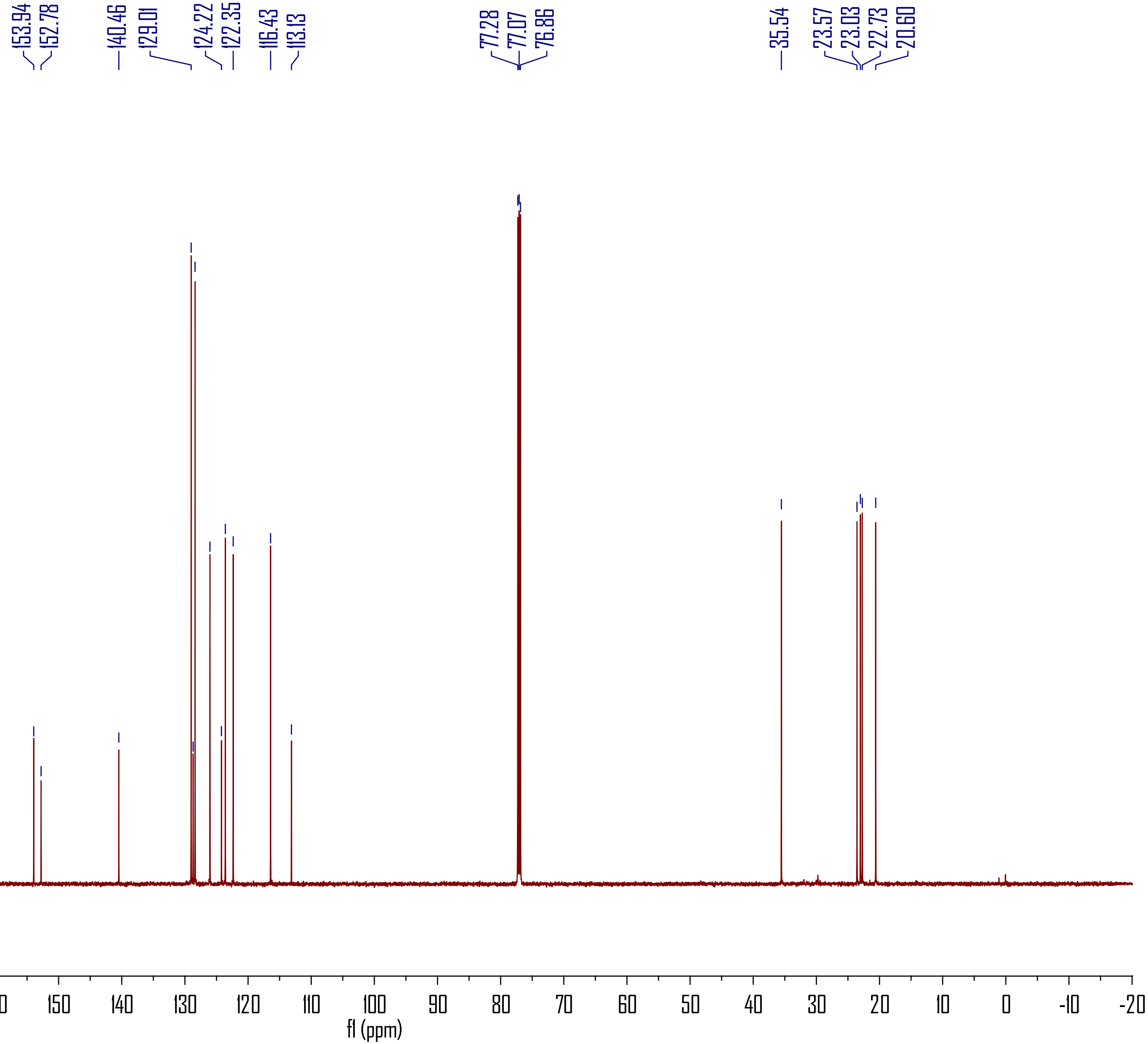

153.94
152.78
140.46
129.01
124.22
122.35
116.43
113.13
77.28
77.07
76.86
35.54
23.57
23.03
22.73
20.60
220 210 200 190 180 170 160 150 140 130 120 110 100 90 80 70 60 50 40 30 20 10 0 -10 -20
f1 (ppm)

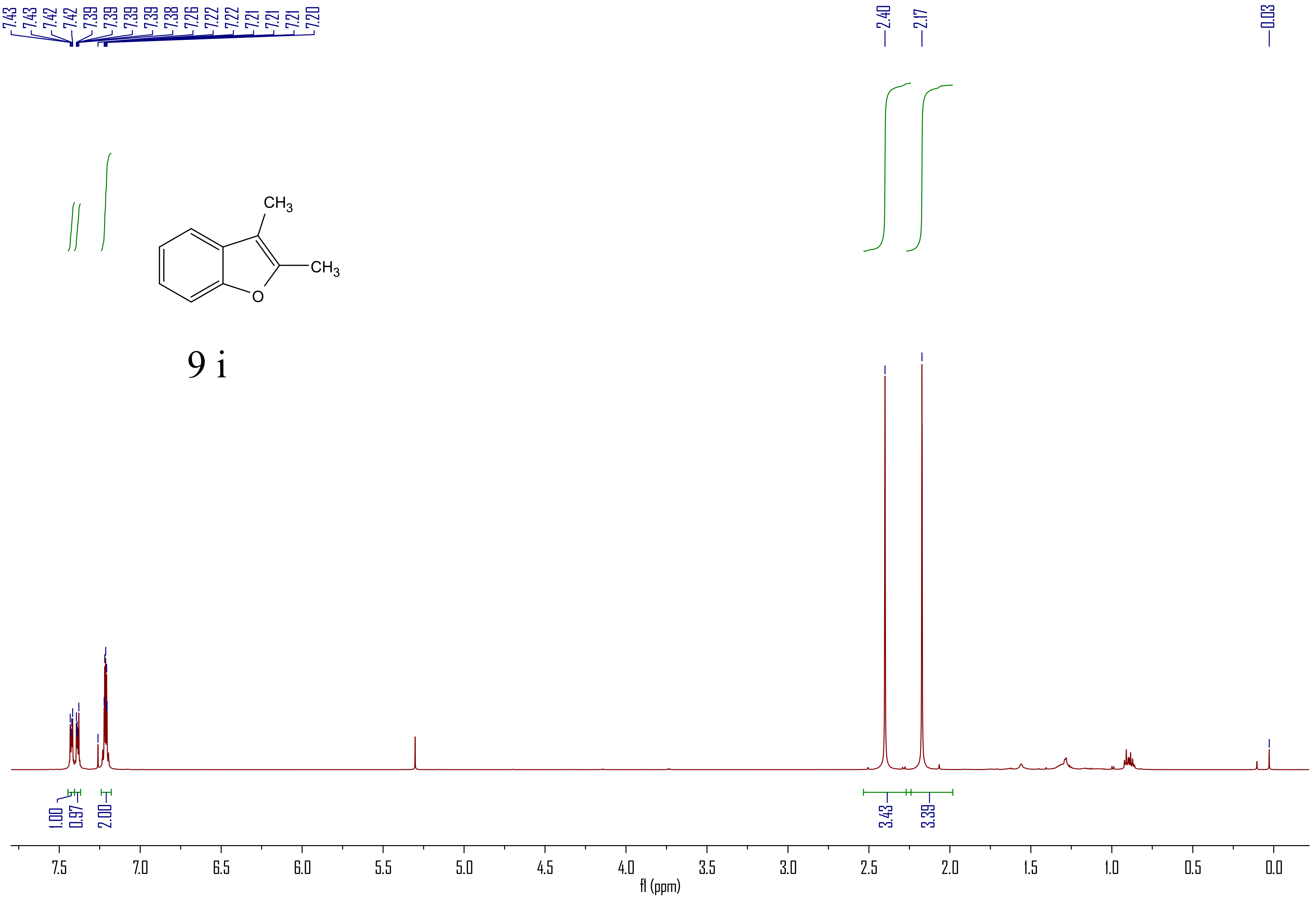
7.43
7.43
7.42
7.42
7.39
7.39
7.39
7.39
7.38
7.26
7.22
7.22
7.21
7.21
7.21
7.20
2.40
2.17
0.03
CH_3
CH_3
O
9 i
1.00
0.97
2.00
3.43
3.39
7.5
7.0
6.5
6.0
5.5
5.0
4.5
4.0
3.5
3.0
2.5
2.0
1.5
1.0
0.5
0.0
f1 (ppm)

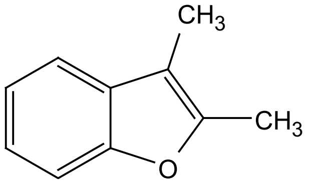
CH3
CH3
O

9i

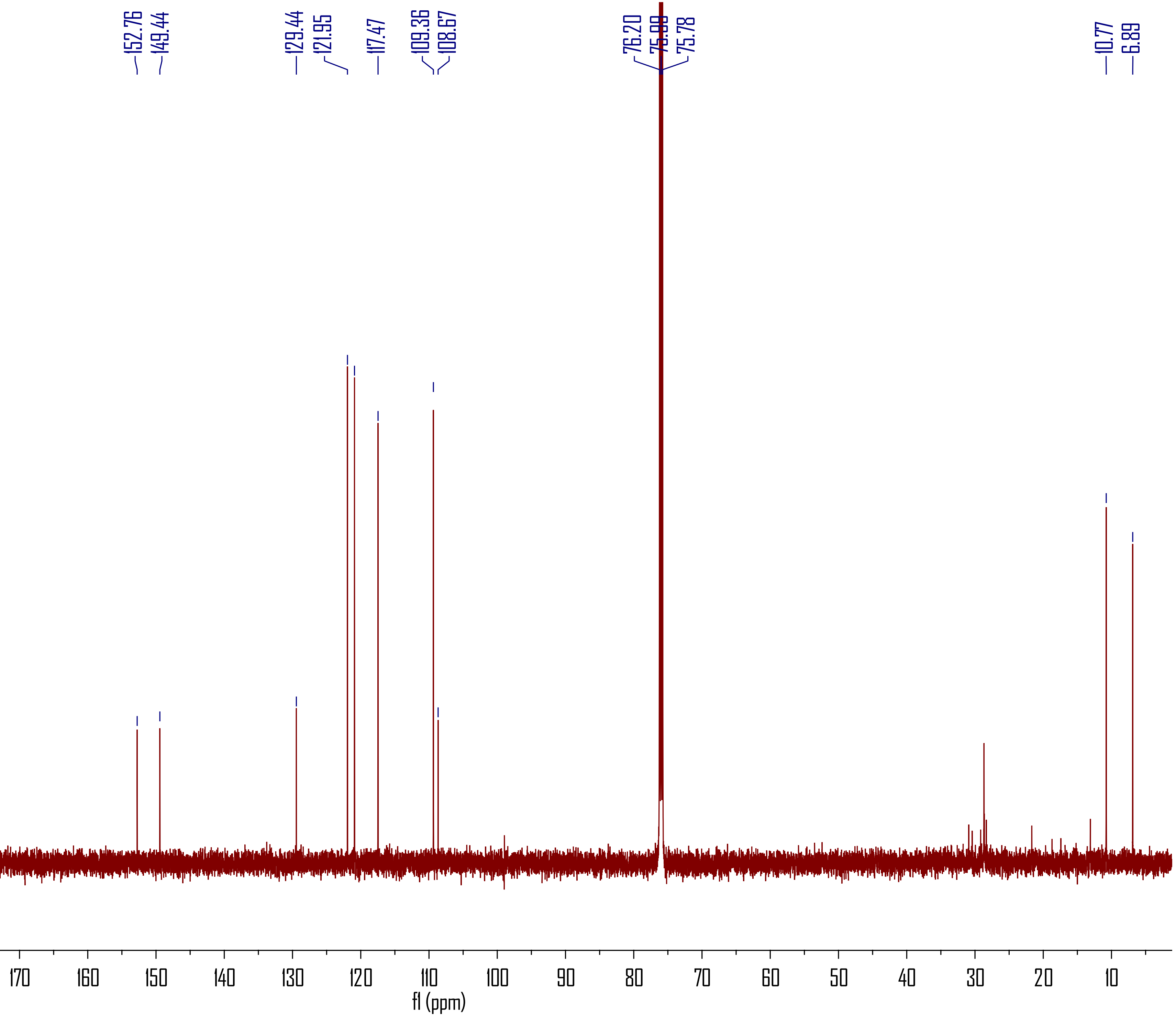
152.76
149.44
129.44
121.95
117.47
109.36
108.67
76.20
75.78
10.77
6.89
210
200
190
180
170
160
150
140
130
120
110
100
90
80
70
60
50
40
30
20
10
f1 (ppm)

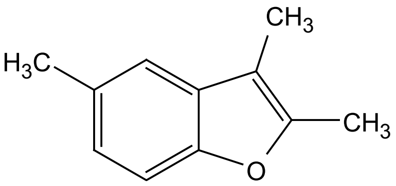
CH3
H3C
CH3
O

9j

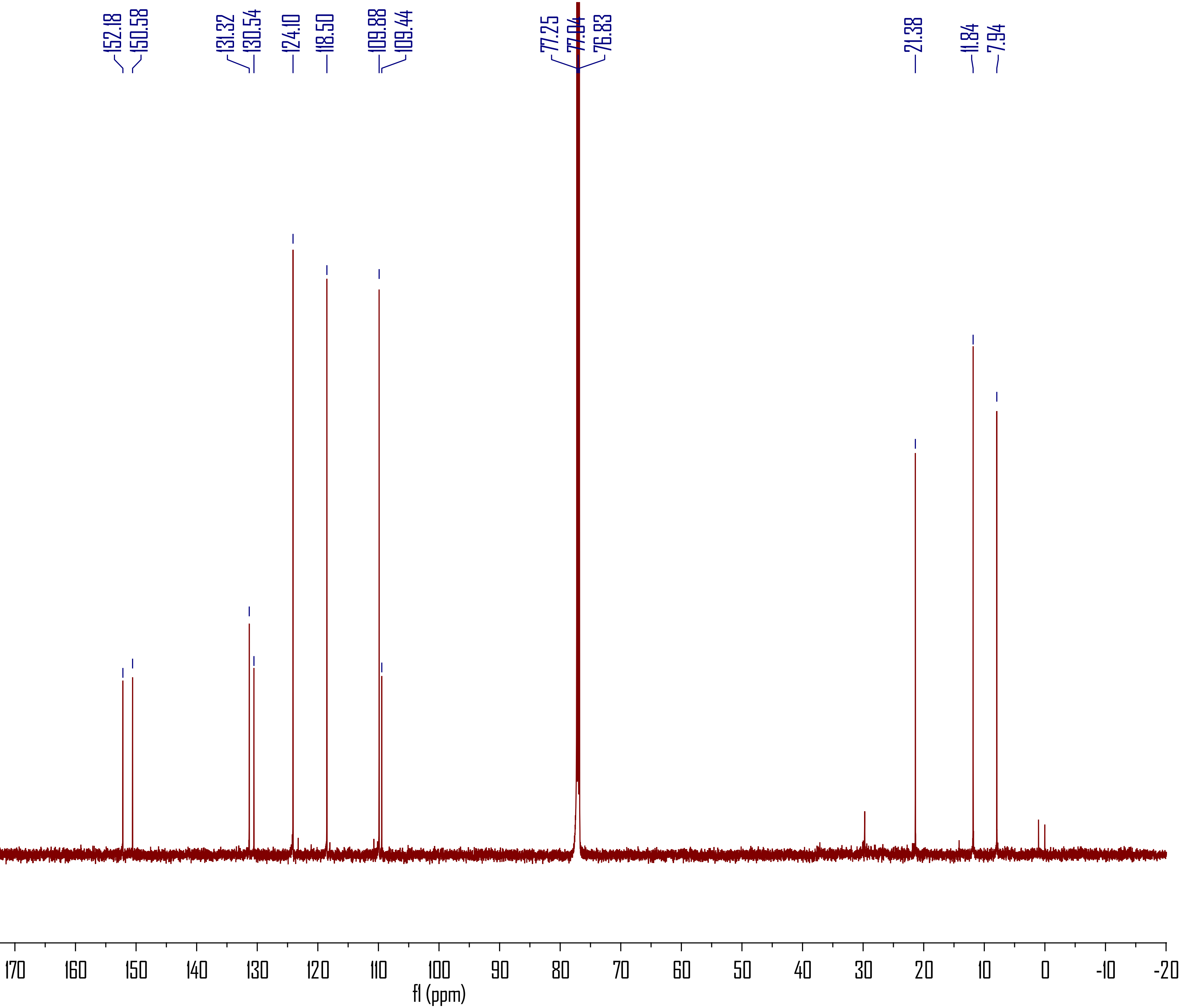
152.18
150.58
131.32
130.54
124.10
118.50
109.88
109.44
77.25
77.04
76.83
21.38
11.84
7.94
220 210 200 190 180 170 160 150 140 130 120 110 100 90 80 70 60 50 40 30 20 10 0 -10 -20
f1 (ppm)

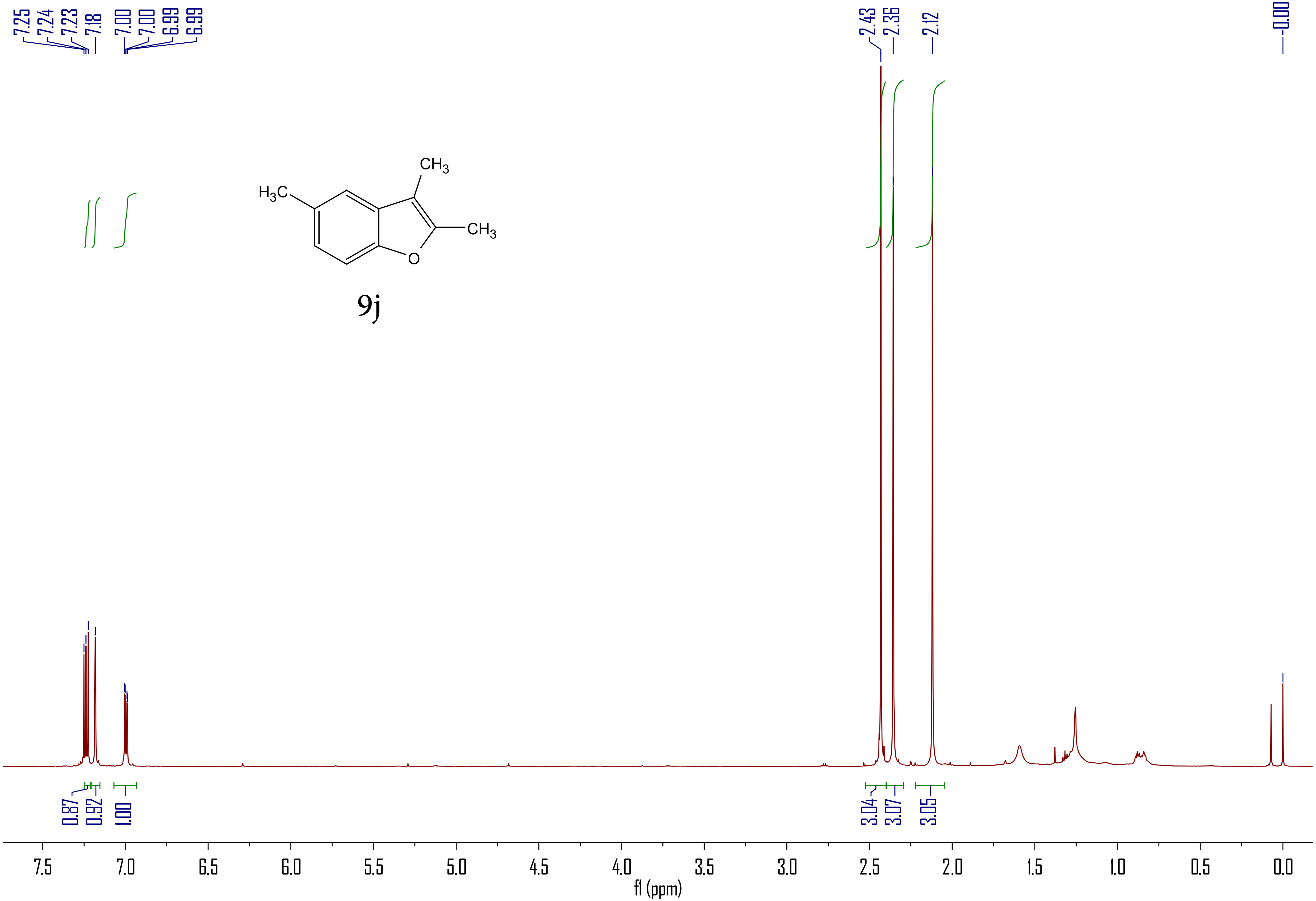
7.25
7.24
7.23
7.18
7.00
7.00
6.99
6.99
2.43
2.36
2.12
-0.00
CH3
H3C
CH3
O
9j
0.87
0.92
1.00
3.04
3.07
3.05
7.5
7.0
6.5
6.0
5.5
5.0
4.5
4.0
3.5
3.0
2.5
2.0
1.5
1.0
0.5
0.0
f1 (ppm)

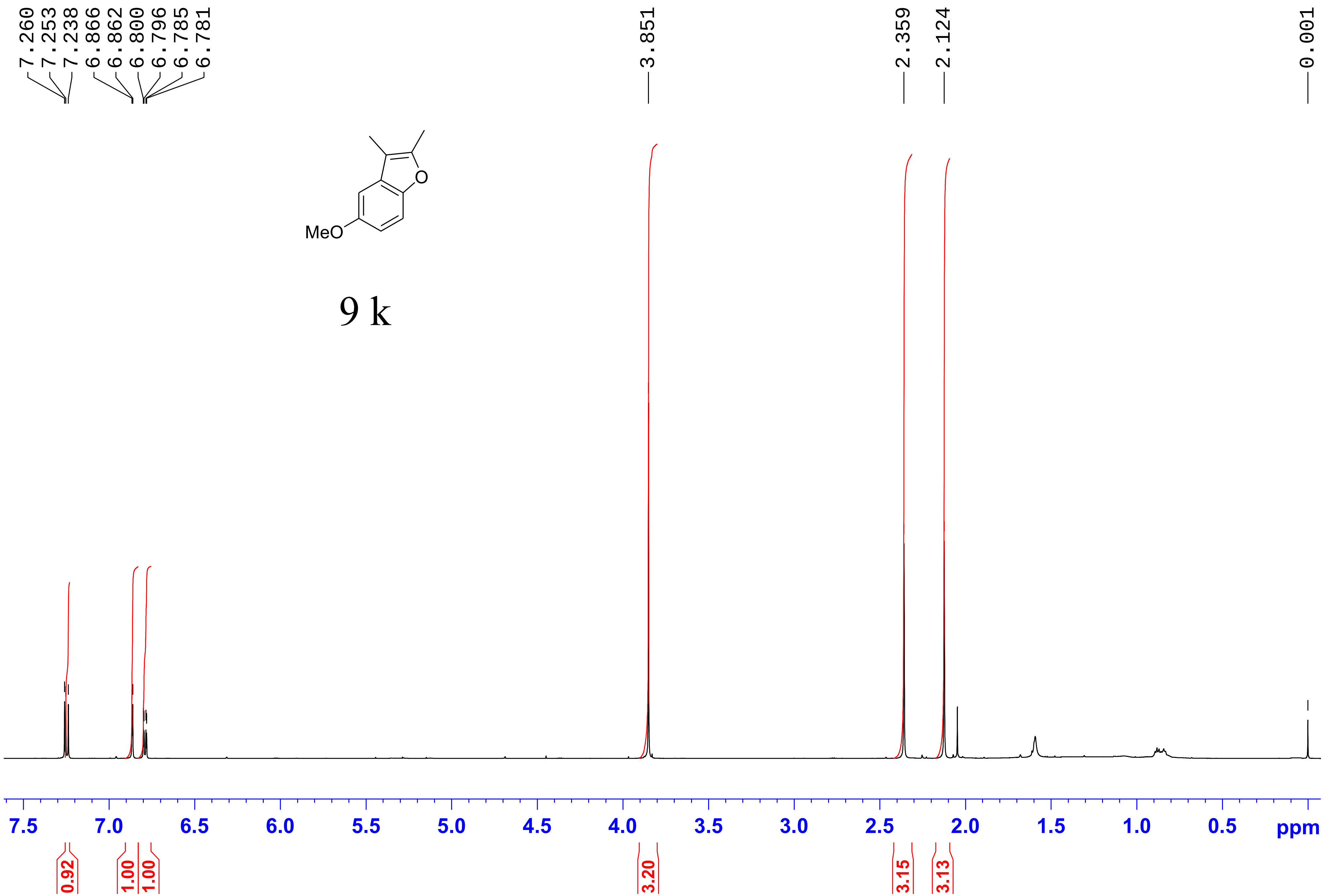
7.260
7.253
7.238
6.866
6.862
6.800
6.796
6.785
6.781
3.851
2.359
2.124
0.001
MeO
O
9 k
7.5
7.0
6.5
6.0
5.5
5.0
4.5
4.0
3.5
3.0
2.5
2.0
1.5
1.0
0.5
ppm
0.92
1.00
1.00
3.20
3.15
3.13

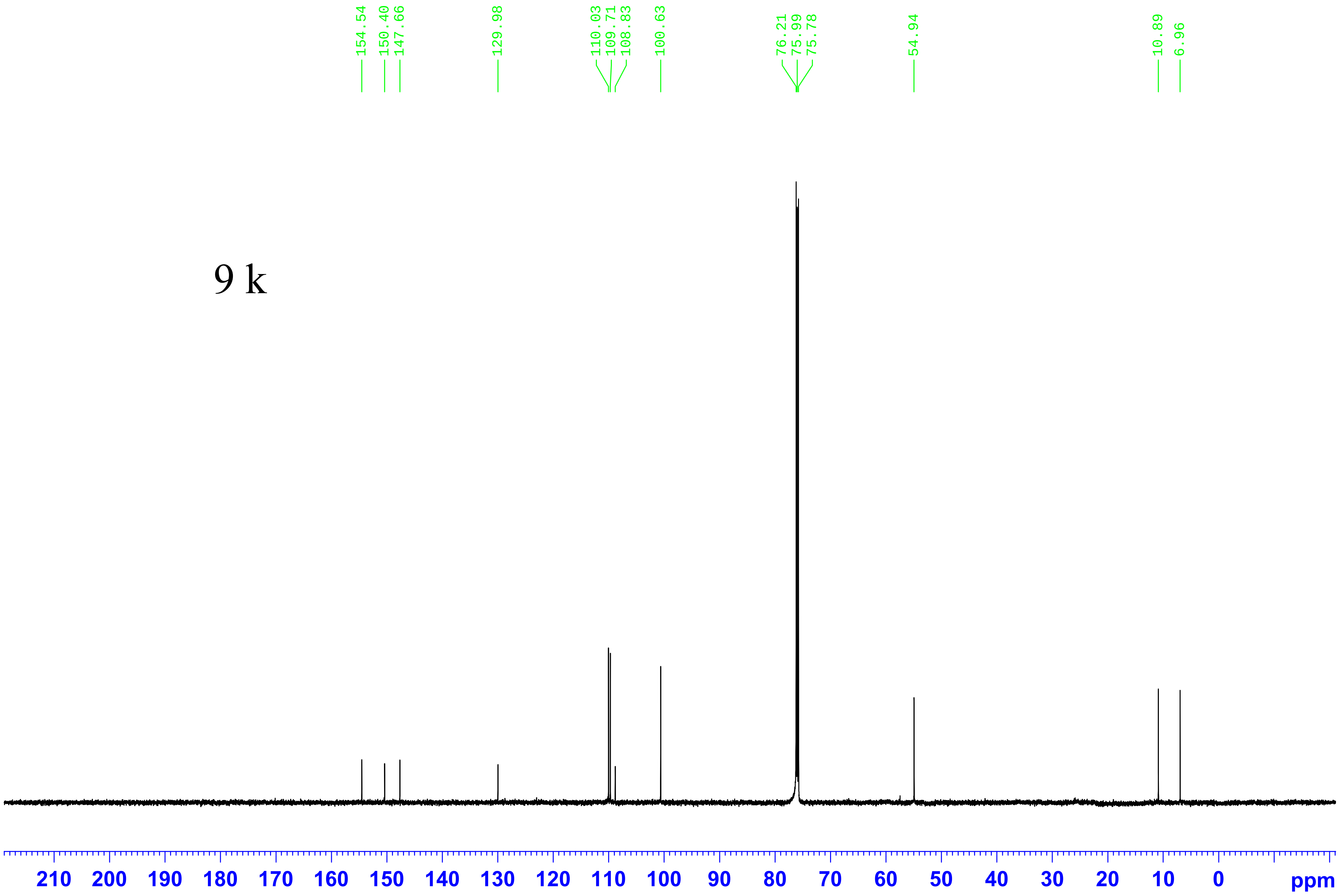
154.54
150.40
147.66
129.98
110.03
109.71
108.83
100.63
76.21
75.99
75.78
54.94
10.89
6.96
9 k
210
200
190
180
170
160
150
140
130
120
110
100
90
80
70
60
50
40
30
20
10
0
ppm
